# Suicide rates amongst individuals from ethnic minority backgrounds: A systematic review and meta-analysis

**DOI:** 10.1016/j.eclinm.2022.101399

**Published:** 2022-04-28

**Authors:** M.Isabela Troya, Matthew J. Spittal, Rosina Pendrous, Grace Crowley, Hayley C Gorton, Kirsten Russell, Sadhbh Byrne, Rebecca Musgrove, Stephanie Hannah-Swain, Navneet Kapur, Duleeka Knipe

**Affiliations:** a School of Public Health, College of Medicine and Health, University College Cork, 4.07 Western Gateway Building, Cork, Ireland; b National Suicide Research Foundation, University College Cork, 4.28 Western Gateway Building, Cork, Ireland; c Centre for Mental Health, Melbourne School of Population and Global Health, University of Melbourne, Melbourne, Australia; d School of Psychology, University of Chester, Chester, UK; e Population Health Sciences, Bristol Medical School, University of Bristol, Bristol, UK; f Department of Pharmacy, School of Applied Sciences, University of Huddersfield, Huddersfield, UK; g School of Psychological Sciences and Health, Graham Hills Building, 40 George Street, Glasgow, UK; h Trinity Centre for Global Health, Trinity College Dublin, Dublin, Ireland; i Centre for Mental Health and Safety, National Institute for Health Research Greater Manchester Patient Safety Translational Research Centre, Manchester Academic Health Sciences Centre, University of Manchester, and Greater Manchester Mental Health NHS Foundation Trust, Manchester, UK; j Sheffield Hallam University, Sheffield, UK; k Division of Psychology and Mental Health, Centre for Mental Health and Safety, The University of Manchester, Manchester, UK

**Keywords:** Suicide, Self-harm, Ethnic minorities, Indigenous, Migrant, Refugee, Asylum seeker

## Abstract

**Background:**

Existing evidence suggests that some individuals from ethnic minority backgrounds are at increased risk of suicide compared to their majority ethnic counterparts, whereas others are at decreased risk. We aimed to estimate the absolute and relative risk of suicide in individuals from ethnic minority backgrounds globally.

**Methods:**

Databases (Medline, Embase, and PsycInfo) were searched for epidemiological studies between 01/01/2000 and 3/07/2020, which provided data on absolute and relative rates of suicide amongst ethnic minority groups. Studies reporting on clinical or specific populations were excluded. Pairs of reviewers independently screened titles, abstracts, and full texts. We used random effects meta-analysis to estimate overall, sex, location, migrant status, and ancestral origin, stratified pooled estimates for absolute and rate ratios. PROSPERO registration: CRD42020197940.

**Findings:**

A total of 128 studies were included with 6,026,103 suicide deaths in individuals from an ethnic minority background across 31 countries. Using data from 42 moderate-high quality studies, we estimated a pooled suicide rate of 12·1 per 100,000 (95% CIs 8·4–17·6) in people from ethnic minority backgrounds with a broad range of estimates (1·2–139·7 per 100,000). There was weak statistical evidence from 51 moderate-high quality studies that individuals from ethnic minority groups were more likely to die by suicide (RR 1·3 95% CIs 0·9–1·7) with again a broad range amongst studies (RR 0·2–18·5). In our sub-group analysis we only found evidence of elevated risk for indigenous populations (RR: 2·8 95% CIs 1·9–4·0; pooled rate: 23·2 per 100,000 95% CIs 14·7–36·6). There was very substantial heterogeneity (*I^2^* > 98%) between studies for all pooled estimates.

**Interpretation:**

The homogeneous grouping of individuals from ethnic minority backgrounds is inappropriate. To support suicide prevention in marginalised groups, further exploration of important contextual differences in risk is required. It is possible that some ethnic minority groups (for example those from indigenous backgrounds) have higher rates of suicide than majority populations.

**Funding:**

No specific funding was provided to conduct this research. DK is funded by Wellcome Trust and Elizabeth Blackwell Institute Bristol. Matthew Spittal is a recipient of an Australian Research Council Future Fellowship (project number FT180100075) funded by the Australian Government. Rebecca Musgrove is funded by the NIHR Greater Manchester Patient Safety Translational Research Centre (PSTRC-2016-003).

**Research in context**

**Evidence before this study**

Suicidal behaviour is a major public health concern and variation in suicidal behaviour is likely to be observed between culturally distinct populations within nations. We searched Medline, Embase, and PsycINFO for systematic reviews published between January 1, 2011 and July 21, 2021, and the search terms included: (ethnic minority OR migrant OR refugee OR expatriate OR asylum-seeker OR indigenous OR departee OR foreign born OR foreign worker OR foreign student OR international student, OR minority group OR ethnic group OR BME OR BAME OR CALD OR cultural diversity) AND (suicide OR self-harm) AND (systematic review), and found a total of 265 records  and seven relevant systematic reviews, however these were restricted to specific groups (*n* = 3) (e.g. migrants or indigenous groups only) or to a specific area (*n* = 4) (e.g. Latin America). We found no previous systematic review which aimed to identify and synthesise original research to estimate the absolute rates and rate ratios of suicide in individuals from an ethnic minority background globally.

**Added value of this study**

With evidence from 128 studies and 31 countries, we estimated the absolute rate of suicide in ethnic minority groups to be 12·1 per 100,000 (95% CIs 8·4–17·6), with study estimates ranging between 1·2 and 139.7 per 100,000. There was weak statistical evidence of an increased risk of suicide amongst individuals from an ethnic minority background (RR 1·3 95% CIs 0·9–1·7), with substantial variation between study estimates (RR range 0·2–18.5). When examining suicide rates amongst subgroups, indigenous populations consistently reported high rates of suicide (23·2 per 100,000; 95% CIs 14·7–36·6) as well as higher risk of suicide when compared to the general population (RR 2·8 95% CIs 1·9–4·0).

**Implications of all the available evidence**

Despite the prevailing homogenous grouping of individuals from ethnic minority backgrounds in research and in the public discourse, our study provides evidence of how this practice is inadequate due to high levels of heterogeneity and inherent differences amongst groups. Clinicians and policymakers need to carefully consider this evidence when supporting individuals from ethnic minority backgrounds and creating publicly funded government resources.

Alt-text: Unlabelled box

## Introduction

Over 700,000 people die annually by suicide globally.^1^ Suicide is a universal concept but has different meanings worldwide.^2^^,^^3^ Suicide rates are likely to vary between culturally distinct populations within nations. Previous evidence suggests that for certain ethnic minority groups, self-harm (regardless of suicidal intent) is more likely than majority groups.^4^^,^^5^ Individuals from ethnic minority groups are more likely to experience language barriers, acculturative stress, and discrimination which influence suicide risk.^6^, ^7^, ^8^ However, research regarding suicide risk and ethnic minority status is mixed, with some evidence suggesting higher rates of suicide in ethnic minority groups, whilst other studies point to an opposite effect.^8^ This may be explained by the differing meaning of suicide in different cultural groups which confers different risk of suicide, and/or be due to the varying underlying mortality rate of suicide by country. In some settings suicide is seen as an acceptable response to social stressors, especially in south Asia,^2^^,^^9^ whereas in other contexts religious beliefs might make suicide less likely. Another possible explanation may be due to the way in which ethnic minority groups are defined. Some studies define ethnic minority status based on presumed/self-reported ethnicity^5^ while others determine by migrant status^8^ or indigenous.^10^^,^^11^ The presumption of ethnicity is problematic as it has been shown to misclassify individuals and is often based on skin colour.^12^

Whilst indigenous groups are often considered as separate to ethnic minority groups, the definition of an ethnic minority group includes similar concepts as indigenous groups, and therefore technically are an ethnic minority group (Box 1).^13^

In some countries, these groups are often described together (e.g., Black, Indigenous, and People of Colour in the United States). There are clearly differences between ethnic minority groups and those who are migrants, or indigenous peoples in terms of certain rights and histories. Yet, they all share common challenges and many of the risk factors which elevate suicide risk (e.g., discrimination, deprivation, social fragmentation).^8^^,^^14^^,^^15^ Whilst there is general recognition that these groups are different, non-majority ethnic individuals are often grouped and considered together. Indigenous status is sometimes not delineated^16^ and is even less likely to be recorded in mortality data from low-and-middle-income-countries (LMICs)^11^ where close to 80% of indigenous people live.^13^ As most indigenous groups tend to be minorities in countries, they may be classified as individuals from an ethnic minority background without specification of indigenous status. Understanding whether the rate of suicide is higher in these potentially marginalised groups than in the majority ethnic population is important to guide suicide prevention. It is also key to understanding whether there are important differences in rates by individual/group factors (e.g., sex, age, and migratory status), as well as contextual characteristics (e.g., continent where these ethnic minority groups live).^10^ The importance of gaining a better understanding of the rate and risk of suicide in ethnic minority groups has been brought into the spotlight during the COVID-19 pandemic. Whilst rates of suicide have appeared to have dropped in many countries around the world,^17^ the rate of suicide has increased in marginalised communities.^18^^,^^19^ This is against a backdrop of the recognition that suicide deaths are less likely to classified as suicide deaths in ethnic minority groups.^20^

To our knowledge, there has been no previous systematic review which has aimed to comprehensively identify and synthesise original research to estimate the absolute rates and rate ratios (RR) of suicide in individuals from an ethnic minority background. We also aimed to explore the heterogeneity by certain individual (i.e. sex), group (i.e. migrant status and ancestral origin), and contextual (i.e. continent of residence) factors.

## Methods

This review was conducted and reported in accordance with established systematic review guidance: Preferred Reporting Items for Systematic Reviews and Meta-Analyses (PRISMA). An a priori protocol was registered in the international prospective register of systematic reviews (PROSPERO): registration number CRD42020197940.

### Search strategy and selection criteria

We searched three electronic databases (Medline, Embase, and PsycInfo) for studies published from January 1, 2000 to July 3, 2020, reporting absolute or relative suicide rates amongst ethnic minority groups following a comprehensive search strategy (Supplementary File 1). References of included studies were screened as well as previously identified systematic reviews relevant to the subject area.^4^, ^5^, ^6^, ^7^, ^8^^,^^10^^,^^11^^,^^21^, ^22^, ^23^, ^24^, ^25^, ^26^, ^27^, ^28^, ^29^, ^30^, ^31^ Full-text articles of non-English papers were assessed after relevant translations with a native speaker and/or Google Translate. See Box 2 for further full-text eligibility criteria and Supplementary File 2 for exclusion criteria.

### Data extraction

Pilot screening of 20 randomly selected articles from the search results was conducted amongst reviewers to ensure consistency. Afterwards, 3 pairs of reviewers independently screened titles, abstracts, and full texts using the Rayyan systematic review website.^28^ Data extraction was conducted by two reviewers independently based on a pre-piloted data extraction sheet (Supplementary File 3). Where there were discrepancies amongst reviewers, at all stages of the screening process, a third author (MIT/DK) resolved discrepancies. There was generally a high level of agreement amongst reviewers at each stage of the screening process (title/abstract 7·1%, 691/9800; full-text 1·3%, 12/916).

### Quality assessment

Assessment of methodological quality was conducted alongside data extraction. Studies were independently appraised by pairs of reviewers using an adapted version of the Newcastle Ottawa Scale.^32^ This scale has been used in previous meta-analysis studies,^33^^,^^34^ and does not provide an overall study score, nor can it be used to compare between study designs. It can only be used to compare between studies of a similar study design. The Newcastle Ottawa Scale is designed for case-control and cohort studies; the wording of the scale was altered to be relevant to the exposure and outcomes of interest. We used previously adapted versions of the scale for case series and cross-sectional studies.^33^^,^^35^ Three domains were considered for quality assessment: (a) selection of study groups, (b) comparability of the groups, (c) ascertainment of the exposure/outcome. As the scale is not comparable between study designs we adopted a similar approach to one we have used previously where we identified studies which were least likely to be affected by bias in terms of selection of suicide deaths (outcome) and assessment of ethnicity (exposure).^33^ This assessment criteria was decided prior to data extraction by authors and further details provided in Supplementary File 4. Disagreements regarding methodological quality of included studies were resolved by a third author (MIT/DK).

### Data analysis

In research, policy, and in the public discourse, individuals from ethnic minority backgrounds are referred to and considered as a homogeneous population and often compared to the majority population.^36^ Despite inherent historical, social, and cultural differences, most published research^36^ and government documentation^37^ continue to treat individuals from an ethnic minority background as one group. Because of this wider context we decided a priori to consider them as a single group in our main analysis. We conducted a random effects meta-analysis to pool the estimates of the absolute rates and rate ratios of suicide in individuals from ethnic minority backgrounds.^38^ However, recognising and considering the differences amongst ethnic minority groups, further analysis was conducted with subgroups to attempt to differentiate, using the available data, the distinct backgrounds as described below.

The primary analysis was conducted on studies of reasonable quality (i.e., moderate-high), and a secondary analysis was conducted including studies of low quality. A further post-hoc sensitivity analysis was conducted including only studies rated as high quality for overall absolute rates and RRs, and sex. Data were synthesised if there were a minimum of five estimates to pool (pre-specified in our protocol). In the absence of this, forest plots of the estimates without a pooled estimate were provided along with narrative synthesis. Where a study only provided an absolute rate of suicide for the minority ethnic group, we calculated (where possible) standardised mortality ratios which we have used as an approximate to RRs using the latest World Health Organisation official data (crude rates) for the corresponding country, when data were within a decade of the mid-point estimates^39^ and used this to represent the majority ethnic suicide rate. As an example, one study reported an absolute rate of 15.1 per 100,000 but no RR^40^ From the WHO data, the crude rate for that country in the midpoint of the study was 24.1 per 100,000 (95% CIs 22.8 to 25.0). Thus, the approximate RR was 15.1 / 24.1 = 0.63. We estimated the standard error of the approximate RR using standard errors of both these crude rates, which was 0.06 on the log scale. (See Lash et al. equation 18-4 for the formula used to estimate the log standard error.)^41^

Some studies reported multiple estimates, for example representing rates or RR in several different ethnic groups. In order to have a single estimate in each study for the overall pooled estimates, we first aggregated the estimates for the minority groups using a fixed-effects meta-analysis (which assumed a common rate in the minority groups under observation in each study). This was done for every study that had more than one estimate. These aggregated estimates (and their standard errors) were then entered into the main analyses (see below). Similar aggregations were done for subgroup analyses where we examined migratory status and ancestral origin.

We conducted a pre-specified subgroup analysis by sex (male/female), continent of residence, and migratory status (Migrant, Non-migrant). Given the diverse ethnic minority groups, we pre-specified a subgroup analysis which aimed to disaggregate ethnic minority groups into slightly more nuanced subgroups. For this analysis, we categorised study estimates into broad categories based on the continent individuals were reported to originate from. For example, if a study reported on suicide rates in individuals from an Italian background, these estimates were categorised as having ancestral origins in Europe. Not all studies, however, were grouped by continent. Estimates related to indigenous people were kept separate, as were estimates which were based on specific reported ethnic groups (e.g., Black, White, or Hispanic). Grouping by continent of origin is problematic due to individuals’ differences across continents, and because someone from Asia, for example, who migrated recently is likely to be distinct from a native who has historical Asian origins (sometimes many generations in the past). However, this is a step closer to a more nuanced understanding than how ethnicity is typically treated in the literature. We had pre-specified two additional analyses by method of suicide and ethnic density, but data were not consistently reported, nor enough variability presented to allow for a meaningful analysis. We present pooled estimates (overall, by subgroup, and sensitivity analysis) for absolute rates and RRs. We assessed the possibility of non-reporting bias through visual inspection (i.e. funnel plot) of all the studies contributing to the overall absolute and relative rate pooled estimates, and formally test for plot asymmetry using an Egger test. We conducted a limited number of additional post-hoc analysis to further investigate sources of heterogeneity and explore the robustness of our findings. The studies included provided data from a wide time period, and some studies included data for a restricted population (e.g. only young people) or specific methods of suicide. To explore whether the time period (split into 5-year bands) the study was published, and the age of the included participants explained any heterogeneity observed, we conducted three additional post-hoc analysis. This was restricted to our primary overall pooled estimates.

Because we pre-pooled estimates from studies prior to our primary analysis, our second post-hoc sensitivity analysis examined differences in results when the data were instead analysed as a multi-level meta-analysis. The approach respects the nesting estimates within studies (a two-level structure). Failure to account for this structure could result in confidence intervals that are too narrow, especially where the data were pre-pooled using fixed effects methods. As such, we repeated our primary and secondary analyses on rate and rate ratios using a multi-level meta-analysis with a random intercept for study.^42^ All analyses were undertaken in Stata 16.1 except the multi-level meta-analysis which was undertaken in R 4.1.1.

### Role of the funding source

The funder had no role in the study design, data collection, data analysis, data interpretation, or the writing of the report. All authors had full access to all the data in the study and had final responsibility for the decision to submit for publication.

## Results

**Figure 1:**
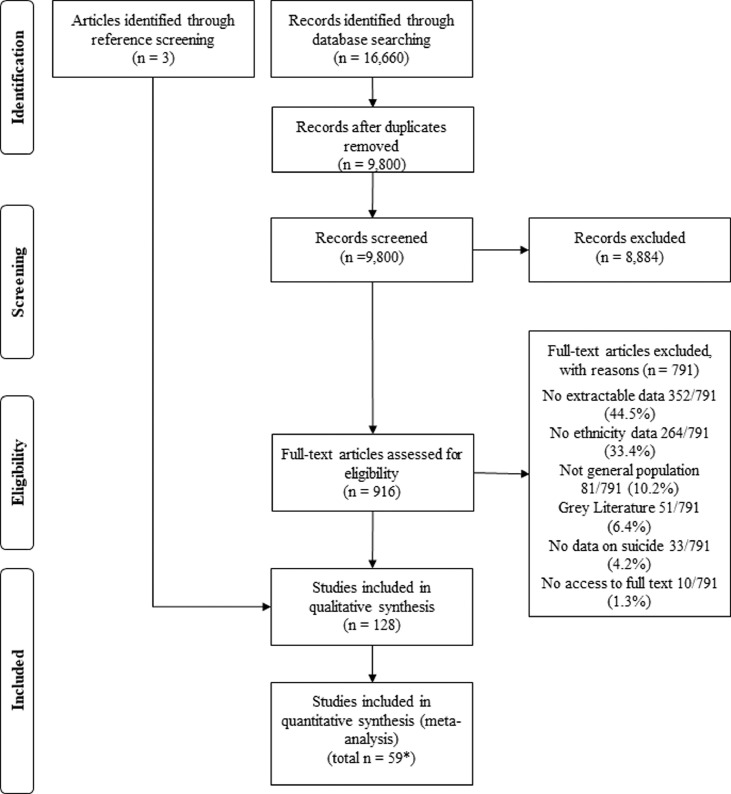
PRISMA Flow diagram of study selection (42 studies included in the absolute rate synthesis and 51 in the relative rate meta-analysis).

Of 9800 abstracts screened, 919 full texts were assessed for eligibility criteria and 128 met inclusion criteria, with 6,026,103 suicide deaths in individuals from ethnic minority groups living in 23 high-income countries representing 87·5% (112/128) of included studies, and eight LMICs representing 12·5% (16/128) studies (see Table 1, Figure 1, Supplementary File 5). Six non-English articles were included. Most studies had low-quality assessments (69/128, 53·9%), whilst 24 (18·8%) were high-quality, and the remaining 35 (27·3%) moderate quality. Six (5%) of studied reported on a specific method of suicide. Twenty-five (19·5%) studies reported on indigenous populations and 41 (32·0%) on migrants. Roughly half of the studies measured ethnicity by self-report or census linkage (66/128, 51·6%). A list of excluded studies with reasons are provided in Supplementary File 6.

**Table 1:** Characteristics of included studies.

Author (Year published) Country	Year (data extracted)	No. participants (%male/age range)	Study Design	Method of Suicide*	No. of ethnic minority groups	How ethnic group was defined	Quality assessment
Pavlovic (2001) Australia, Croatia, Slovenia^43^	Australia: 1988–1997, Croatia and Slovenia:1985–1994	28,788 (NA/ all ages)	Case series	All	4	Proxy: Migrant status	Low
Malenfant (2004) Canada^44^	1995–1997 (average)	3863 (79·1%/all ages)	Case series	All	2	Proxy: Migrant status	Low
Karmali (2005) Canada^45^	1999–2002 data grouped	NA (NA/ all ages)	Cohort	‘Traumatic’ suicide	2	Self-report/census linked	High
Yun (2016) USA^46^	2000–2014	26,857 (NA/ all ages)	Case series	All	3	Coroner/death records	Moderate
Wissow (2001)** USA^47^	1990–1993	23 (NA/ all ages)	Case series	All	1	Self-report/census linked	High
Wainiqolo (2012) Fiji^48^	2005–2006	73 (63·9%/all ages)	Case series	All	2	Self-report/census linked	Low
Voracek (2009)** Austria^49^	1970–2006	1439 (NA/ all ages)	Case series	All	21	Proxy: Migrant status	Low
Telisinghe (2014) Brunei^50^	1999–2010	124 (81%/ all ages)	Case series	All	3	Coroner/death records	Moderate
Spicer (2000) USA^51^	1990–1996 (varied by state)	7104 (NA/ all ages)	Case series	All	4	Self-report/census linked	High
Singh (2001) USA^52^	1979–1989	0 (NA/all ages)	Cohort	All	2	Proxy: Migrant status	Low
Singh (2004)** USA^53^	1986–1994	0 (NA/all ages)	Case series	All	6	Self-report/census linked	High
Saunders (2019) Canada^54^	2003–2012	6484 (74·8%/all ages)	Cohort	All	2	Proxy: Migrant status	Low
Rockett (2010) USA^20^	2003–2005	92,218 (79·7%/all ages)	Case series	All	3	Coroner/death records	Moderate
Pridmore (2009) Australia^55^	2001–2006	256 (NA/all ages)	Case series	All	2	Coroner/death records	Moderate
Orellana (2016) Brazil^56^	2009–2011	600 (78%/all ages)	Case series	All	2	Coroner/death records	Moderate
Bando (2012) Brazil^57^	1998–2008	4748 (NA/all ages)	Case series	All	4	Coroner/death records	Moderate
Norredam (2013) Denmark^58^	1993–1999	0 (NA/all ages)	Cohort	All	3	Proxy: Migrant status	Low
Merrill (2019) USA^59^	2011–2015	203,101 (78·9%/all ages)	Case series	All	3	Coroner/death records	Moderate
Kuroki (2018) USA^60^	2010	35,376 (78·9%/all ages)	Case series	All	7	Coroner/death records	Moderate
Kposowa (2000) USA^61^	1979–1989	545 (79%/ all ages)	Cohort	All	3	Self-report/census linked	High
Kõlves (2015) Australia^62^	2011	5752 (76·9%/ all ages)	Case series	All	2	Coroner/death records	Moderate
Kanamori (2020) Sweden^63^	2011–2016	5423 (70·2%/ all ages)	Cohort	All	5	Proxy: Migrant status	Low
Jung (2009) Romania^64^	2005–2006	273 (81%/ all ages)	Case series	All	3	Self-report/census linked	High
Jiang (2016) USA^65^	2006–2013	913 (79%/all ages)	Case series	All	4	No description provided	Low
Homer (2005) USA^66^	1988–1998	182 (70·3%/all ages)	Case series	Carbon monoxide	3	Coroner/death records	Moderate
Harrop (2007) Canada^67^	1985–1994	180 (NA/young people 0–19)	Case series	All	2	Self-report/census linked	High
Hanlon (2019) USA^68^	2005–2013	43,555 (87%/all ages)	Case series	Firearm	3	Coroner/death records	Moderate
Goosen (2011) Netherlands^69^	2002–2007	9020 (68·3%/ all ages)	Cohort	All	2	Proxy: Migrant status	Low
Garlow (2007) USA^70^	1989–2003	1257 (NA/all ages)	Case series	All	2	Coroner/death records	Moderate
Elo (2014) USA^71^	2007	0 (NA/Below the age of 75)	Case series	All	1	Coroner/death records	Moderate
Di Thiene (2015) Sweden^72^	2005–2010	4358 (71·7%/all ages)	Cohort	All	4	Proxy: Migrant status	Low
Deckert (2015) Germany^73^	1990–1999	0 (NA/all ages)	Cohort	All	1	Proxy: Migrant status	Low
De Leo (2011) Australia^74^	1994–2007	7672 (NA/all ages)	Case series	All	2	Coroner/death records	Moderate
Day (2009)** USA^75^	1999–2003	204 (77·9%/all ages)	Case series	All	1	Coroner/death records	Moderate
Brennecke (2020) Germany^76^	2000–2017	206,056 (74%/all ages)	Case series	All	10	Proxy: Migrant status	Low
Wong (2014) USA^77^	1999–2009	4071 (NA/10–14 and 15–19)	Case series	All	2	Self-report/census linked	High
Garlow (2005) USA^78^	1994–2002	735 (79·9%/all ages)	Case series	All	2	Self-report/census linked	High
Kõlves (2015) Australia^79^	1991–2009	NA (NA/all ages)	Case series	All	2	Proxy: Migrant status	Low
Burrows (2006) South Africa^80^	2001–2003	4946 (81·8%/all ages)	Case series	All	4	Coroner/death records	Low
Burrows (2013) Canada^81^	2004–2007	3395 (77·0%/all ages)	Case series	All	2	Proxy: Other-home language	Low
Bauwelinck (2017) Belgium^82^	2001–2011	11,522 (72·9%/18–64)	Case series	All	7	Proxy: Migrant status	Low
Stockard (2002) USA^83^	1995	209 (51·1/all ages)	Case series	All	2	No description provided	Low
Shah (2009) England and Wales^84^	2001–2005	0 (NA/older people 65+)	Case series	All	18	Proxy: Migrant status	Low
Westman (2006) Sweden^85^	1994–1999	4,459,806 (50·7%/25–64)	Cohort	All	6	Proxy: Migrant status	Low
Liu (2011) Taiwan^86^	1979–1981	117 (61·5%/all ages)	Case series	All	3	Coroner/death records	Low
Cwik (2016)** USA^87^	2007–2012	0 (NA/ all ages)	Case series	All	1	Self-report/census linked	High
Dunlavy (2019) Sweden^88^	1993–2008	4989 (70·1%/25–64)	Cohort	All	3	Proxy: Migrant status	Low
Wen (2004) Taiwan^89^	1998–2000	128 (73·4%/20–64)	Case series	All	1	Coroner/death records	Moderate
Singh (2006) USA^90^	1999–2001	0 (NA/ all ages)	Case series	All	7	Self-report/census linked	High
Tuck (2011) England and Wales^91^	1993–2003	978 (NA/all ages)	Case series	Burning	2	Proxy: use name to provide ethnicity	Low
Abdalla (2013) Republic of Ireland^92^	2008	0 (NA/all ages)	Cross-sectional	All	1	Self-report/census linked	Low
Loh (2007) Singapore^93^	2002	366 (60%/all ages)	Case series	All	4	Coroner/death records	Moderate
Herman (2016) Fiji^94^	2005–2006	90 (NA/young people: 15–24)	Case series	All	2	Self-report/census linked	High
Soole (2014) Australia^95^	2004–2012	141 (67·4%/ young people: 10–17)	Case control	All	4	Coroner/death records	Low
Ougrin (2011)** England^96^	2005–2007	0 (NA%/all ages)	Case series	All	1	Proxy: Migrant status	Low
Mittendorfer-Rutz (2019) Sweden^97^	2017	94 (NA/ young people: 10–21)	Case series	All	2	Proxy: Migrant status	Low
Hjern (2002) Sweden^98^	1990–1998	0 (NA/all ages)	Cohort	All	20	Proxy: Migrant status	Low
Ide (2012) Australia^99^	Data for most 2004–2006, females from north Europe, central and south America, 1999–2003	0 (NA/all ages)	Case series	All	16	Proxy: Migrant status	Low
Sanford (2006)** USA^100^	2004	999 (77%/all ages)	Case series	All	2	No description provided	Low
Gilmour (2019) Japan^101^	2012–2016	0 (NA/all ages)	Case series	All	4	Proxy: Migrant status	Low
Iribarren (2000) USA^102^	1979–1993	319 (68·3%/all ages)	Cohort	All	4	Self-report/census linked	High
Hoffmann (2020) USA^103^	2007–2016	20,982 (NA/ young people: 5–19)	Case series	All	4	Coroner/death records	Moderate
Khan (2018) USA^104^	2012–2015	20,288 (NA/ young people: 10–24)	Case series	All	3	Coroner/death records	Moderate
Hastings (2015) USA^105^	2003–2011	0 (100%/ 25 and older)	Case series	All	6	Coroner/death records	Moderate
Webb (2015) Denmark^106^	1971–2002	1414 (80·3%/ follow up from adolescence to middle age)	Cohort	All	2	Proxy: Migrant status	Low
Ueda (2019) Japan^107^	2010–2014	136,582 (69·7%/all ages)	Case series	All	9	Proxy: Migrant status	Low
Rockett (2012) USA^108^	2000–2009	0 (NA/ all ages)	Case series	All	4	Coroner/death records	Moderate
DeMello (2020) USA^109^	2014	447 (79·2%/ all ages)	Case series	All	4	Self-report/census linked	High
Holck (2013) USA^110^	2004–2006	89,147 (79·1%/15–54)	Case series	All	2	Coroner/death records	Moderate
Krivo (2018) USA^111^	2008–2010	77,467 (NA/all ages)	Case series	All	2	Coroner/death records	Moderate
Lazzarini (2017)** Brazil^112^	2003–2013	119 (71·4%/all ages)	Cohort	All	1	Self-report/census linked	High
Pathak (2018) USA^113^	2014	1831 (100%/all ages)	Case series	All	2	Coroner/death records	Moderate
Day (2003) USA^114^	1979–1998	0 (NA/all ages)	Case series	All	1	Coroner/death records	Low
Bridge (2015) USA^115^	2008–2012	155 (77%/ young people: 5–11)	Case series	All	5	No description provided	Low
Sonderman (2014) USA^116^	2002–2011	57 (61·4%/40 to 79)	Cohort	All	2	Self-report/census linked	High
Puzo (2018) Norway^117^	1992–2012	11,409 (72·5%/ all ages)	Case series	All	5	Proxy: Migrant status	Low
Silviken (2009) Finland^118^	1970–1998	89 (78·7%/all ages)	Case series	All	1	Self-report/census linked	Low
Price (2019)** USA^119^	2001–2017	303 (73·9%/ young people: 13–19)	Case series	All	1	No description provided	Low
Pacot (2018)** French Guyana^120^	2008–2015	24 (58·3%/all ages)	Case series	All	1	No description provided	Low
Fairthorne (2016) Australia^121^	1983–2011	0(0%/all age groups of maternal age)	Cohort	All	2	Self-report/census linked	Low
Pollock (2018) Canada^122^	2014	6058 (NA/all ages)	Case series	All	6	No description provided	Low
Stefanac (2019) Australia^123^	2004–2014	3709 (74·1%/ young people: 10–24)	Case series	All	2	Coroner/death records	Moderate
Shah (2011) England and Wales^124^	2001–2005	0 (NA/all ages)	Case series	All	11	Proxy: Migrant status	Low
Silviken (2006) Norway^125^	1970–1998	89 (78·7%/all ages)	Case series	All	1	Self-report/census linked	Low
Amin (2019) Sweden^126^	2005–2013	9144 (NA/16–64)	Cohort	All	2	Proxy: Migrant status	Low
Hollander (2019) Sweden^127^	1970–2015	3747 (NA/16–43 year olds)	Cohort	All	3	Proxy: Migrant status	Low
Martin (2010) USA^128^	2004–2007	4218 (77%/all ages)	Case series	All	6	Coroner/death records	Low
Shoaf (2004) USA^129^	1991–1993	2525 (78%/all ages)	Case series	All	2	Coroner/death records	Low
Termorshuizen (2015) Netherlands^130^	2000–2011	3041 (NA/all ages)	Cohort	All	8	Proxy: Migrant status	Low
Werenko (2000) USA^131^	1990–1994	184 (85%/ young people: 0–20)	Case series	All	3	Coroner/death records	Moderate
Orellana (2019)** Brazil^132^	2007–2011	64 (80%/all ages)	Case series	All	1	Self-report/census linked	Low
Yau (2018) USA^133^	2013	2810 (NA/all ages)	Case series	Asphyxiation	5	Coroner/death records	Low
Ali (2012) Malaysia^134^	2009	328 (74%/all ages)	Case series	All	6	Coroner/death records	Low
Kua (2003) Singapore^135^	1991–2000	712 (56%/older people: 65 plus)	Case series	All	3	No description provided	Low
Värnik (2005) Estonia^136^	1991–1998	0 (NA/all ages)	Case series	All	2	Proxy: Migrant status	Low
Music (2014) Bosnia and Herzegovina^137^	2006 (for suicide rate numbers); 1990	190 (64%/all ages)	Case series	All	3	Proxy: Migrant status	Low
Measey (2006) Australia^138^	2001–2002	577 (NA/all ages)	Case series	All	2	Coroner/death records	Low
Herne (2014) USA^139^	1999–2009	4541 (77·9%/ all ages)	Case series	All	2	Self-report/census linked	High
EchoHawk (2006)** USA^140^	1994–1996	0 (NA/ young people: 15–24)	Case series	All	2	Self-report/census linked	High
Pollock (2016) Canada^141^	1993–2009	745 (84·8%/ all ages)	Case series	All	3	Proxy: region of residency	Low
Tian (2019) USA^142^	2003–2014	103,796 (78·6%/all ages)	Case series	All	3	Coroner/death records	Moderate
Sumarokov (2014) Russia^143^	2002–2012	252 (93·8%/ all ages)	Case series	All	2	Self-report/census linked	High
Bjerregaard (2015)** Greenland^144^	1970–2011	1678 (NA/ all ages)	Case series	All	1	Proxy: place of birth	Low
Maynard (2012) England and Wales, national^145^	1999–2003	20,739 (77·4%/all ages)	Case series	All	6	Proxy: Death records, country of birth	Low
Ruch (2019) USA^146^	2007–2016	3163 (65·8%/ young people: 10–19)	Case series	All	4	Coroner/death records	Moderate
Soole (2014) Australia^95^	2000–2010	45 (55·6%/ young people: 10–14)	Case series	All	2	Coroner/death records	Moderate
Rhoades (2003)** USA^147^	1994–1996	753 (80·2%/ all ages)	Case series	All	1	Self-report/census linked	High
Mullany (2009) USA^148^	2000–2006	0 (NA/ all ages)	Case series	All	3	Self-report/census linked	High
Styka (2010) USA^149^	2005–2006	516 (78·5%/ 18–64)	Case series	All	3	Coroner/death records	Moderate
Arya (2019) India^150^	2014–2015	265,289 (NA/ all ages)	Case series	All	4	Coroner/death records	Moderate
Vieweg (2005) USA^151^	2003	18 (NA/ young people: 10–17)	Case series	All	2	Coroner/death records	Moderate
Heninger (2008) USA^152^	2000–2004	21 (66·7%/ young people: 10–19)	Case series	All	2	Coroner/death records	Moderate
Matzopoulos (2015)** South Africa^153^	2009	6471 (82·2%/all ages)	Case series	All	4	Coroner/death records	Low
Saunders (2017) Canada^154^	1996–2012	0 (NA/young people: 10–25)	Cohort	All	2	Proxy: Migrant status	Low
Nestadt (2017) USA^155^	2003–2015	6196 (79·6%/all ages)	Case series	All	4	Coroner/death records	Low
Matthay (2017) USA^156^	2013	4012 (NA/all ages)	Case series	All	6	Coroner/death records	Low
Øien-Ødegaard (2019) Norway^157^	2007–2014	4341 (NA/all ages)	Cohort	All	2	Proxy: Migrant status	Low
Kerr (2003) USA^158^	1996–1998	576 (79·9%/ young people: 15–19)	Case series	All	3	Coroner/death records	Low
Pear (2018) USA^159^	2011–2015	1575 (88%/all ages)	Case series	Firearm	4	Coroner/death records	Moderate
Ferreira (2011) Brazil^160^	2004–2006	127 (29%/all ages)	Case series	All	2	Self-report/census linked	High
Garssen (2007) Netherlands^161^	2005	13,214 (70%/15–54)	Case series	All	4	Proxy: Migrant status	Low
Kobori (2017)** Japan^40^	2010	324 (NA/ all ages)	Case series	All	1	Proxy: Migrant status	Low
Koppenaal (2003) Netherlands^162^	1999	20 (90%/all ages)	Case series	All	1	Proxy: Migrant status	Low
Razum (2004) Germany^163^	1992–1997 for absolute rates, 1980–1997 relative rates	0 (NA/ 10–64 year olds)	Case series	All	1	Proxy: Migrant status	Low
Souza (2019) Brazil^164^	2010–2014	584 (58%/ young people: 10–14)	Case series	All	2	Coroner/death records	Moderate
Bhupinder (2010)** Malaysia^165^	2009	48 (70·8%/all ages)	Case series	All	3	Coroner/death records	Low
Hassler (2005) Sweden^166^	1961–2000	0 (NA/all ages)	Cohort	All	3	Self-report/census linked	High
Soininen (2008) Finland^167^	1979–2005	0 (NA/all ages)	Cohort	All	3	Self-report/census linked	High

*Traumatic suicide defined as methods that cause the person to sustain severe multi system injury. Studies which include all methods of suicide deaths are those that have included suicide deaths regardless of methods - the distribution of types of methods of suicide included in each study may, however, vary greatly. Some studies (as indicated in the table) only included specific methods of suicide.

^⁎⁎^ Only absolute rates were provided for these studies. In order to include these studies in the synthesis of relative rates, standardised mortality ratios were calculated using the WHO crude rate.

**Box 1:** Definitions of ethnic minority groups and indigenous groups.

Term	Definition
Ethnic Minority Groups	A group of individuals who share common characteristics (e.g., language, tribe) and sense of identity, but are a group who are in a non-dominant position in a given country.^13^
Indigenous Groups	Those which, having a historical continuity with pre-invasion and pre-colonial societies that developed on their territories, consider themselves distinct from other sectors of the societies now prevailing on those territories, or parts of them; they form at present non-dominant sectors of society and are determined to preserve, develop and transmit to future generations their ancestral territories, and their ethnic identity, as the basis of their continued existence as peoples, in accordance with their own cultural patterns, social institutions, and legal system”.^13^

**Box 2:** Inclusion and exclusion criteria.

Inclusion criteria	
Population	Ethnic minority groups, including migrant groups and indigenous peoples, in general populations. Studies reporting on specific clinical groups or other subgroups were not included (e.g., army veterans).
Exposure	Death by suicide amongst ethnic minority groups.
Comparator	Suicide rates in majority populations (if reported).
Study design	All epidemiological study designs providing data on suicide rates, or data from which rates can be derived
Setting	General population (i.e., non-clinical populations or specific subgroups).
**Exclusion criteria**	
Studies reporting on:	Clinical or specific population groups, non-fatal self-harm, not peer reviewed due to high risk of bias, and where full-texts were not available. Studies which reached full-text stage but did not provide data which allowed for pooling of estimates were excluded (Supplementary File 2).

### Absolute suicide rates

#### Overall

**Figure 2:**
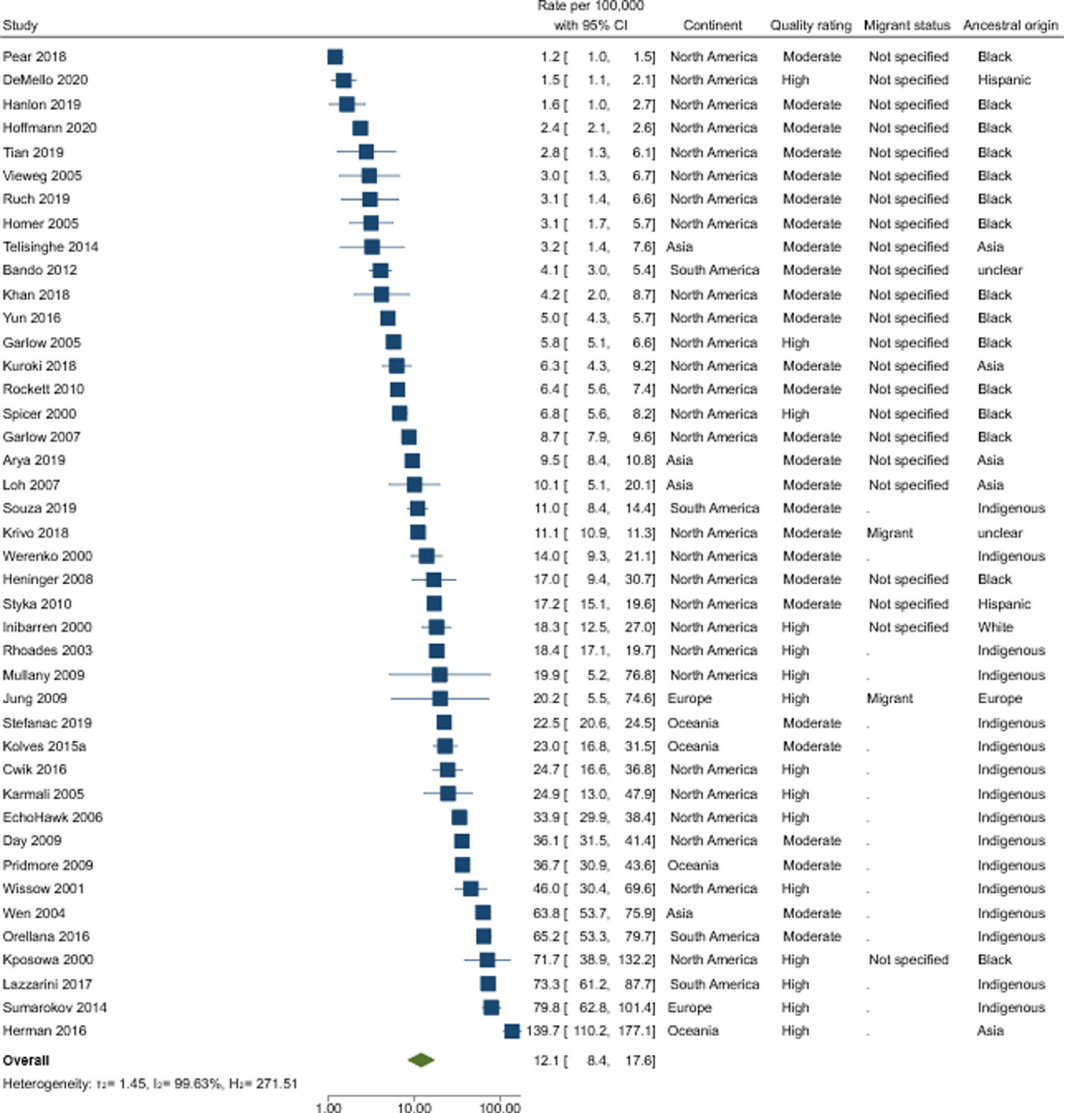
Forest plots reporting absolute rates (with 95% Confidence Intervals) per 100,00 amongst individuals from an ethnic minority background in *n* = 42 moderate-high quality studies.

A total of 42 studies, with moderate-high quality assessment, were included in our primary analysis, which produced a pooled rate of suicide of 12·1 per 100,000 (95% CIs 8·4–17·6) in individuals from ethnic minority backgrounds (Figure 2). There was evidence of a high degree of heterogeneity (*I^2^* = 100%) and broad rates range of 1·2–139·7 per 100,000. Whilst there was visual evidence that the included studies in this analysis tended to be larger in size (Supplementary file), there was no statistical evidence of plot asymmetry (*p* = 0.38).

*Subgroups:* Twenty of the 42 moderate-high quality studies reported on male, and 18 on female-specific suicide rates. Males had a suicide rate of 22·6 per 100,000 (95% CIs 13·5–37·9), and females had a suicide rate of 6·8 per 100,000 (95% CIs 3·6–12·7). There was a high degree of heterogeneity, with estimates ranging between 2·9 and 138·1 per 100,000 in males (*I^2^* = 99%), and 0·3–41·7 per 100,000 in females (*I^2^* = 98%).

Pooled point estimates for North American studies (*n* = 28) were 8·5 per 100,000 (95% CIs 5·6–12·9) and significant heterogeneity persisted (range 1·2–71·7 per 100,000; *I^2^* = 100%). There were insufficient studies to report on pooled absolute rates of suicide by continent amongst the other continents (Supplementary File 7). Estimates for suicide rates in Oceania (*n* = 4) were largely consistent with each other (range 22·5–36·7 per 100,000) with one notable exception^94^ (139·7 per 100,000 95%CIs 110·2–177·1). This study was the only one to record ethnicity based on self-report. There were only two moderate-high quality studies from Europe, and both reported consistent estimates (i.e., overlapping confidence intervals) ranging between 20·2^64^ and 79·8^143^ per 100,000. Out of the moderate-high quality studies from Asia, three^50^^,^^93^^,^^150^ reported consistent rates ranging between 3·2 and 10·1 per 100,000, with one exception^89^ which reported rates as high as 63·8 per 100,000 and compared to the other studies reported in indigenous people. Lastly, in South America only two of the absolute rate estimates were consistent with each other^56^^,^^112^ (range 65·2–73·3 per 100,000), with two studies reporting much lower rates:4·1 per 100,000,57 11·0 per 100,000.^164^ These two studies differed from the other studies as they reported exclusively on indigenous populations.

We examined absolute rates by migrant status and only provide pooled estimates for studies where the migratory status was unspecified (Table 2). Rates of not specified groups were 5·8 per 100,000 (95% CIs 4·0–8·5) with high levels of heterogeneity (range 1·2–71·7 per 100,0000; *I^2^* = 99%). There were insufficient studies to pool by migrant and non-migrant status. Supplementary File 7 provides the forest plots of absolute rates of migrant groups (*n* = 3) in Asia, Europe, and US. These rates varied (6·8–37·7 per 100,000).

**Table 2:** Absolute suicide rates per 100,000 in individuals from an ethnic minority background by continent, migrant status, and region of ancestral origin

	Moderate and high-quality studies			All studies		
	Rate (95% CIs)	I^2^	No. of studies/estimates	Rate (95% CIs)	I^2^	No. of studies/estimates
Overall	12·1 (8·4-17·6)	99·6%	42	12·4 (9·5-16·2)	99·8%	85
**Sex**						
Male	22·6 (13·5-37·9)	99·5%	20	20·6 (15·3-27·7)	99·6%	53
Female	6·8 (3·6-12·7)	98·2%	18	6·6 (4·9-8·9)	98·6%	51
**Continent**						
Africa	-	-	0	-	-	2
Asia	-	-	4	11·0 (4·7-25·7)	99·0%	8
Europe	-	-	2	15·4 (10·1-23·4)	99·4%	20
N. America	8·5 (5·6-12·9)	99·6%	28	8·6 (5·8-12·7)	99·8%	43
Oceania	-	-	4	33·3 (17·3-64·4)	98.2%	6
S. America	-	-	4	38·3 (12·0-122·7)	99·1%	6
Migrant Status*						
Migrant	-	-	2	11·5 (7·7-17·4)	99·8%	22
Non-migrant	-	-	0	-	-	3
Not specified	6·6 (4·2-10·3)	99·1%	29	6·5 (4·5-9·3)	99·2%	40
**Ancestral Origin**						
Asia	7·8 (2·9-20·6)	98·9%	9	9·1 (5·3-15·6)	98·3%	19
Black	4·6 (2·9-7·2)	99·8%	18	4·3 (3·0-6·3)	99·8%	27
Africa	-	-	0	-	-	3
Europe	-	-	2	25·3 (12·1-52·6)	98·2%	8
Hispanic	5·9 (2·5-14·2)	99·9%	9	4·0 (2·1-7·8)	99·9%	17
Latino	-	-	1		-	1
South America	-	-	0	-	-	1
North America	-	-	0	-	-	3
Indigenous	23·2 (14·7-36·6)	99·2%	19	23·4 (14·6-37·4)	99·6%	29
Middle East	-	-	0	-	-	-
Not clear	4·9 (3·5-8·1)	99·9%	13	7·2 (4·9-10·7)	99·9%	37

⁎ Indigenous groups are not included in this sub-group analysis.

When examining by ancestral origin, highest rates were estimated amongst indigenous (23·2 per 100,000; 95% CIs 14·7–36·6), and lowest pooled estimated in ethnically Black individuals (4·6 per 100,000; 95% CIs 2·9–7·2). There was still substantial heterogeneity (*I^2^* > 98%). There were insufficient studies to report ancestral origin pooled rates of individuals from Europe, Latino, and White individuals. These are summarised in Supplementary File 7, whilst the rates of suicide were low in ethnically Latino individuals^104^ (4·0 per 100,000), individuals from Europe^64^ (37·7 per 100,000, 95% CIs 32·4–43·7) or White individuals^102^ (28·1 per 100,000, 95% CIs 24·7–32·0) had similar rates.

*Secondary analysis:* We repeated the main analysis including all studies regardless of methodological quality (Table 2). A total of 85 studies produced a pooled rate of 12·4 per 100,000 (95% CIs 9·5–16·2) with high degree of heterogeneity *I^2^* = 100% (range 0·1–172·0 per 100,000). Consistent with main results, similar rates were reported amongst studies of any methodological quality for male and female-specific studies (males: 20·6 per 100,000;95% CIs 15·3–27·7, females 6·6 per 100,000; 95% CIs 4·9–8·9). We repeated all subgroup analyses including all studies regardless of methodological quality (Supplementary File 8).

In Supplementary File 9, we report absolute suicide rates in studies of high-quality only (*n* = 15). Most were North American (11/15) and based on indigenous populations (8/15). The pooled point estimate was consistent with the findings from the primary analysis with substantial heterogeneity (*I^2^* = 99%; range 1·5–139·7). When examining male and female-specific rates, these were higher than when including moderate quality studies: males 34·0 per 100,000; 95% CIs 15·4–74·8, females 8·2 per 100,000; 95% CIs 3·3–20·1. Substantial heterogeneity remained (*I^2^* > 98%). Substantial heterogeneity (*I^2^* > 98%) also persisted when stratified by when studies were published (5-year periods). The pooled rate of suicide in studies with no age restrictions were consistent with the primary analysis (11.6 per 100,000; 95% CIs 7.5–17.9) and with substantial heterogeneity (*I^2^* > 99%) between study estimates. Excluding studies which presented on specific methods of suicide provided a consistent rate estimate (13.8 per 100.00; 95% CIs 9.6–20.0) to that of the main analysis with substantial heterogeneity ((*I^2^* > 99%) between study estimates.

In Supplementary file 10, we report the results of the primary and the secondary analysis using a multi-level meta-analysis. These results are largely consistent with the results from the primary and secondary analyses. To illustrate, the overall rate in the moderate and high-quality studies was 10.8 per 100,000 (95% CIs 7.8 to 15.0). The *I^2^* was 65% at the study level (42 studies) and 35% at the study level (145 estimates). Similar results were observed for all other rates.

### Rate ratios

#### Overall

**Figure 3:**
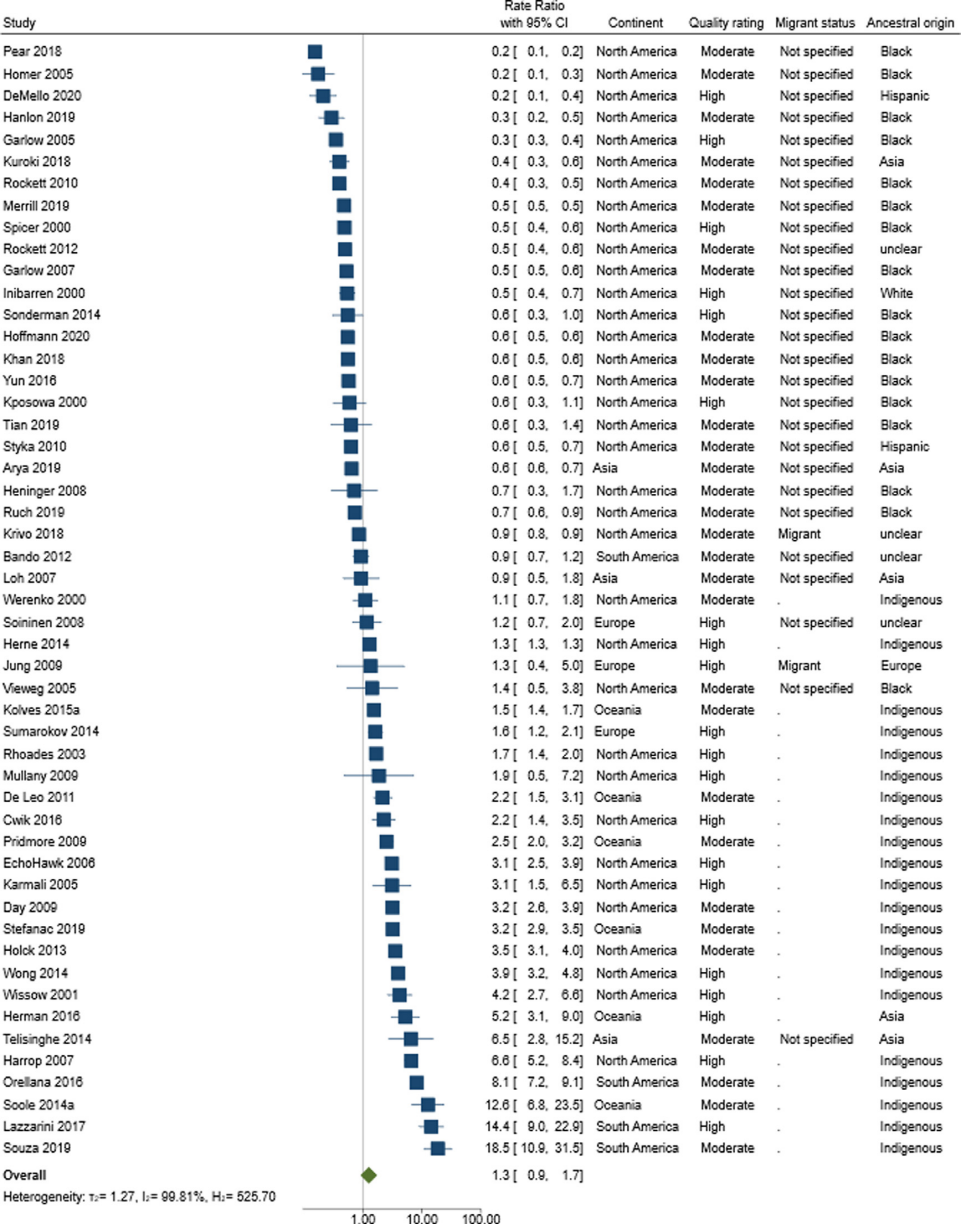
Forest plots reporting rate ratios (with 95% Confidence Intervals) amongst individuals from an ethnic minority background compared to majority ethnic population in *n* = 51 moderate-high quality studies.

A total of 51 studies of moderate-high quality rating provided RR of suicide in individuals from an ethnic minority background compared to the majority ethnic population. Overall, there was no statistical evidence that individuals from an ethnic minority background were any more likely to die by suicide (RR 1·3 95% CIs 0·9–1·7) (Figure 3). There was, however, evidence of substantial heterogeneity (*I^2^* = 100%), with a broad ranging RR: 0·2–18·5. Visual inspection of the funnel plots suggests that smaller studies which indicate a reduction in risk were less likely to be reported/published and therefore included in our review (Supplementary file 12). However, there was limited statistical evidence of plot asymmetry (*p* = 0.08).

*Subgroups:* A total of 27 studies reported on male-specific RRs, and 24 on female-specific RRs. There was no statistical evidence of an increased risk of suicide in both sexes (Table 3): males: RR 1·2, 95% CIs 0·8–2·0; females: RR 1·4, 95% CIs 0·8–2·4, and substantial heterogeneity persisted (*I^2^* > 99%). Pooled point estimates by continent found that individuals from ethnic minority backgrounds were more likely to die by suicide in Oceania (*n* = 6) (RR: 3·3 95% CIs 1·9–5·9), compared to limited evidence of elevated risk in North America (*n* = 35) (RR: 0·9 95% CIs 0·6–1·1) (Table 3). Within these two subgroups substantial heterogeneity persisted (Oceania: *I^2^* = 98%, RR range 1·5–12·6; North America: *I^2^* = 100%, RR range 0·2–6·6). Supplementary File 7 provides forest plots of these studies. Three^56^^,^^112^^,^^164^ of the four studies from South America reported a higher risk of suicide amongst individuals from an ethnic minority background, specifically indigenous people (8 to 19-fold increased risk). The one study which did not report an elevated risk was a study which reported suicide rates in a highly populated urban city Sao Paulo by ‘race’.^57^ European studies reported estimates ranging between a 16% and 62% increased risk.^64^^,^^143^^,^^167^ There was more variability between study estimates from Asia (*n* = 3), with two studies which were consistent with a reduction in risk,^93^^,^^150^ and one study reporting increased risk.^50^ This latter study included indigenous people.

**Table 3:** Rate ratios amongst individuals from an ethnic minority background compared to majority ethnic population by continent, migrant status, and region of ancestral origin

	Moderate and high-quality studies			All studies		
	Rate Ratios (95% CIs)	I^2^	No. of studies/estimates	Rate Ratios (95% CIs)	I^2^	No. of studies/estimates
Overall	1·3 (0·9-1·7)	99·8%	51	1·2 (0·9-1·5)	99·7%	97
**Sex**						
Male	1·2 (0·8-2·0)	99·7%	27	1·1 (0·9-1·5)	99·4%	68
Female	1·4 (0·8-2·4)	99·2%	25	1·2 (0·9-1·6)	98·4%	67
**Continent**						
Africa	-	-	0	-	-	2
Asia	-	-	3	1·3 (0·5-3·0)	98·2%	7
Europe	-	-	3	1·0 (0·8-1·3)	96·7%	23
N. America	0·9 (0·6-1·2)	99·8%	35	0·9 (0·6-1·1)	99·8%	50
Oceania	3·3 (1·9-5·9)	98·0%	6	3·1 (1·7-5·6)	98.0%	9
S. America	-	-	4	7·9 (3·0-21·0)	97·9%	6
Migrant Status*						
Migrant	-	-	3	1·0 (0·7-1·4)	99·4%	26
Non-migrant	-	-	0	1·3 (0·7-2·3)	95·0%	5
Not specified	0·6 (0·5-0·8)	98·8%	30	0·6 (0·5-0·8)	98·8%	46
**Ancestral Origin**						
Asia	0·8 (0·4-1·9)	97·9%	9	0·9 (0·5-1·6)	98·1%	19
Black	0·5 (0·4-0·5)	99·3%	21	0·5 (0·4-0·6)	99·4%	30
Africa	-	-	0	-	-	3
Europe	-	-	1	1·0 (0·7-1·5)	93·7%	9
Hispanic	0·5 (0·4-0·6)	98·7%	10	0·4 (0·4-0·6)	98·7%	18
Latino	-	-	1	-	-	1
South America	-	-	0	-	-	1
North America	-	-	0	-	-	2
Indigenous	2·8 (1·9-4·0)	99·2%	25	2·8 (2·0-4·0)	99·3%	35
Middle East	-	-	0	0·4 (0·3-0·5)	92·9%	6
Not clear	0·7 (0·4-1·1)	99·9%	16	0·8 (0·6-1·0)	99·9%	44

⁎ Indigenous groups are not included in this sub-group analysis.

When examining RR by migrant status, there were only sufficient data to calculate a pooled estimate for studies where migrant status was not specified (Table 3). These studies no evidence of either an increase or decrease in risk with substantial heterogeneity (*I^2^* = 98%, RR range 0·2–4·6). Supplementary File 7 provides forest plots of studies reporting on migrants (*n* = 3). With the exception of a study from the US,^111^ the other two studies from Europe^64^ and Asia^50^ report an increased suicide risk (RR range 2·5–13·6).

The final pre-specified subgroup analysis was by ancestral origin. Indigenous groups had increased risk of suicide: RR 2·8 (95% CIs 1·9–4·0) with significant heterogeneity observed (*I^2^* = 99%, RR range 0·4–18·5), whilst there was no statistical evidence of increased risk for any other ancestral origin groups (Table 3). One study reported RR by ancestral origin amongst individuals from Europe, which showed an increased risk of suicide (RR 2·5 95% CIs 2·0–3·2),^64^ whereas the single study reporting on young Latino individuals indicated a halving in risk^104^ (Supplementary File 7).

*Secondary analysis:* A total of 97 studies (regardless of study quality) reported on suicide RR amongst individuals from an ethnic minority background; the inclusion of lower quality studies provided consistent estimates to our main analysis (RR 1·2 95% CIs 0·9–1·5) with high degree of heterogeneity *I^2^* = 100 (RR range 0·2–21·9) (Table 3). Across these studies, risk of suicide for males and females were similar (males:1·1; 95% CIs 0·9–1·5, females:1·2; 95% CIs 0·9–1·6), and consistent with our main findings. We repeated all other subgroup analyses including all studies regardless of methodological quality in Supplementary File 8.

In Supplementary File 9, we report RR in high-quality studies (*n* = 20). Most were North American (15/20) and over half in indigenous populations (11/20). There was statistical evidence that individuals from an ethnic minority background were at an increased risk of suicide compared to majority ethnic groups: RR 1·6; 95% CIs 1·0–2·6. All the non-indigenous studies either indicated a reduction in risk or no risk, whereas indigenous studies were consistent with an elevated risk of suicide. There was still substantial heterogeneity *I^2^* = 99% (range 0·2–14·4). When examining male and female-specific RR, this increased risk remained, though the estimates were consistent with chance: males RR 1·5; 95% CIs 0·8–2·8 and females RR 1·4; 95% CIs 0·6–3·1 (*I^2^* = 99%). Subgroup analysis by year of study did not account for the heterogeneity observed (subgroup *I^2^* range 96–100%). The pooled RR in studies with no age restrictions were consistent with the primary analysis (RR 1.0 95% CIs 0.7–1.4) and with substantial heterogeneity (*I^2^* > 100%) between study estimates. The same consistent finding was observed when only studies including all methods of suicide were included (RR 1.4 95% CIs 1.0–1.19 *I^2^* > 99%), with some statistical evidence of an increased risk of suicide in ethnic minority groups.

In Supplementary file 11, we report sensitivity analyses using a multi-level meta-analyses. The results using this approach were similar to that reported for the primary and sub-group analyses. For example, the overall RR for the multi-level meta-analysis for moderate and high-quality studies was 1.2 (95% CIs 0.9 to 1.7) with heterogeneity largely attributable to the between study effects (I^2^ = 90% for studies and 10% for estimates). A similar finding was observed when all studies were considered (RR = 1.1, 95% CIs 0.9 to 1.4).

## Discussion

To the best of our knowledge, this is the first systematic review reporting suicide mortality rates amongst individuals from an ethnic minority background. Despite the prevailing homogenous grouping of individuals from ethnic minority backgrounds in research, policy, and the public discourse, our study provides evidence of how this practice is inappropriate due to high levels of heterogeneity, varying estimates, and inherent differences amongst groups. With evidence from 128 studies, and 6,026,103 suicide deaths across 31 countries, we estimated the absolute rate of suicide in ethnic minority groups to be 12·1 per 100,000 (95% CIs 8·4–17·6), with study estimates ranging between 1·2 and 139·7 per 100,000. There was no statistical evidence of an increased risk of suicide in moderate-high quality studies amongst individuals from an ethnic minority background (RR 1·3 95% CIs 0·9–1·7), with substantial variation (RR range 0·2 to 18·5). However, when examining suicide risk amongst high-quality studies (*n* = 20), there was statistical evidence for an increased risk of suicide amongst individuals from an ethnic minority background (RR:1·6; 95% CIs 1·0–2·6). In all our subgroup analyses substantial heterogeneity persisted (*I^2^* > 98%). This heterogeneity remained unexplained in our subgroup analysis by migratory status (where this was provided) or broad ancestral origin (which crudely maps to the ethnic minority groups reported elsewhere).

When examining suicide rates amongst subgroups, indigenous populations consistently reported high rates of suicide (23·2 per 100,000; 95% CIs 14·7–36·6) as well as higher suicide risk (RR 2·8 95% CIs 1·9–4·0). Most studies were based in high income countries (87·5%), pointing to a major gap in our understanding. Given that close to 80% of indigenous people live in LMICs,^13^ they host 80% of the world's refugee population^168^ and 80% of all suicide deaths occur in LMICs,^1^ this knowledge gap is of concern.

Previous studies indicate that individuals from ethnic minority backgrounds are less likely to have their death recorded as suicide than their majority counterparts.^20^ Research^169^ has pointed to the misclassification of individuals from ethnic minority backgrounds when the recording of ethnicity is based on observer classification (e.g., increased likelihood of misclassification of individuals from Black or mixed groups by healthcare workers^12^). In our review, studies which were high-quality rated were those which used secure records to identify ethnicity (e.g., census, official records (i.e., linked data where ethnicity is based on self-report), or self-report). This was to identify the studies which had accurately captured ethnicity. Less than a fifth of studies (24/128, 18·8%) were rated as high-quality, with half being indigenous studies which are all consistent with an elevated risk of suicide compared to their majority counterparts. Suggesting that the apparent reduction in risk in other ethnic minority groups might be a consequence of systemic barriers rather than a true risk reduction.

Previous research has indicated that migratory status of individuals from ethnic minority backgrounds changes risk profiles, with recent migrants being less likely to die by suicide compared to their majority counterparts, whereas descendants of these migrants (i.e., non-migrants) are at elevated risk of suicide.^72^ Most studies in non-indigenous populations did not specify migration status thereby making it impossible to disentangle mixed effects. Future work should report on migratory status and include how many generations removed descendants are from their migrant ancestor.

Our review was conducted in accordance with established systematic review guidance. Nonetheless, our study had limitations. First, over half of included studies had low-quality assessments (53·9%) which could indicate increased risk of bias. However, our primary analysis was conducted with moderate-high quality studies, reducing the risk. Second, we limited inclusion to manuscripts with English language titles or abstracts – if they did not have these they would not have appeared in our searches. This may have led to underreporting and exclusion of studies from non-English speaking countries. We attempted to overcome this by searching the references all included papers and previous reviews. Third, we limited our review to peer-reviewed articles, excluding grey literature which may mean our review may be subject to publication bias. Fourth, for several papers relative rates were either not presented or were not able to be calculated from presented data. To avoid exclusion of these studies which presented only absolute rates from our RR synthesis, we calculated a standardised mortality ratio using the WHO suicide rate estimates and assumed this would be the equivalent to a RR. Given that ethnic minority groups make up a small proportion of the overall population, and the number of suicide deaths in this group are likely to be small in comparison to the ethnic majority population the standardised mortality ratio is likely to be equivalent to a RR. However, if these assumptions are not met our estimates may be biased towards the null. Fifth, despite us restricting our searches to papers published in the last two decades, there were papers included in our review which reported on data from the 1960s. Given that suicide rates have changed significantly over time, the inclusion of data spanning a wide period may in part contribute to the large amount of heterogeneity observed. However, in our post-hoc analysis which stratified by publication year, substantial heterogeneity persisted between study estimates. Lastly, we restricted our exploration of heterogenicity to sub-group meta-analyses. This approach does not allow us to investigate the sources of heterogeneity in detail and it would have been better to use meta-regression techniques. We did not conduct a meta-regression given that we would have had very few moderate-high quality studies with all the covariate data required.

Despite the prevailing homogenous grouping of individuals from ethnic minority backgrounds in research and the public discourse, our study provides evidence of how this practice should change in relation to suicide prevention because of high levels of heterogeneity and inherent differences amongst groups. Researchers and governments should be aware of the fallacy of aggregating ethnic minority groups and report on individual ethnic minority groups as appropriate. Ideally in categorising individuals, this should be done with relation to likely risk, and therefore should be based on self-identification of ethnicity and be combined with other pertinent risk data, for example migratory status. When data are insufficient to do this, we need to be circumspect in our interpretation of findings. Furthermore, in the absence of self-reported ethnicity data careful consideration needs to be given to possible misclassification of individuals which might masque suicide and self-harm risk. A possible strategy for overcoming this might be with the use of multiple sources of ethnicity data which may reduce likely misclassification. Clinicians and policymakers need to carefully consider this evidence when supporting individuals from ethnic minority backgrounds and creating publicly funded government resources. Some indigenous and ethnic minority groups may be at higher risk of suicide than the wider population. The reasons for this need to be better understood and appropriate policy and clinical responses must be introduced.

## Contributors

DK, NK, and MIT had the idea for the study, designed the protocol. GC, SH-S, HCG, KR, RP, RM, SB, DK, and MIT did the study selection and data extraction. GC, SB, RM, KR, DK, MIT, RP, and HCG conducted reference screening of relevant papers. MIT and DK drafted the manuscript with input received from MS, NK, GC, HCG, RP, RM, KR, and SB. MS conducted statistical analysis. RP created and managed the reference list and assisted in the development of tables and Figs. All authors critically revised the manuscript. All authors had full access to all the data in the study and had final responsibility for the decision to submit for publication.

## Data sharing statement

Our study is based on published data. The data supporting the findings of this study are available within this article and the supporting files, and all data retrieved from original papers, together with tables and Figs. arising from these data, are available to share upon reasonable request to the corresponding author.

## Funding

No specific funding was provided to conduct this research. DK is funded by Wellcome Trust and Elizabeth Blackwell Institute Bristol. Matthew Spittal is a recipient of an Australian Research Council Future Fellowship (project number FT180100075) funded by the Australian Government. Rebecca Musgrove is funded by the NIHR Greater Manchester Patient Safety Translational Research Centre (PSTRC-2016-003).

## Declaration of interests

NK reports grants and personal fees from Department of Health and Social Care, National Institute of Health Research (NIHR), National Institute of Health and Care Excellence, Healthcare Quality and Improvement Partnership, outside the submitted work; and work with NHS England on national quality improvement initiatives for suicide and self-harm. He sits on Department of Health and Social Care's (England) National Suicide Prevention Strategy Advisory Group. He has chaired and been the Topic Advisor for NICE guideline committees for Self-harm and Depression. DK reported grants and personal fees from Wellcome Trust and the Centre for Pesticide Suicide Prevention and Department of Health and Social Care (UK). DK is a steering group member of the Migration Health and Development Research Initiative, where she receives no fees for this work. RM reports PhD stipend fees paid by the NIHR Greater Manchester Patient Safety Translational research centre (PSTRC). All other authors have nothing to declare.

## Appendix Supplementary materials

Image, application 1

## References

[1] World Health Organisation. Suicide. 2021 https://www.who.int/news-room/fact-sheets/detail/suicide. Accessed 15 December 2021

[2] Abrutyn S. (2017). What Hindu sati can teach us about the sociocultural and social psychological dynamics of suicide. J Theory Soc Behav.

[3] Mishara B.L., Weisstub D.N. (2016). The legal status of suicide: a global review. Int J Law Psychiatry.

[4] Bhui K., McKenzie K., Rasul F. (2007). Rates, risk factors & methods of self harm among minority ethnic groups in the UK: a systematic review. BMC Public Health.

[5] Al-Sharifi A., Krynicki C.R., Upthegrove R. (2015). Self-harm and ethnicity: a systematic review. Int J Soc Psychiatry.

[6] Lai D.W.L., Li L., Daoust G.D. (2017). Factors influencing suicide behaviours in immigrant and ethno-cultural minority groups: a systematic review. J. Immigr. Minor Health.

[7] Montesinos A., Heinz A., Schouler-Ocak M., Aichberger M. (2013). Precipitating and risk factors for suicidal behaviour among immigrant and ethnic minority women in Europe: a systematic review. Suicidol Online.

[8] Forte A., Trobia F., Gualtieri F. (2018). Suicide risk among immigrants and ethnic minorities: a literature overview. Int J Environ Res Public Health.

[9] Sørensen J.B., Agampodi T., Sørensen B.R., Siribaddana S., Konradsen F., Rheinländer T. (2017). We lost because of his drunkenness’: the social processes linking alcohol use to self-harm in the context of daily life stress in marriages and intimate relationships in rural Sri Lanka. BMJ Glob Health.

[10] Harder H.G., Rash J., Holyk T., Jovel E., Harder K. (2020). Routledge International Handbook of Clinical Suicide Research.

[11] Pollock N.J., Naicker K., Loro A., Mulay S., Colman I. (2018). Global incidence of suicide among Indigenous peoples: a systematic review. BMC Med.

[12] Mathur R., Bhaskaran K., Chaturvedi N. (2014). Completeness and usability of ethnicity data in UK-based primary care and hospital databases. J Public Health.

[13] Department of Economic and Social Affairs, United Nations (2015). https://www.un.org/esa/socdev/unpfii/documents/2016/Docs-updates/The-State-of-The-Worlds-Indigenous-Peoples-2-WEB.pdf.

[14] Yoon E., Hacker J., Hewitt A., Abrams M., Cleary S. (2012). Social connectedness, discrimination, and social status as mediators of acculturation/enculturation and well-being. J Couns Psychol.

[15] Grigoroglou C., Munford L., Webb R.T., Kapur N., Ashcroft D.M., Kontopantelis E. (2019). Prevalence of mental illness in primary care and its association with deprivation and social fragmentation at the small-area level in England. Psychol Med.

[16] Anderson I., Robson B., Connolly M. (2016). Indigenous and tribal peoples’ health (The Lancet–Lowitja Institute Global Collaboration): a population study. Lancet.

[17] Pirkis J., John A., Shin S. (2021). Suicide trends in the early months of the COVID-19 pandemic: an interrupted time-series analysis of preliminary data from 21 countries. Lancet Psychiatry.

[18] Curtin SC, Hedegaard H, Ahmad FB. Provisional Numbers and rates of suicide by month and demographic characteristics: United States, 2020. NVSS Vital Stat Rapid Release 2020. https://www.cdc.gov/nchs/products/index.htm. Accessed 15 December 2021.

[19] Page A., Bandara P., Hammond T.E., Stevens G., Carter G. (2021). Impact of Covid-19 physical distancing policies on incidence of intentional self-harm in Western Sydney. Australas Psychiatry.

[20] Rockett I.R.H., Wang S., Stack S. (2010). Race/ethnicity and potential suicide misclassification: window on a minority suicide paradox?. BMC Psychiatry.

[21] Keyes K.M., Liu X.C., Cerda M. (2012). The role of race/ethnicity in alcohol-attributable injury in the United States. Epidemiol Rev.

[22] McKenzie K., Serfaty M., Crawford M. (2003). Suicide in ethnic minority groups. Br J Psychiatry.

[23] Aspinall P.J. (2002). Suicide amongst Irish migrants in Britain: a review of the identity and integration hypothesis. Int J Soc Psychiatry.

[24] Rees R, Stokes G, Stansfield C, Oliver E, Kneale D, Thomas J. Prevalence of mental health disorders in adult minority ethnic populations in England: a systematic review. London, UK, 2016 https://discovery.ucl.ac.uk/id/eprint/1485144/. Accessed 4 July 2021.

[25] Rehkopf D.H., Buka S.L. (2006). The association between suicide and the socio-economic characteristics of geographical areas: a systematic review. Psychol Med.

[26] Bécares L., Dewey M.E., Das-Munshi J. (2018). Ethnic density effects for adult mental health: systematic review and meta-analysis of international studies. Psychol Med.

[27] Aldridge R.W., Nellums L.B., Bartlett S. (2018). Global patterns of mortality in international migrants: a systematic review and meta-analysis. Lancet.

[28] Ouzzani M., Hammady H., Fedorowicz Z., Elmagarmid A. (2016). Rayyan-a web and mobile app for systematic reviews. Syst Rev.

[29] Amiri S. (2020). Prevalence of suicide in immigrants/refugees: a systematic review and meta-analysis. Arch Suicide Res.

[30] Azuero A.J., Arreaza-Kaufman D., Coriat J. (2017). Suicide in the indigenous population of Latin America: a systematic review. Rev Colomb Psiquiatr.

[31] Armitage C.J., Panagioti M., Abdul Rahim W., Rowe R., O'Connor R.C (2015). Completed suicides and self-harm in Malaysia: a systematic review. Gen Hosp Psychiatry.

[32] Wells G, Shea B, O'Connell D, et al. The Newcastle–Ottawa scale (NOQAS) for assessing the quality of non-randomized studies in meta-analysis. Ottawa, Canada, 2011. http://www3.med.unipmn.it/dispense_ebm/2009-2010/Corso%20Perfezionamento%20EBM_Faggiano/NOS_oxford.pdf. Accessed 20 April 2022.

[33] Knipe D., Williams A.J., Hannam-Swain S. (2019). Psychiatric morbidity and suicidal behaviour in low- and middle-income countries: a systematic review and meta-analysis. PLoS Med.

[34] Yuan T., Fitzpatrick T., Ko N.Y. (2019). Circumcision to prevent HIV and other sexually transmitted infections in men who have sex with men: a systematic review and meta-analysis of global data. Lancet Glob Health.

[35] Knipe D.W., Carroll R., Thomas K.H., Pease A., Gunnell D., Metcalfe C. (2015). Association of socio-economic position and suicide/attempted suicide in low and middle income countries in South and South-East Asia - a systematic review. BMC Public Health.

[36] Khunti K., Routen A., Pareek M., Treweek S., Platt L. (2020). The language of ethnicity. BMJ.

[37] Office for National Statistics. Suicide statistics by ethnicity. 2021 https://www.ons.gov.uk/aboutus/transparencyandgovernance/freedomofinformationfoi/suicidestatisticsbyethnicity. Accessed 16 December 2021.

[38] Harris R.J., Bradburn M.J., Deeks J.J., Altman D.G., Harbord R.M., Sterne J.A.C. (2008). Metan: fixed- and random-effects meta-analysis. Stata J.

[39] World Health Organisation (2021). https://www.who.int/data/gho/data/themes/mental-health/suicide-rates.

[40] Kobori E., Maeda Y., Yamamoto T. (2017). Mortality rates of foreign national residents in Japan: comparison with the Japanese population and a possible healthy migrant effect. Nippon Koshu Eisei Zasshi.

[41] Lash T.L., VanderWeele T.J., Haneuse S., Rothman K.J. (2021).

[42] Viechtbauer W. (2010). Conducting meta-analyses in R with the metafor package. J Stat Softw.

[43] Pavlović E., Marušič A. (2001). Suicide in Croatia and in Croatian immigrant groups in Australia and Slovenia. Croat Med J.

[44] Malenfant E.C. (2004). Suicide in Canada's immigrant population. Health Rep.

[45] Karmali S., Laupland K., Harrop A.R. (2005). Epidemiology of severe trauma among status Aboriginal Canadians: a population-based study. CMAJ.

[46] Yun S., Kayani N., Geiger S., Homan S., Wilson J. (2016). High risk behaviors but low injury-related mortality among hispanic teens in Missouri. Public Health Rep.

[47] Wissow L.S., Walkup J., Barlow A., Reid R., Kane S. (2001). Cluster and regional influences on suicide in a Southwestern American Indian tribe. Soc Sci Med.

[48] Wainiqolo I., Kafoa B., Kool B., Herman J., McCaig E., Ameratunga S. (2012). A profile of Injury in Fiji: findings from a population-based injury surveillance system (TRIP-10). BMC Public Health.

[49] Voracek M., Loibl L.M., Dervic K., Kapusta N.D., Niederkrotenthaler T., Sonneck G. (2009). Consistency of immigrant suicide rates in Austria with country-of-birth suicide rates: a role for genetic risk factors for suicide?. Psychiatry Res.

[50] Telisinghe P.U., Colombage S.M. (2014). Patterns of suicide in Brunei Darussalam and comparison with neighbouring countries in South East Asia. J Forensic Leg Med.

[51] Spicer R.S., Miller T.R. (2000). Suicide acts in 8 states: incidence and case fatality rates by demographics and method. Am J Public Health.

[52] Singh G.K., Siahpush M. (2001). All-cause and cause-specific mortality of immigrants and native born in the United States. Am J Public Health.

[53] Singh G.K., Miller B.A. (2004). Health, life expectancy, and mortality patterns among immigrant populations in the United States. Can J Public Health.

[54] Saunders N.R., Chiu M., Lebenbaum M. (2019). Suicide and self-harm in recent immigrants in Ontario, Canada: a population-based study. Can J Psychiatry.

[55] Pridmore S., Fujiyama H. (2009). Suicide in the Northern territory, 2001-2006. Aust N Z J Psychiatry.

[56] Orellana J.D., Balieiro A.A., Fonseca F.R., Basta P.C., de Souza M.L.P. (2016). Spatial-temporal trends and risk of suicide in Central Brazil: an ecological study contrasting indigenous and non-indigenous populations. Braz J Psychiatry.

[57] Bando D.H., Brunoni A.R., Fernandes T.G., Benseñor I.M., Lotufo P.A. (2012). Taxas de suicídio e tendências em São Paulo, Brasil, de acordo com gênero, faixa etária e aspectos demográficos. Rev Bras Psiquiatr.

[58] Norredam M., Olsbjerg M., Petersen J.H., Laursen B., Krasnik A. (2013). Are there differences in injury mortality among refugees and immigrants compared with native-born?. Inj Prev.

[59] Merrill R.M. (2019). Injury-related deaths according to environmental, demographic, and lifestyle factors. J Environ Public Health.

[60] Kuroki Y. (2018). Comparison of suicide rates among Asian Americans in 2000 and 2010. Omega.

[61] Kposowa A.J. (2000). Marital status and suicide in the national longitudinal mortality study. J Epidemiol Commun Health.

[62] Kõlves K., Potts B., De Leo D. (2015). Ten years of suicide mortality in Australia: socio-economic and psychiatric factors in Queensland. J Forensic Leg Med.

[63] Kanamori M., Kondo N., Juarez S., Dunlavy A., Cederström A., Rostila M. (2020). Rural life and suicide: does the effect of the community context vary by country of birth? A Swedish registry-based multilevel cohort study. Soc Sci Med.

[64] Jung H., Matei D.B., Hecser L. (2009). Biostatistical study of suicide features in Mures County (Romania). Leg Med.

[65] Jiang Y., Ranney M.L., Perez B., Viner-Brown S. (2016). Burden of violent death on years of life lost in Rhode Island, 2006–2013. Am J Prev Med.

[66] Homer C.D., Engelhart D.A., Lavins E.S., Jenkins A.J. (2005). Carbon monoxide-related deaths in a metropolitan county in the USA: an 11-year study. Forensic Sci Int.

[67] Harrop A.R., Brant R.F., Ghali W.A., Macarthur C. (2007). Injury mortality rates in native and non-native children: a population-based study. Public Health Rep.

[68] Hanlon T.J., Barber C., Azrael D., Miller M. (2019). Type of firearm used in suicides: findings from 13 states in the national violent death reporting system, 2005–2015. J Adolesc Health.

[69] Goosen S., Kunst A.E., Stronks K., Van Oostrum I.E.A., Uitenbroek D.G., Kerkhof A.J.F.M. (2011). Suicide death and hospital-treated suicidal behaviour in asylum seekers in the Netherlands: a national registry-based study. BMC Public Health.

[70] Garlow S.J., Purselle D.C., Heninger M. (2007). Cocaine and alcohol use preceding suicide in African American and white adolescents. J Psychiatr Res.

[71] Elo I.T., Beltrán-Sánchez H., Macinko J. (2014). The contribution of health care and other interventions to Black-White disparities in life expectancy, 1980-2007. Popul Res Policy Rev.

[72] Di Thiene D., Alexanderson K., Tinghög P., La Torre G., Mittendorfer-Rutz E (2015). Suicide among first-generation and second-generation immigrants in Sweden: association with labour market marginalisation and morbidity. J Epidemiol Commun Health.

[73] Deckert A., Winkler V., Meisinger C., Heier M., Becher H. (2015). Suicide and external mortality pattern in a cohort of migrants from the former Soviet Union to Germany. J Psychiatr Res.

[74] De Leo D., Sveticic J., Milner A. (2011). Suicide in indigenous people in Queensland, Australia: trends and methods, 19942007. Aust N Z J Psychiatry.

[75] Day G.E., Provost E., Lanier A.P. (2009). Alaska native mortality rates and trends. Public Health Rep.

[76] Brennecke G., Stoeber F.S., Kettner M. (2020). Suicide among immigrants in Germany. J Affect Disord.

[77] Wong C.A., Gachupin F.C., Holman R.C. (2014). American Indian and Alaska native infant and pediatric mortality, United States, 1999–2009. Am J Public Health.

[78] Garlow S.J., Purselle D., Heninger M. (2005). Ethnic differences in patterns of suicide across the life cycle. Am J Psychiatry.

[79] Kõlves K., De Leo D. (2015). Are immigrants responsible for the recent decline in Australian suicide rates?. Epidemiol Psychiatr Sci.

[80] Burrows S., Laflamme L. (2006). Suicide mortality in South Africa: a city-level comparison across socio-demographic groups. Soc Psychiatry Psychiatr Epidemiol.

[81] Burrows S., Auger N., Tamambang L., Barry A.D. (2013). Suicide mortality gap between Francophones and Anglophones of Quebec, Canada. Soc Psychiatry Psychiatr Epidemiol.

[82] Bauwelinck M., Deboosere P., Willaert D., Vandenheede H. (2017). Suicide mortality in Belgium at the beginning of the 21st century: differences according to migrant background. Eur J Public Health.

[83] Stockard J., O'Brien R.M (2002). Cohort variations and changes in age-specific suicide rates over time: explaining variations in youth suicide. Soc Forces.

[84] Shah A., Lindesay J., Dennis M. (2009). Comparison of elderly suicide rates among migrants in England and Wales with their country of origin. Int J Geriatr Psychiatry.

[85] Westman J., Sundquist J., Johansson L.M., Johansson S.E., Sundquist K. (2006). Country of birth and suicide: a follow-up study of a national cohort in Sweden. Arch Suicide Res.

[86] Liu I.C., Liao S.F., Lee W.C., Kao C.Y., Jenkins R., Cheng A.T.A. (2011). A cross-ethnic comparison on incidence of suicide. Psychol Med.

[87] Cwik M.F., Tingey L., Maschino A. (2016). Decreases in suicide deaths and attempts linked to the white mountain apache suicide surveillance and prevention system, 2001-2012. Am J Public Health.

[88] Dunlavy A.C., Juárez S., Toivanen S., Rostila M. (2019). Suicide risk among native- and foreign-origin persons in Sweden: a longitudinal examination of the role of unemployment status. Soc Psychiatry Psychiatr Epidemiol.

[89] Wen C.P., Tsai S.P., Shih Y.T., Chung W.S.I. (2004). Bridging the gap in life expectancy of the aborigines in Taiwan. Int J Epidemiol.

[90] Singh G.K., Hiatt R.A. (2006). Trends and disparities in socioeconomic and behavioural characteristics, life expectancy, and cause-specific mortality of native-born and foreign-born populations in the United States, 1979-2003. Int J Epidemiol.

[91] Tuck A., Bhui K., Nanchahal K., McKenzie K. (2011). Suicide by burning in the South Asian origin population in England and Wales a secondary analysis of a national data set. BMJ Open.

[92] Abdalla S., Kelleher C.C., Quirke B., Daly L. (2013). Disparities in fatal and non-fatal injuries between Irish travellers and the Irish general population are similar to those of other indigenous minorities: a cross-sectional population-based comparative study. BMJ Open.

[93] Loh M., Tan C.H., Sim K. (2007). Epidemiology of completed suicides in Singapore for 2001 and 2002. Crisis.

[94] Herman J., Peiris-John R., Wainiqolo I. (2016). Epidemiology of fatal and hospitalised injuries among youth in Fiji (TRIP 15). J Paediatr Child Health.

[95] Soole R., Kõlves K., De Leo D. (2014). Factors related to childhood suicides: analysis of the queensland child death register. Crisis.

[96] Ougrin D., Banarsee R., Dunn-Toroosian V., Majeed A. (2011). Suicide survey in a London borough: primary care and public health perspectives. J Public Health.

[97] Mittendorfer-Rutz E., Hagström A., Hollander A.C. (2020). High suicide rates among unaccompanied minors/youth seeking asylum in Sweden. Crisis.

[98] Hjern A., Allebeck P. (2002). Suicide in first- and second-generation immigrants in Sweden. A comparative study. Soc Psychiatry Psychiatr Epidemiol.

[99] Ide N., Kõlves K., Cassaniti M., De Leo D. (2012). Suicide of first-generation immigrants in Australia, 1974-2006. Soc Psychiatry Psychiatr Epidemiol.

[100] Sanford C., Marshall S.W., Martin S.L. (2006). Deaths from violence in North Carolina, 2004: how deaths differ in females and males. Inj Prev.

[101] Gilmour S., Hoshino H., Dhungel B. (2019). Suicide mortality in foreign residents of Japan. Int J Environ Res Public Health.

[102] Iribarren C., Sidney S., Jacobs D.R., Weisner C. (2000). Hospitalization for suicide attempt and completed suicide: epidemiological features in a managed care population. Soc Psychiatry Psychiatr Epidemiol.

[103] Hoffmann J.A., Farrell C.A., Monuteaux M.C., Fleegler E.W., Lee L.K. (2020). Association of pediatric suicide with county-level poverty in the United States, 2007-2016. JAMA Pediatr.

[104] Khan S.Q., Berrington De Gonzalez A., Best A.F. (2018). Infant and youth mortality trends by race/ethnicity and cause of death in the United States. JAMA Pediatr.

[105] Hastings K.G., Jose P.O., Kapphahn K.I. (2015). Leading causes of death among Asian American subgroups (2003-2011). PLoS One.

[106] Webb R.T., Antonsen S., Mok P.L.H., Agerbo E., Pedersen C.B. (2015). National cohort study of suicidality and violent criminality among Danish immigrants. PLoS One.

[107] Ueda M., Yoshikawa K., Matsubayashi T. (2019). Suicide by persons with foreign background in Japan. PLoS One.

[108] Rockett I.R.H., Regier M.D., Kapusta N.D. (2012). Leading causes of unintentional and intentional injury mortality: united States, 2000-2009. Am J Public Health.

[109] DeMello A.S., Yang Y., Schulte J. (2020). Learning from suicide deaths in Harris County, Texas. Death Stud.

[110] Holck P., Day G.E., Provost E. (2013). Mortality trends among Alaska native people: successes and challenges. Int J Circumpolar Health.

[111] Krivo L.J., Phillips J.A. (2018). How does immigration affect suicide? An analysis of U.S. metropolitan areas. Soc Sci Q.

[112] Lazzarini T.A., Gonçalves C.C.M., Benites W.M. (2017). Suicide in Brazilian indigenous communities: clustering of cases in children and adolescents by household. Rev Saude Publica.

[113] Pathak E.B. (2018). Mortality among Black Men in the USA. J Racial Ethn Health Disparities.

[114] Day G.E., Lanier A.P. (2003). Alaska native mortality, 1979–1998. Public Health Rep.

[115] Bridge J.A., Asti L., Horowitz L.M. (2015). Suicide trends among elementary school-aged children in the United States From 1993 to 2012. JAMA Pediatr.

[116] Sonderman J.S., Munro H.M., Blot W.J., Tarone R.E., McLaughlin J.K. (2014). Suicides, homicides, accidents, and other external causes of death among blacks and whites in the southern community cohort study. PLoS One.

[117] Puzo Q., Mehlum L., Qin P. (2018). Rates and characteristics of suicide by immigration background in Norway. PLoS One.

[118] Silviken A. (2009). Prevalence of suicidal behaviour among indigenous Sami in northern Norway. Int J Circumpolar Health.

[119] Price J.H., Khubchandani J. (2019). The changing characteristics of African-American Adolescent suicides, 2001–2017. J Commun Health.

[120] Pacot R., Garmit B., Pradem M., Nacher M., Brousse P. (2018). The problem of suicide among Amerindians in Camopi-Trois Sauts, French Guiana 2008-2015. BMC Psychiatry.

[121] Fairthorne J., Walker R., De Klerk N., Shepherd C. (2016). Early mortality from external causes in Aboriginal mothers: a retrospective cohort study. BMC Public Health.

[122] Pollock N.J., Healey G.K., Jong M., Valcour J.E., Mulay S. (2018). Tracking progress in suicide prevention in Indigenous communities: a challenge for public health surveillance in Canada. BMC Public Health.

[123] Stefanac N., Hetrick S., Hulbert C., Spittal M.J., Witt K., Robinson J. (2019). Are young female suicides increasing? A comparison of sex-specific rates and characteristics of youth suicides in Australia over 2004-2014. BMC Public Health.

[124] Shah A., Lindesay J., Dennis M. (2011). Suicides by country of birth groupings in England and Wales: age-associated trends and standardised mortality ratios. Soc Psychiatry Psychiatr Epidemiol.

[125] Silviken A., Haldorsen T., Kvernmo S. (2006). Suicide among indigenous Sami in Arctic Norway, 1970-1998. Eur J Epidemiol.

[126] Amin R., Helgesson M., Runeson B. (2019). Suicide attempt and suicide in refugees in Sweden-a nationwide population-based cohort study. Psychol Med.

[127] Hollander A.C., Pitman A., Sjöqvist H. (2020). Suicide risk among refugees compared with non-refugee migrants and the Swedish-born majority population. Br J Psychiatry.

[128] Martin S.L., Proescholdbell S., Norwood T., Kupper L.L. (2010). Suicide and homicide in North Carolina: initial findings from the North Carolina violent death reporting system, 2004-2007. N C Med J.

[129] Shoaf K., Sauter C., Bourque L.B., Giangreco C., Weiss B. (2004). Suicides in Los Angeles County in relation to the Northridge earthquake. Prehosp Disaster Med.

[130] Termorshuizen F., Braam A.W., van Ameijden E.J.C. (2015). Neighborhood ethnic density and suicide risk among different migrant groups in the four big cities in the Netherlands. Soc Psychiatry Psychiatr Epidemiol.

[131] Werenko D.D., Olson L.M., Fullerton-Gleason L., Lynch A.W., Zumwalt R.E., Sklar D.P. (2000). Child and adolescent suicide deaths in New Mexico, 1990-1994. Crisis.

[132] Yamall Orellana J.D., de Souza C.C., Ponte de Souza M.L (2019). Hidden suicides of the indigenous people of the Brazilian amazon: gender, alcohol and familial clustering. Rev Colomb Psiquiatr.

[133] Yau R.K., Paschall M.J. (2018). Epidemiology of asphyxiation suicides in the United States, 2005–2014. Inj Epidemiol.

[134] Ali N.H., Zainun K.A., Bahar N. (2012). Pattern of suicides in 2009: data from the National Suicide Registry Malaysia. Asia Pac Psychiatry.

[135] Kua E.H., Ko S.M., Ng T.P. (2003). Recent trendsin elderly suicide rates in a multi-ethnic Asian City. Int J Geriatr Psychiatry.

[136] Värnik A., Kõlves K., Wasserman D. (2005). Suicide among Russians in Estonia: database study before and after independence. Br Med J.

[137] Music E., Jacobsson L., Renberg E.S. (2014). Suicide in Bosnia and Herzegovina and the city of Sarajevo: with special reference to ethnicity. Crisis.

[138] Measey M.A.L., Li S.Q., Parker R., Wang Z. (2006). Suicide in the Northern Territory, 1981-2002. Med J Aust.

[139] Herne M.A., Bartholomew M.L., Weahkee R.L. (2014). Suicide mortality among American Indians and Alaska Natives, 1999-2009. Am J Public Health.

[140] EchoHawk M. (2006). Suicide prevention efforts in one area of Indian health service, USA. Arch Suicide Res.

[141] Pollock N.J., Mulay S., Valcour J., Jong M. (2016). Suicide rates in aboriginal communities in Labrador, Canada. Am J Public Health.

[142] Tian N., Zack M., Fowler K.A., Hesdorffor D.C. (2019). Suicide timing in 18 states of the United States from 2003 to 2014. Arch Suicide Res.

[143] Sumarokov Y.A., Brenn T., Kudryavtsev A.V., Nilssen O. (2014). Suicides in the indigenous and non-indigenous populations in the Nenets Autonomous Okrug, Northwestern Russia, and associated socio-demographic characteristics. Int J Circumpolar Health.

[144] Bjerregaard P., Larsen C.V.L. (2015). Time trend by region of suicides and suicidal thoughts among Greenland inuit. Int J Circumpolar Health.

[145] Maynard M.J., Rosato M., Teyhan A., Harding S. (2012). Trends in suicide among migrants in England and Wales 1979-2003. Ethn Health.

[146] Ruch D.A., Sheftall A.H., Schlagbaum P., Rausch J., Campo J.V., Bridge J.A. (2019). Trends in suicide among youth aged 10 to 19 years in the United States, 1975 to 2016. JAMA Netw Open.

[147] Rhoades E.R. (2003). The health status of American Indian and Alaska native males. Am J Public Health.

[148] Mullany B., Barlow A., Goklish N. (2009). Toward understanding suicide among youths: results from the White Mountain Apache tribally mandated suicide surveillance system, 2001-2006. Am J Public Health.

[149] Styka A.N., White D.S., Zumwalt R.E., Lathrop S.L. (2010). Trends in adult suicides in New Mexico: utilizing data from the new mexico violent death reporting system. J Forensic Sci.

[150] Arya V., Page A., Dandona R., Vijayakumar L., Mayer P., Armstrong G. (2019). The geographic heterogeneity of suicide rates in India by religion, caste, tribe, and other backward classes. Crisis.

[151] Vieweg W.V.R., Linker J.A., Anum E.A. (2005). Child and adolescent suicides in Virginia: 1987 to 2003. J Child Adolesc Psychopharmacol.

[152] Heninger M., Hanzlick R. (2008). Nonnatural deaths of adolescents and teenagers: fulton county, Georgia, 1985-2004. Am J Forensic Med Pathol.

[153] Matzopoulos R., Prinsloo M., Pillay-Van Wyk V. (2015). Injury-related mortality in south africa: a retrospective descriptive study of postmortem investigations. Bull World Health Organ.

[154] Saunders N.R., Lebenbaum M., Stukel T.A. (2017). Suicide and self-harm trends in recent immigrant youth in Ontario, 1996-2012: a population-based longitudinal cohort study. BMJ Open.

[155] Nestadt P.S., Triplett P., Fowler D.R., Mojtabai R. (2017). Urban-rural differences in suicide in the state of Maryland: the role of firearms. Am J Public Health.

[156] Matthay E.C., Galin J., Ahern J. (2017). Changing patterns in rates and means of suicide in California, 2005 to 2013. Am J Public Health.

[157] Øien-Ødegaard C., Reneflot A., Hauge L.J. (2019). Use of primary healthcare services prior to suicide in Norway: a descriptive comparison of immigrants and the majority population. BMC Health Serv Res.

[158] Kerr G.R., Ramsey D.J. (2003). Deaths of Texas adolescents from injury, 1996 through 1998. Tex Med.

[159] Pear V.A., Castillo-Carniglia A., Kagawa R.M.C., Cerdá M., Wintemute G.J (2018). Firearm mortality in California, 2000–2015: the epidemiologic importance of within-state variation. Ann Epidemiol.

[160] Ferreira M.E.V., Matsuo T., de Souza R.K.T. (2011). Aspectos demográficos e mortalidade de populações indígenas do Estado do Mato Grosso do Sul, Brasil. Cad Saude Publica.

[161] Garssen M., Hoogenboezem J., Kerkhof A. (2007). Zelfdoding onder Nederlandse Surinamers naar etniciteit. Tijdschr Psychiatr.

[162] Koppenaal H., Bos C.A., Broer J. (2003). Hoge sterfte door infectieziekten en niet-natuurlijke doodsoorzaak onder asielzoekers in 1998-1999. Ned Tijdschr Geneeskd.

[163] Razum O., Zeeb H. (2004). Suicide mortality among Turks in Germany. Nervenarzt.

[164] De Souza M.L.P. (2019). Mortality from suicide in indigenous children in Brazil. Cad Saude Publica.

[165] Bhupinder S., Kumara T.K., Syed A.M. (2010). Completed suicides in the district of Timur Laut, Penang Island - a preliminary investigation of 3 years (2007-2009) prospective data. Med J Malays.

[166] Hassler S., Johansson R., Sjölander P., Grönberg H., Damber L. (2005). Causes of death in the Sami population of Sweden, 1961-2000. Int J Epidemiol.

[167] Soininen L., Pukkala E. (2008). Mortality of the Sami in northern Finland 1979-2005. Int J Circumpolar Health.

[168] United Nations (2020). https://www.un.org/en/desa/international-migration-2020-highlights.

[169] Saunders C.L., Abel G.A., El Turabi A., Ahmed F., Lyratzopoulos G. (2013). Accuracy of routinely recorded ethnic group information compared with self-reported ethnicity: evidence from the English cancer patient experience survey. BMJ Open.

