# Supplementary material for: Suicide rates amongst individuals from ethnic minority backgrounds: A systematic review and meta-analysis
Source: eClinicalMedicine. 2022 Apr 28;47:101399. doi: 10.1016/j.eclinm.2022.101399 (PMC9065636; doi:10.1016/j.eclinm.2022.101399)

# SUPPLEMENTARY FILES

## Supplementary File 1. Search Strategy

MEDLINE

1. Human Migration/ or Migration.mp.

2. migrant.mp. or "Transients and Migrants"/

3. Migrant*.mp.

4. "Emigrants and Immigrants"/ or immigra*.mp.

5. Expatriate.mp.

6. Refugees/ or Refugee*.mp.

7. Departee.mp.

8. "Emigration and Immigration"/ or Emigr*.mp.

9. Asylum.mp.

10. Foreign-born.mp.

11. Foreign born.mp.

12. foreign worker.mp.

13. foreign student.mp.

14. International student.mp.

15. Minority Groups/

16. Minority Health/

17. Population Groups/

18. Ethnic Groups/

19. (ethnic* or multi-ethnic* or multiethnic* or indig* or aborig* or native* or inuit* or eskimo* or kalaallit* or amerind* or romany or romani* or gypsies or gipsies or travel?er or me?tis or first?nation*).ti,ab,kf,hw.

20. (african or africaan or caribbean* or afro-caribbean* or afrocaribbean* or asian* or bangladeshi* or chinese or indian* or pakistani* or somali* or latin* or hispanic*).ti,ab,kf,hw.

21. (black or blacks).ti,af,kf,hw.

22. (BME or BAME).ti,ab,kf.

23. CALD.ti,ab,kf.

24. Cultural diversity/

25. Cross Cultural Comparison/

26. (cultural or culture or race or racial or nonwhite? or non-white?).ti,ab,kf.

27. minority.ti,ab,kf.

28. or/1-27

29. (self?harm* or suicide).ab. or (self?harm* or suicide).ti.

30. 28 and 29

31. limit 30 to yr="2000 -Current"

EMBASE

1. Migration/ or migration.mp.

2. migrant*.mp.

3. (immigra* or Emigra*).mp.

4. Expatriate.mp.

5. Refugee/ or Refugee*.mp.

6. Departee.mp.

7. Asylum.mp.

8. Foreign-born.mp.

9. Foreign born.mp.

10. foreign worker.mp.

11. foreign student.mp.

12. International student.mp.

13. minority group/

14. minority health/

15. Population Groups/

16. Ethnic Groups/

17. (ethnic* or multi-ethnic* or multiethnic* or indig* or aborig* or native* or inuit* or eskimo* or kalaallit* or amerind* or romany or romani* or gypsies or gipsies or travel?er or me?tis or first?nation*).ti,ab,kw,hw.

18. (african or africaan or caribbean* or afro-caribbean* or afrocaribbean* or asian* or bangladeshi* or chinese or indian* or pakistani* or somali* or latin* or hispanic*).ti,ab,kw,hw.

19. (black or blacks).ti,ab,kw,hw.

20. (BME or BAME).ti,ab,kw.

21. CALD.ti,ab,kw.

22. cultural diversity/

23. (race or racial or nonwhite? or non-white?).ti,ab,kw.

24. minority.ti,ab,kw.

25. or/1-24

26. (self?harm* or suicide).ab. or (self?harm* or suicide).ti.

27. 25 and 26

28. limit 27 to yr="2000 -Current"

PSYCHINFO

1. Human Migration/ or migration.mp.

2. Migrant*.mp.

3. (immigra* or Emigra*).mp.

4. Expatriate.mp.

5. refugee.mp. or Refugees/

6. Departee.mp.

7. Asylum.mp.

8. Foreign-born.mp.

9. Foreign born.mp.

10. foreign worker.mp.

11. foreign student.mp.

12. International student.mp.

13. Population Groups/

14. Ethnic Groups/

15. "Racial and Ethnic Differences"/ or "Racial and Ethnic Groups"/

16. (ethnic* or multi-ethnic* or multiethnic* or indig* or aborig* or native* or inuit* or eskimo* or kalaallit* or amerind* or romany or romani* or gypsies or gipsies or travel?er or me?tis or first?nation*).ti,ab,hw.

17. (african or africaan or caribbean* or afro-caribbean* or afrocaribbean* or asian* or bangladeshi* or chinese or indian* or pakistani* or somali* or latin* or hispanic*).ti,ab,hw.

18. (black or blacks).ti,ab.

19. (BME or BAME).ti,ab.

20. CALD.ti,ab.

21. Cross Cultural Differences/

22. (race or racial or nonwhite? or non-white?).ti,ab.

23. minority.ti,ab.

24. or/1-23

25. (self?harm* or suicide).ab. or (self?harm* or suicide).ti.

26. 24 and 25

27. limit 26 to yr="2000 -Current"

## Supplementary File 2. Exclusion criteria for studies at full-text stage

Studies which reached full-text stage but did not provide data which allowed for pooling of estimates were excluded:

- Total number of deaths and total number of person years
- Absolute rate and confidence intervals or standard deviation (SD)
- Relative risk (rate ratio) and confidence intervals or SD
- Standardised mortality ratio (SMR) and confidence intervals or number of deaths
- Total number of deaths and crude rate (allowing for back calculation of underlying population denominator and therefore confidence intervals/standard errors).

Study authors of articles with missing data published within the last 5 years (2015-2020) were contacted to request missing information (n=44).

## Supplementary File 3. Data extraction sheet

The data extraction sheet was saved in a Google Form, where reviewers would collect the following information on studies:

- Extractor name
- Study author surname, year of publication and study ID
- Study type
- Quality assessment rating
- Year of study
- Year data extracted for
- Country
- Setting
- Total number of participants
- Method of suicide
- Number of male/female participants
- Description of data source
- Description of how ethnicity was collected
- Age range
- Summary of age range
- Age group
- Measures reported for each ethnic group
- Data by ethnic groups
  - Name of ethnic minority group
  - Subcategory
  - Data presented by sex (yes/no)
  - Total number (males/females/total)
  - Total number suicides (males/females/total)
  - Absolute rate including 95% CIs (males/females/total)
  - Relative rate including 95% CIs (males/females/total)

## Supplementary File 4. Quality Assessment Criteria

| Study design | Criteria | Quality rating | | |
| --- | --- | --- | --- | --- |
|  |  | Low | Moderate | High |
| Cohort | Selection - ethnicity | Selected group of users eg nurses, volunteers or no description of the derivation of the cohort | Truly/somewhat representative of the minority ethnic group(s) under investigation | |
|  |  | Proxy used e.g. names or place of birth or no description | Secure records used to identify ethnicity (e.g., census or other official records, or self-report) | |
|  | Outcome - suicide | Self-report or no description | Official records | |
| Case series | Selection – suicide | Self report by someone else or other method or no description | Official records | |
|  |  | Potential for selection biases or not stated | Consecutive series of cases (stated/assumed) | |
|  | Exposure - ethnicity | Proxy used e.g. names or place of birth or no description | Death certificate/ coroner records but not linked to other source | Secure record collected prior to death (e.g., census) or self-reported |
| Case-control | Selection – suicide | Self-report or no description | Independent validation | |
|  |  | Potential for selection biases or not stated | Consecutive or obviously representative series | |
|  | Selection – controls | Hospital controls or no description | Community controls | |
|  | Exposure – ethnicity | Interview not blinded to case/control status or written self report or medical record only or no description | Secure records (e.g., medical) or through a structured interview where interviewer is blind to case/control status | |
|  |  | Different method of exposure ascertainment for cases and controls | Same method of exposure ascertainment for cases and controls | |
|  |  | Non respondents described or rate different and no designation | Non-response similar for cases and controls | |
| Cross-sectional | Selection - ethnicity | Selected group of users eg nurses, volunteers or no description of the derivation of the cohort | Truly/somewhat representative of the minority ethnic group(s) under investigation | |
|  |  | Not justified or described | Sample size justified and satisfactory | |
|  |  | Proxy used e.g. names or place of birth or no description | Secure records used to identify ethnicity (e.g., census or other official records, or self-report) | |
|  |  | The response rate is unsatisfactory, or the comparability between respondents and non-respondents is unsatisfactory or no description | Comparability between respondents and non-respondents characteristics established, and response rate satisfactory. | |
|  | Outcome - suicide | Self report by someone else or other method or no description | Record linkage | |

## Supplementary File 5. Description of included studies by country


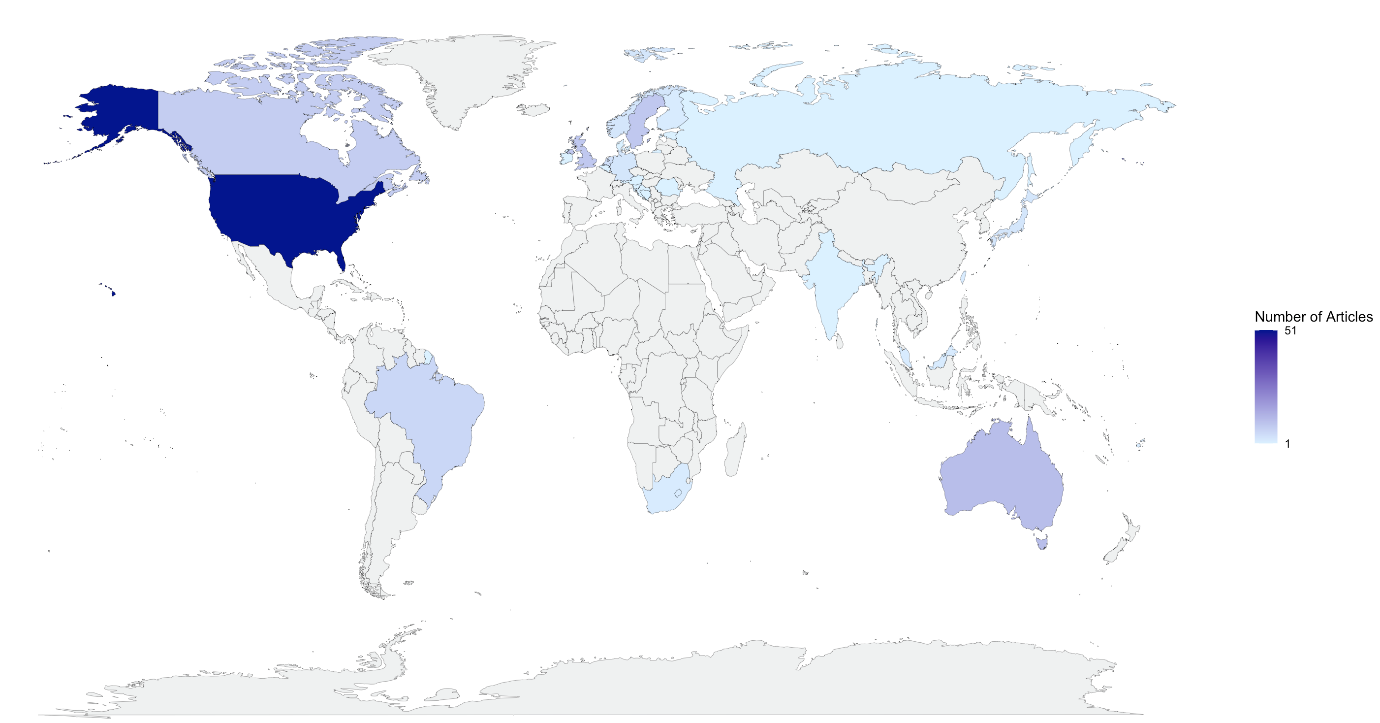


## Supplementary File 6. Reasons for exclusion full text articles (n=791)

| **No.** | **Study** | **Reason for exclusion*** |
| --- | --- | --- |
| 1 | Abd-Elwahab Hassan D, Ghaleb SS, Kotb H, Agamy M, Kharoshah M. Suicidal hanging in Kuwait: Retrospective analysis of cases from 2010 to 2012. Journal of Forensic and Legal Medicine. 2013;20(8):1118-21. | No ethnicity data |
| 2 | Abdalla S, Quirke B, Daly L, Fitzpatrick P, Kelleher C. All Ireland traveller health study: Differentials in cause-specific mortality between Irish travellers and the general population in the Republic of Ireland. Irish Journal of Medical Science.180(6):S217. | No ethnicity data |
| 3 | Abe K, Mertz KJ, Powell KE, Hanzlick RL. Characteristics of Black and White suicide decedents in Fulton County, Georgia 1988-2002. American Journal of Public Health.96(10):1794-8. | No extractable data |
| 4 | Abel WD, Bourne PA, Hamil HK, Thompson EM, Martin JS, Gibson RC, et al. A public health and suicide risk in Jamaica from 2002 to 2006. North American Journal of Medical Sciences. 2009;1(3):142-7. | No ethnicity data |
| 5 | Acciai F, Noah AJ, Firebaugh G. Pinpointing the sources of the Asian mortality advantage in the USA. Journal of epidemiology and community health.69(10):1006-11. | No extractable data |
| 6 | Adinkrah M. Patterns of female suicidal behavior in Ghana. Psychological Reports.109(2):649-62. | No ethnicity data |
| 7 | Adler N, Cattuto C, Kalimeri K, Paolotti D, Tizzoni M, Verhulst S, et al. How Search Engine Data Enhance the Understanding of Determinants of Suicide in India and Inform Prevention: Observational Study. Journal of Medical Internet Research. 2019;21(1):e10179. | No ethnicity data |
| 8 | Affleck W, Chachamovich E, Chawky N, Beauchamp G, Turecki G, Seguin M. Suicide amongst the inuit of nunavut: An exploration of life trajectories. International Journal of Environmental Research and Public Health. 2020;17(6). | No data on suicide |
| 9 | Afroz B, Moniruzzaman S, Stark Ekman D, Andersson R. The impact of economic crisis on injury mortality: The case of the 'Asian crisis'. Public Health.126(10):836-8. | No ethnicity data |
| 10 | Aghazadeh J, Motlagh ME, EntezarMahdi R, Eslami M, Mohebbi I, Yousefzadeh H, et al. Cause-specific mortality among women of reproductive age: Results from a population-based study in an Iranian community. Sexual & reproductive healthcare: official journal of the Swedish Association of Midwives. 2017;14:7-12. | No ethnicity data |
| 11 | Ahmadi A, Mohammadi R, Stavrinos D, Almasi A, Schwebel DC. Self-immolation in Iran. Journal of Burn Care and Research.29(3):451-60. | No ethnicity data |
| 12 | Ahn J. Depression, suicide, and Korean society. [Korean]. Journal of the Korean Medical Association.55(4):320-1. | No extractable data |
| 13 | Ai Ansari A, Hamadeh RR, Ali MK, El Offi A. Suicide in Bahrain in the last decade. Crisis. 2007;28(1):11-5. | No ethnicity data |
| 14 | Aichberger MC. Rates of suicidal behavior among immigrants and ethnic minorities in Europe. van Bergen, Diana D [Ed]; Heredia Montesinos, Amanda [Ed]; Schouler-Ocak, Meryam [Ed] (2015) Suicidal behavior of immigrants and ethnic minorities in Europe (pp 13-24) vii, 189 pp Boston, MA, US: Hogrefe Publishing; US. 2015:13-24. | Grey literature |
| 15 | Aktas SG. An unobtrusive important issue in the world, female suicide: Understanding geography of suicide for suicide prevention policy (case of Turkey). Ercetin, Sefika Sule [Ed]; Banerjee, Santo [Ed] (2014) Chaos and complexity theory in world politics (pp 115-146) xxiv, 383 pp Hershey, PA, US: Information Science Reference/IGI Global; US. 2014:115-46. | Grey literature |
| 16 | Al Madni OM, Azim Kharosha MA, Zaki MK, Murty OP. Trends of suicide in Dammam Kingdom of Saudi Arabia. Journal of Forensic Medicine and Toxicology.27(2):56-60. | No extractable data |
| 17 | Al Madni OM, Kharoshah MA, Zaki MK, Ghaleb SS. Hanging deaths in Dammam, Kingdom of Saudi Arabia. Journal of Forensic & Legal Medicine. 2010;17(5):265-8. | No extractable data |
| 18 | Al-Sharifi A, Krynicki CR, Upthegrove R. Self-harm and ethnicity: A systematic review. The International journal of social psychiatry.61(6):600-12. | No extractable data |
| 19 | Al-Waheeb S, Al-K, ary N. Patterns of suicide in Kuwait: a retrospective descriptive study from 2003-2009. BMC public health.15:527. | No extractable data |
| 20 | Alcantara C, Gone JP. Suicide in Native American communities: A transactional-ecological formulation of the problem. Leong, Frederick T L [Ed]; Leach, Mark M [Ed] (2008) Suicide among racial and ethnic minority groups: Theory, research, and practice (pp 173-199) xviii, 334 pp New York, NY, US: Routledge/Taylor & Francis Group; US. 2008:173-99. | Grey literature |
| 21 | Alex, er D. A comparison of suicide predictors between African Americans and Caucasians. Dissertation Abstracts International: Section B: The Sciences and Engineering. 2010;70(8):5148. | Grey literature |
| 22 | Ali B, Rockett I, Miller T. Variable Circumstances of Suicide Among Racial/Ethnic Groups by Sex and Age: A National Violent-Death Reporting System Analysis. Archives of suicide research : official journal of the International Academy for Suicide Research.1-13. | No ethnicity data |
| 23 | Alimohammadi AM, Mehrpisheh S, Memarian A. Epidemiology of cases of suicide due to hanging who referred to forensic center of Shahriar in 2011. International Journal of Medical Toxicology and Forensic Medicine. 2013;3(4):121-5. | Not general population |
| 24 | Allard YE, Wilkins R, Berthelot JM. Premature mortality in health regions with high aboriginal populations. Health reports / Statistics Canada, Canadian Centre for Health Information = Rapports sur la sante / Statistique Canada, Centre canadien d'information sur la sante.15(1):51-60. | No ethnicity data |
| 25 | Allison S, Bastiampillai T, Looi JCL, Tavella A. A tale of two cities: Suicide rates in Sydney and Melbourne are consistently lower than the rest of Australia. Australian and New Zealand Journal of Psychiatry.54(3):321-2. | No ethnicity data |
| 26 | Alrashdi S, Riyaz R, Hon S, Geller RJ, Herrington L, Kazzi ZN. Racial and ethnic differences in poisoning exposures reported to a regional poison center. Clinical Toxicology.49:612. | No extractable data |
| 27 | Altindag A, Ozkan M, Oto R. Suicide in Batman, southeastern Turkey. Suicide & Life-Threatening Behavior. 2005;35(4):478-82. | No ethnicity data |
| 28 | Anderson RN, Arias E. The effect of revised populations on mortality statistics for the United States, 2000. National vital statistics reports : from the Centers for Disease Control and Prevention, National Center for Health Statistics, National Vital Statistics System.51(9):1-24. | No ethnicity data |
| 29 | Andrade JKL. Clinical implications of the prediction of suicide attempts for native Hawaiian and non-native Hawaiian adolescents in Hawaii. Journal of the American Academy of Child and Adolescent Psychiatry.55:S9-S10. | Grey literature |
| 30 | Andrea P, Jessica L. Mortality in a Six-Year Cohort of Venue-Recruited Youth in San Francisco, California USA. Turkish Archives of Pediatrics.48:31. | Not general population |
| 31 | Andres AR, Collings S, Qin P. Sex-specific impact of socio-economic factors on suicide risk: a population-based case-control study in Denmark. European journal of public health.20(3):265-70. | No ethnicity data |
| 32 | Anikeeva O, Bi P, Hiller JE, Ryan P, Roder D, Han GS. Trends in migrant mortality rates in Australia 1981-2007: a focus on the National Health Priority Areas other than cancer. Ethnicity & health. 2015;20(1):29-48. | No ethnicity data |
| 33 | Anne George M, McCormick R, Lalonde CE, Jin A, Brussoni M. The RISC research project: Injury in First Nations communities in British Columbia, Canada. International Journal of Circumpolar Health. 2013;72. | No ethnicity data |
| 34 | Anonymous. Injury mortality among American Indian and Alaska Native children and youth--United States, 1989-1998. Mmwr. (30):697-701. | Grey literature |
| 35 | Anonymous. Suicide among Hispanics--United States, 1997-2001. Mmwr. (22):478-81. | Grey literature |
| 36 | Anonymous. Alcohol and suicide among racial/ethnic populations - 17 states, 2005-2006. Mmwr. (23):637-41. | Grey literature |
| 37 | McKenzie K, Bhui K, Nanchahal K, Blizard B.. Suicide rates in people of South Asian origin in England and Wales: 1993-2003 (BJP 193 (406-409)). British Journal of Psychiatry.194(5):470. | No extractable data |
| 38 | Anonymous. Epidemiology of suicide and suicidal behavior in children and adolescents. Iranian Journal of Psychiatry.7(4):51. | No extractable data |
| 39 | Anonymous. QuickStats: Age-Adjusted Suicide Rates by Race/Ethnicity - National Vital Statistics System, United States, 2015-2016. Mmwr. (14):433. | Grey literature |
| 40 | Anonymous. Suicide among adults aged 35-64 Years - United States, 1999-2010. Morbidity and Mortality Weekly Report. 2013;62(17):321-5. | Grey literature |
| 41 | Anonymous. QuickStats: Age-Adjusted Suicide Rates, <sup>,</sup><sup> </sup> by Race/Ethnicity - National Vital Statistics System, United States, 2015-2016. MMWR - Morbidity & Mortality Weekly Report. 2018;67(14):433. | Grey literature |
| 42 | Anton S, ic N, Pozgain I. Suicides in the Osijek area. Socijalna Psihijatrija. 2000;28(4):155-61. | No extractable data |
| 43 | Apter A, King RA, Bleich A, Fluck A, Kotler M, Kron S. Fatal and non-fatal suicidal behavior in Israeli adolescent males. Archives of Suicide Research.12(1):20-9. | Not general population |
| 44 | Aragon TJ, Lichtensztajn DY, Katcher BS, Reiter R, Katz MH. Calculating expected years of life lost for assessing local ethnic disparities in causes of premature death. BMC Public Health. 2008;8. | No data on suicide |
| 45 | Arias E, MacDorman MF, Strobino DM, Guyer B. Annual Summary of Vital Statistics - 2002. Pediatrics.112(6):1215-30. | Not general population |
| 46 | Arias SA, Miller I, Camargo CA, Sullivan AF, Goldstein AB, Allen MH, et al. Factors associated with suicide outcomes 12 months after screening positive for suicide risk in the emergency department. Psychiatric Services.67(2):206-13. | Not general population |
| 47 | Armed Forces Health Surveillance C. Deaths by suicide while on active duty, active and reserve components, U.S. Armed Forces, 1998-2011. Msmr.19(6):7-10. | Not general population |
| 48 | Armitage CJ, Panagioti M, Abdul Rahim W, Rowe R, O'Connor RC. Completed suicides and self-harm in Malaysia: A systematic review. General Hospital Psychiatry.37(2):153-65. | No extractable data |
| 49 | Armstrong G, Spittal MJ, Jorm AF. Are we underestimating the suicide rate of middle and older-aged Indigenous Australians? An interaction between 'unknown' Indigenous status and age. Australian and New Zealand journal of public health.42(4):412-3. | Grey literature |
| 50 | Arnautovska U, McPhedran S, De Leo D. Differences in characteristics between suicide cases of farm managers compared to those of farm labourers in Queensland, Australia. Rural and remote health.15(3):3250. | Not general population |
| 51 | Arsenijevic J, Contenta A, Moissaing S, Ponthie A, Malvisi L, Zamatto F, et al. A crisis of protection and humane treatment: Violence, physical trauma and deaths among migrants/refugees travelling along the Western Balkan corridor to Northern Europe. Tropical Medicine and International Health.22:92. | Not general population |
| 52 | Auerswald CL, Lin JS, Parriott A. Six-year mortality in a street-recruited cohort of homeless youth in San Francisco, California. PeerJ. 2016;2016(4). | Not general population |
| 53 | Austin AE, Van Den Heuvel C, Byard RW. Causes of community suicides among indigenous South Australians. Journal of Forensic and Legal Medicine.18(7):299-301. | No extractable data |
| 54 | Aydin B, Kartal M. Suicide cases in a province (Samsun) of Blacksea region of Turkey between 1999-2003 years. [Turkish]. Turkiye Klinikleri Journal of Medical Sciences. 2010;30(3):1067-72. | No ethnicity data |
| 55 | Azrael D, Hemenway D, Miller M, Barber CW, Schackner R. Youth Suicide: Insights from 5 Years of Arizona Child Fatality Review Team Data. Suicide and Life-Threatening Behavior.34(1):36-43. | No ethnicity data |
| 56 | Azuero AJ, Arreaza-Kaufman D, Coriat J, Tassinari S, Faria A, Castaneda-Cardona C, et al. Suicide in the Indigenous Population of Latin America: A Systematic Review. Revista Colombiana de Psiquiatria.46(4):237-42. | No ethnicity data |
| 57 | Azuero AJ, Arreaza-Kaufman D, Coriat J, Tassinari S, Faria A, Castaneda-Cardona C, et al. Suicide in Latin American indigenous population: A systematic review. Value in Health.20:A305. | No extractable data |
| 58 | Baca-Garcia E, Perez-Rodriguez MM, Mann JJ, Oquendo MA. Suicidal Behavior in Young Women. Psychiatric Clinics of North America.31(2):317-31. | No extractable data |
| 59 | Bahar N, Ismail WS, Hussain N, Haniff J, Bujang MA, Hamid AM, et al. Suicide among the youth in Malaysia: What do we know? Asia-Pacific psychiatry : Official Journal of the Pacific Rim College of Psychiatrists. 2015;7(2):223-9. | No ethnicity data |
| 60 | Balaratnasingam S. The Kimberley suicide data: Implications for suicide prevention. Australian and New Zealand Journal of Psychiatry.51:48-9. | No extractable data |
| 61 | Balica E, Stockl H. Homicide-suicides in Romania and the role of migration. European Journal of Criminology.13(4):517-34. | Not general population |
| 62 | Balis T, Postolache TT. Ethnic differences in adolescent suicide in the United States. International Journal of Child Health and Human Development. 2008;1(3):281-96. | No extractable data |
| 63 | Barnes DH. Suicide. Hampton, Robert L [Ed]; Gullotta, Thomas P [Ed]; Crowel, Raymond L [Ed] (2010) Handbook of African American health (pp 444-460) xi, 612 pp New York, NY, US: Guilford Press; US. 2010:444-60. | Grey literature |
| 64 | Barranco RE. Suicide, religion, and Latinos: A macrolevel study of U.S. Latino suicide rates. The Sociological Quarterly.57(2):256-81. | No data on suicide |
| 65 | Barron SL. Suicide in context: Elderly Chinese female suicide in San Francisco from 1987 through 1996. Dissertation Abstracts International: Section B: The Sciences and Engineering.61(3):1624. | Grey literature |
| 66 | Barth SK, Kang HK, Bullman T. All-cause mortality among US veterans of the persian gulf war: 13-year follow-up. Public Health Reports.131(6):822-30. | No ethnicity data |
| 67 | Basta M, Vgontzas A, Kastanaki A, Michalodimitrakis M, Kanaki K, Koutra K, et al. Suicide rates in Crete, Greece during the economic crisis: The effect of age, gender, unemployment and mental health service provision. BMC Psychiatry. 2018;18(1). | No ethnicity data |
| 68 | Bastos MJ, Pereira JA, Smarzaro DC, Costa EF, Bossanel RC, Oliosa DM, et al. Ecological analysis of accidents and lethal violence in Vitoria, Southeastern Brazil. Revista de saude publica.43(1):123-32. | No ethnicity data |
| 69 | Basu R, Gavin L, Pearson D, Ebisu K, Malig B. Examining the Association between Apparent Temperature and Mental Health-Related Emergency Room Visits in California. American Journal of Epidemiology.187(4):726-35. | Not general population |
| 70 | Beaudoin V, Seguin M, Chawky N, Affleck W, Chachamovich E, Turecki G. Protective factors in the inuit population of nunavut: A comparative study of people who died by suicide, people who attempted suicide, and people who never attempted suicide. International Journal of Environmental Research and Public Health. 2018;15(1). | No extractable data |
| 71 | Beautrais A. Suicide in Asia. Crisis: The Journal of Crisis Intervention and Suicide Prevention. 2006;27(2):55-7. | No ethnicity data |
| 72 | Beautrais AL, Fergusson DM. Indigenous suicide in New Zealand. Archives of Suicide Research.10(2):159-68. | No extractable data |
| 73 | Bennett AT, Collins KA. Suicide: A ten-year retrospective study. Journal of Forensic Sciences. 2000;45(6):1256-8. | No extractable data |
| 74 | Bennett AT, Collins KA. Elderly suicide: A 10-year retrospective study. American Journal of Forensic Medicine and Pathology. 2001;22(2):169-72. | No extractable data |
| 75 | berg J. Alcohol and suicide in eastern Europe. Drug & Alcohol Review. 2008;27(4):361-73. | No ethnicity data |
| 76 | Berkelmans G, van der Mei R, Bhulai S, Merelle S, Gilissen R. Demographic risk factors for suicide among youths in the netherlands. International Journal of Environmental Research and Public Health. 2020;17(4). | No extractable data |
| 77 | Berman M. Suicide among young Alaska Native men: community risk factors and alcohol control. American journal of public health.104:S329-35. | No extractable data |
| 78 | Bernard SJ, Paulozzi LJ, Wallace DL. Fatal injuries among children by race and ethnicity--United States, 1999-2002. Mmwr. (5):1-16. | Grey literature |
| 79 | Best AF, Haozous EA, Berrington de Gonzalez A, Chernyavskiy P, Freedman ND, Hartge P, et al. Premature mortality projections in the USA through 2030: a modelling study. The lancet Public Health. 2018;3(8):e374-e84. | No extractable data |
| 80 | Bhise M. A case control psychological autopsy of farmers' suicides in Wardha District of central India. Indian Journal of Psychiatry.54:S3. | No ethnicity data |
| 81 | Bhui KS, Dinos S, McKenzie K. Ethnicity and its influence on suicide rates and risk. Ethnicity and Health.17(1):141-8. | No extractable data |
| 82 | Bhui KS, McKenzie K. Rates and risk factors by ethnic group for suicides within a year of contact with mental health services in England and Wales. Psychiatric Services.59(4):414-20. | Not general population |
| 83 | Bjerregaard P, Lynge I. Suicide - A challenge in modern Greenland. Archives of Suicide Research.10(2):209-20. | No ethnicity data |
| 84 | Bjorkenstam E, Helgesson M, Amin R, Mittendorfer-Rutz E. Mental disorders, suicide attempt and suicide: differences in the association in refugees compared with Swedish-born individuals. The British journal of psychiatry : the journal of mental science.1-7. | No data on suicide |
| 85 | Bjorksten KS, Bjerregaard P. Season of birth is different in Inuit suicide victims born into Traditional than into Modern Lifestyle: A register study from Greenland. BMC Psychiatry. 2015;15(1). | No ethnicity data |
| 86 | Black S, A. r, Gallaway M, Bell MR, Ritchie EC. Prevalence and risk factors associated with suicides of Army soldiers 2001-2009. Military Psychology.23(4):433-51. | Not general population |
| 87 | Blakely T, Atkinson J, O'Dea D. No association of income inequality with adult mortality within New Zealand: A multi-level study of 1.4 million 25-64 year olds. Journal of Epidemiology and Community Health.57(4):279-84. | No ethnicity data |
| 88 | Bobes J, Saiz P, G-Portilla M, Bascaran M, Martinez S, Paredes B, et al. Suicidal Behaviour in Asturias (Spain). Schmidtke, Armin [Ed]; Bille-Brahe, Unni [Ed]; DeLeo, Diego [Ed]; Kerkhof, Ad [Ed] (2004) Suicidal behaviour in Europe: Results from the WHO/EURO Multicentre Study on Suicidal Behaviour (pp 241-247) xii, 288 pp Ashland, OH, US: Hogrefe & Huber Publishers; US. 2004:241-7. | No ethnicity data |
| 89 | Bokhan N. Migration and mental health of aborigines of Siberia: New challenges of old problems. European Psychiatry Conference: 21st European Congress of Psychiatry, EPA. 2013;28. | No data on suicide |
| 90 | Bolling K, Tran T, Karlnoski R, Taylor L, Brown Maynell K, Smith D. Completed and attempted suicide and self-inflicted burn suicide: A comparison of risk factors. Journal of Burn Care and Research.39:S117. | No extractable data |
| 91 | Booth H. The evolution of epidemic suicide on Guam: context and contagion. Suicide & life-threatening behavior.40(1):1-13. | No ethnicity data |
| 92 | Boothroyd LJ, Kirmayer LJ, Spreng S, Malus M, Hodgins S. Completed suicides among the Inuit of northern Quebec, 1982-1996: A case-control study. Cmaj. 2001;165(6):749-55. | No extractable data |
| 93 | Borrill J, Taylor DA. Suicides by foreign national prisoners in England and Wales 2007: Mental health and cultural issues. Journal of Forensic Psychiatry and Psychology. 2009;20(6):886-905. | Not general population |
| 94 | Botham AD. Suicide: Exploring the Relationship Between Modality and Population Demographic (San Mateo County, CA, USA 2007-2018): A Population-Based Study. Journal of forensic sciences.64(6):1750-60. | No extractable data |
| 95 | Botsis A, Kapsali A, Vaidakis N, Stefanis C. Suicidal Behaviour in Greece. Schmidtke, Armin [Ed]; Bille-Brahe, Unni [Ed]; DeLeo, Diego [Ed]; Kerkhof, Ad [Ed] (2004) Suicidal behaviour in Europe: Results from the WHO/EURO Multicentre Study on Suicidal Behaviour (pp 219-222) xii, 288 pp Ashland, OH, US: Hogrefe & Huber Publishers; US. 2004:219-22. | Grey literature |
| 96 | Bowden M, McCoy A, Reavley N. Suicidality and suicide prevention in culturally and linguistically diverse (cald) communities: A systematic review. International Journal of Mental Health.No Pagination Specified. | No extractable data |
| 97 | Brenburg MA. Prescription Opioids Are Associated With Population Mortality in US Deep South Middle-Age Non-hispanic Whites: An Ecological Time Series Study. Frontiers in Public Health. 2019;7:252. | No ethnicity data |
| 98 | Braden JB, Edlund MJ, Sullivan MD. Suicide Deaths With Opioid Poisoning in the United States: 1999-2014. American journal of public health.107(3):421-6. | No extractable data |
| 99 | Bready JC, Bready RJ, Chute DJ. A Ten-year Study of Suicides from a Rural/Suburban County. Journal of forensic sciences.62(4):911-4. | No ethnicity data |
| 100 | Brenes F. Hispanics, Mental Health, and Suicide: Brief Report. Hispanic health care international : the official journal of the National Association of Hispanic Nurses.17(3):133-6. | No data on suicide |
| 101 | Bridge JA, Barbe RP, Brent DA. Recent Trends in Suicide Among U.S. Adolescent Males, 1992-2001. Psychiatric Services.56(5):522. | No extractable data |
| 102 | Bridge JA, Goldstein TR, Brent DA. Adolescent suicide and suicidal behavior. Journal of Child Psychology & Psychiatry & Allied Disciplines. 2006;47(3):372-94. | No ethnicity data |
| 103 | Bridge JA, Horowitz LM, Fontanella CA, Sheftall AH, Greenhouse J, Kelleher KJ, et al. Age-related racial disparity in suicide rates among US youths from 2001 through 2015. JAMA Pediatrics.172(7):697-9. | No ethnicity data |
| 104 | Brinkley-Rubinstein L, Sivaraman J, Rosen DL, Cloud DH, Junker G, Proescholdbell S, et al. Association of Restrictive Housing during Incarceration with Mortality after Release. JAMA Network Open. 2019. | Not general population |
| 105 | Brunstein Klomek A, Nakash O, Goldberger N, Haklai Z, Geraisy N, Yatzkar U, et al. Completed suicide and suicide attempts in the Arab population in Israel. Social psychiatry and psychiatric epidemiology.51(6):869-76. | No ethnicity data |
| 106 | Buchanan KL, Flowers K, Salami T, Walker RL. Racial and ethnic differences. Lamis, Dorian A [Ed]; Lester, David [Ed] (2011) Understanding and preventing college student suicide (pp 65-79) xix, 339 pp Springfield, IL, US: Charles C Thomas Publisher; US. 2011:65-79. | No extractable data |
| 107 | Buescher PA. A review of available data on the health of the Latino population in North Carolina. North Carolina medical journal. 2003;64(3):97-105. | No extractable data |
| 108 | Bullock MJ, Diniz D. Suffocation using plastic bags: A retrospective study of suicides in Ontario, Canada. Journal of Forensic Sciences.45(3):608-13. | No extractable data |
| 109 | Burgess AW, Sekula LK, Carretta CM. Homicide-suicide and duty to warn. Psychodynamic psychiatry.43(1):67-90. | Not general population |
| 110 | Burns KM, Cottengim C, Dykstra H, Faulkner M, Erck Lambert AB, MacLeod H, et al. Epidemiology of Sudden Death in a Population-Based Study of Infants and Children. Journal of Pediatrics: X.2. | No ethnicity data |
| 111 | Burrows S, Laflamme L. Living circumstances of suicide mortality in a South African city: An ecological study of differences across race groups and sexes. Suicide and Life-Threatening Behavior.35(5):592-603. | No extractable data |
| 112 | Burrows S, Laflamme L. Pattern analysis of suicide mortality surveillance data in urban South Africa. Suicide and Life-Threatening Behavior.38(2):209-20. | No extractable data |
| 113 | Burrows S, Laflamme L. Assessment of accuracy of suicide mortality surveillance data in South Africa: Investigation in an urban setting. Crisis. 2007;28(2):74-81. | No extractable data |
| 114 | Burrows S, Laflamme L. Suicide among urban South African adolescents. International Journal of Adolescent Medicine & Health. 2008;20(4):519-28. | No extractable data |
| 115 | Burrows S, Vaez M, Butchart A, Laflamme L. The share of suicide in injury deaths in the South African context: Sociodemographic distribution. Public Health.117(1):3-10. | No ethnicity data |
| 116 | Burrows S, Vaez M, Laflamme L. Sex-specific suicide mortality in the South African urban context: The role of age, race, and geographical location. Scandinavian Journal of Public Health. 2007;35(2):133-9. | No extractable data |
| 117 | Bursztein Lipsicas C, Makinen IH, Apter A, De Leo D, Kerkhof A, Lonnqvist J, et al. Attempted suicide among immigrants in European countries: an international perspective. Social psychiatry and psychiatric epidemiology.47(2):241-51. | No data on suicide |
| 118 | Byard RW, Austin AE, Van Den Heuvel C. Characteristics of asphyxial deaths in adolescence. Journal of Forensic and Legal Medicine.18(3):107-9. | No extractable data |
| 119 | Cabrera-Mendoza B, Fresno C, Monroy-Jaramillo N, Fries GR, Walss-Bass C, Glahn DC, et al. Sex differences in brain gene expression among suicide completers. Journal of Affective Disorders.267:67-77. | No ethnicity data |
| 120 | Caetano R, Kaplan M, Huguet N, Conner K, McFarl, B, et al. Ethnicity, drinking, and associated problems amongsuicide decedents: National violent death reporting system (NVDRS) 2003-2009. Alcoholism: Clinical and Experimental Research.37:296A. | No extractable data |
| 121 | Caetano R, Kaplan MS, Huguet N, Conner K, McFarl, BH, et al. Precipitating Circumstances of Suicide and Alcohol Intoxication Among U.S. Ethnic Groups. Alcoholism: Clinical and Experimental Research.39(8):1510-7. | No extractable data |
| 122 | Caetano R, Kaplan MS, Huguet N, McFarl, BH, Conner K, et al. Acute Alcohol Intoxication and Suicide Among United States Ethnic/Racial Groups: Findings from the National Violent Death Reporting System. Alcoholism: Clinical and Experimental Research.37(5):839-46. | No extractable data |
| 123 | Caetano R, Kaplan MS, Kerr W, McFarl, BH, Giesbrecht N, et al. Suicide, Alcohol Intoxication, and Age Among Whites and American Indians/Alaskan Natives. Alcoholism: Clinical and Experimental Research.44(2):492-500. | No extractable data |
| 124 | Calabria B, Doran CM, Vos T, Shakeshaft AP, Hall W. Epidemiology of alcohol-related burden of disease among Indigenous Australians. Australian and New Zealand journal of public health.34:S47-51. | No extractable data |
| 125 | Campanelli C, Gilson T. Murder-suicide in New Hampshire, 1995-2000. American Journal of Forensic Medicine and Pathology.23(3):248-51. | Not general population |
| 126 | Campbell A, Balaratnasingam S, McHugh C, Janca A, Chapman M. Alarming increase of suicide in a remote Indigenous Australian population: an audit of data from 2005 to 2014. World Psychiatry.15(3):296-7. | No ethnicity data |
| 127 | Campbell A, Chapman M, McHugh C, Sng A, Balaratnasingam S. Rising Indigenous suicide rates in Kimberley and implications for suicide prevention. Australasian Psychiatry. 2016;24(6):561-4. | No extractable data |
| 128 | Cantor C, Neulinger K. The epidemiology of suicide and attempted suicide among young Australians. Australian and New Zealand Journal of Psychiatry.34(3):370-87. | No extractable data |
| 129 | Carpenter B, Bond C, Tait G, Wilson M, White K. Who Leaves Suicide Notes? An Exploration of Victim Characteristics and Suicide Method of Completed Suicides in Queensland. Archives of suicide research : official journal of the International Academy for Suicide Research. 2016;20(2):176-90. | No ethnicity data |
| 130 | Case A, Deaton A. Rising morbidity and mortality in midlife among white non-Hispanic Americans in the 21st century. Proceedings of the National Academy of Sciences of the United States of America.112(49):15078-83. | No extractable data |
| 131 | Castellanos D, Kosoy JE, Ayllon KD, Acuna J. Presence of Alcohol and Drugs in Hispanic Versus Non-Hispanic Youth Suicide Victims in Miami-Dade County, Florida. Journal of immigrant and minority health.18(5):1024-31. | No extractable data |
| 132 | Castelpietra G, Egidi L, Caneva M, Gambino S, Feresin T, Mariotto A, et al. Suicide and suicides attempts in Italian prison epidemiological findings from the "Triveneto" area, 2010-2016. International Journal of Law and Psychiatry.61:6-12. | Not general population |
| 133 | Castle K, Duberstein PR, Meldrum S, Conner KR, Conwell Y. Risk Factors for Suicide in Blacks and Whites: An Analysis of Data from the 1993 National Mortality Followback Survey. American Journal of Psychiatry.161(3):452-8. | No extractable data |
| 134 | Cavalcante FG, Minayo MC, Mangas RM. [Different aspects of depression in suicide among the elderly]. Ciencia & Saude Coletiva. 2013;18(10):2985-94. | No data on suicide |
| 135 | Centers for Disease C, Prevention. Suicide trends and characteristics among persons in the Guarani Kaiowa and Nandeva communities--Mato Grosso do Sul, Brazil, 2000-2005. MMWR - Morbidity & Mortality Weekly Report. 2007;56(1):7-9. | Grey literature |
| 136 | Centers for Disease C, Prevention. Suicide and suicidal ideation among Bhutanese refugees--United States, 2009-2012. MMWR - Morbidity & Mortality Weekly Report. 2013;62(26):533-6. | Grey literature |
| 137 | Cerel J, Singleton MD, Brown MM, Brown SV, Bush HM, Brancado CJ. Emergency Department Visits Prior to Suicide and Homicide: Linking Statewide Surveillance Systems. Crisis. 2016;37(1):5-12. | Not general population |
| 138 | Ch, ramouleeswaran S, Yalsangi M. Epidemiology of completed suicides in a remote tribal population in south india. Indian Journal of Psychiatry.60:S164. | No extractable data |
| 139 | Chachamovich E, Kirmayer LJ, Haggarty JM, Cargo M, McCormick R, Turecki G. Suicide among Inuit: Results from a large, epidemiologically representative follow-back study in Nunavut. Canadian Journal of Psychiatry.60(6):268-75. | No ethnicity data |
| 140 | Chang MH, Moonesinghe R, Athar HM, Truman BI. Trends in Disparity by Sex and Race/Ethnicity for the Leading Causes of Death in the United States-1999-2010. Journal of public health management and practice: JPHMP.22:S13-S24. | No extractable data |
| 141 | Charlier P, Malaurie J, Wasserman D, Carli V, Sarchiapone M, Dagenais-Everell C, et al. The EPA guidance on suicide treatment and prevention needs to be adjusted to fight the epidemics of suicide at the North Pole area and other autochthonous communities. European Psychiatry.41:129-31. | No extractable data |
| 142 | Chavan BS, Singh GP, Kaur J, Kochar R. Psychological autopsy of 101 suicide cases from northwest region of India. Indian Journal of Psychiatry. 2008;50(1):34-8. | No extractable data |
| 143 | Cheung YT, Spittal MJ, Williamson MK, Tung SJ, Pirkis J. Application of scan statistics to detect suicide clusters in Australia. PLoS ONE [Electronic Resource]. 2013;8(1):e54168. | No extractable data |
| 144 | Cheung YTD, Spittal MJ, Pirkis J, Yip PSF. Spatial analysis of suicide mortality in Australia: Investigation of metropolitan-rural-remote differentials of suicide risk across states/territories. Social Science and Medicine.75(8):1460-8. | No ethnicity data |
| 145 | Chia B-H, Chia A, Yee NW, Choo TB. Suicide trends in Singapore: 1955-2004. Archives of Suicide Research.14(3):276-83. | No extractable data |
| 146 | Chiurliza B, Michaels MS, Joiner TE. Acquired capability for suicide among individuals with American Indian/Alaska Native backgrounds within the military. American Indian and Alaska native mental health research (Online). 2016;23(4):1-15. | Not general population |
| 147 | Choi J, Zarkar S, Tatum J, Rice TR. Asian International Students and Suicide in the United States. Asian Journal of Psychiatry.52. | No ethnicity data |
| 148 | Choi NG, Dinitto DM, Marti CN, Choi BY. Poisoning deaths among late-middle aged and older adults: Comparison between suicides and deaths of undetermined intent. International Psychogeriatrics.31(8):1159-69. | No extractable data |
| 149 | Choi NG, DiNitto DM, Sagna AO, Marti C. Older women who died by suicide: Suicide means, sociodemographic and psychiatric risk factors, and other precipitating circumstances. International Psychogeriatrics.30(10):1531-40. | No extractable data |
| 150 | Choi NG, Marti CN, DiNitto DM, Choi BY. Suicides and Deaths of Undetermined Intent by Poisoning: Reexamination of Classification Differences by Race/Ethnicity and State. Archives of suicide research: official journal of the International Academy for Suicide Research.1-28. | No extractable data |
| 151 | Chuang H-L, Huang W-C. Re-examination of the suicide rates in Taiwan. Social Indicators Research.83(3):465-85. | No ethnicity data |
| 152 | Chung KH, Lee HC, Chen SF, Lin HC. The ten-year trend in suicide methods: Evidence from an asian population. Canadian Journal of Public Health.99(5):406-10. | No ethnicity data |
| 153 | Chung SH, Kwon S, Mason M, Slater M. Demographics and characteristics of violent deaths in children. Pediatrics Conference: National Conference on Education. 2016;141(1). | Grey literature |
| 154 | Cochran J, Geltman PL, Ellis H, Brown C, Anderton S, Montour J, et al. Suicide and suicidal ideation among Bhutanese refugees -United States, 2009-2012. Morbidity and Mortality Weekly Report.62(26):533-6. | Grey literature |
| 155 | Cohen J. Safe in our hands?: A study of suicide and self-harm in asylum seekers. Journal of Forensic and Legal Medicine.15(4):235-44. | No ethnicity data |
| 156 | Coloma C, Hoffman JS, Crosby A. Suicide among Guarani Kaiowa and Nandeva youth in Mato Grosso do Sul, Brazil. Archives of Suicide Research.10(2):191-207. | No extractable data |
| 157 | Colombo-Souza P, Tranchitella FB, Ribeiro AP, Juliano Y, Novo NF. Suicide mortality in the city of Sao Paulo: epidemiological characteristics and their social factors in a temporal trend between 2000 and 2017. Retrospective study. Sao Paulo medical journal = Revista paulista de medicina. 2020;22. | No extractable data |
| 158 | Conner KR, Cox C, Duberstein PR, Tian L, Nisbet PA, Conwell Y. Violence, alcohol, and completed suicide: A case-control study. American Journal of Psychiatry. 2001;158(10):1701-5. | No ethnicity data |
| 159 | Conner KR, Huguet N, Caetano R, Giesbrecht N, McFarl, BH, et al. Acute use of alcohol and methods of suicide in a US national sample. American journal of public health.104(1):171-8. | No extractable data |
| 160 | Cook A, Osler T, Hosmer D, Glance L, Rogers F, Gross B, et al. Gunshot wounds resulting in hospitalization in the United States: 2004-2013. Injury.48(3):621-7. | No ethnicity data |
| 161 | Cook TB. Recent court involvement and risk of suicide: A population-based study utilizing a comprehensive criminal justice database. Dissertation Abstracts International: Section B: The Sciences and Engineering. 2012;73(6):3494. | Grey literature |
| 162 | Cortes E, Cubano A, Lewis JE, Castellanos D. Antidepressants at Autopsy in Hispanic Suicidal Youth in Miami-Dade County, Florida. Journal of Forensic Sciences.56(1):155-60. | No extractable data |
| 163 | Coskun M, Zoroglu S, Ghaziuddin N. Suicide Rates among Turkish and American Youth: A Cross-Cultural Comparison. Archives of Suicide Research.16(1):59-72. | No ethnicity data |
| 164 | Cousins S. Suicide in Indigenous Australians: a "catastrophic crisis". Lancet (London, England).389(10066):242. | No ethnicity data |
| 165 | Couto C, Barreto S, ra, Neto MLR. Suicide in Black children. International Journal of Social Psychiatry.64(5):506-8. | No ethnicity data |
| 166 | Crawford MJ, Kuforiji B, Ghosh P. The impact of social context on socio-demographic risk factors for suicide: A synthesis of data from case-control studies. Journal of Epidemiology and Community Health.64(6):530-4. | No ethnicity data |
| 167 | Crnek-Georgeson KT, Wilson LA, Page A. Factors influencing suicide in older rural males: a review of Australian studies. Rural and remote health.17(4):4020. | No ethnicity data |
| 168 | Crosby AE, Ortega L, Stevens MR, Centers for Disease C, Prevention. Suicides - United States, 2005-2009. Morbidity and mortality weekly report.179-83. | Grey literature |
| 169 | Crowder MK, Kemmelmeier M. New insights on cultural patterns of suicide in the United States: The role of honor culture. Cross-Cultural Research: The Journal of Comparative Social Science.51(5):521-48. | No ethnicity data |
| 170 | Cubbin C, LeClere FB, Smith GS. Socioeconomic status and the occurrence of fatal and nonfatal injury in the United States. American Journal of Public Health.90(1):70-7. | No data on suicide |
| 171 | Cutcliffe JR. Toward an understanding of suicide in First-Nation Canadians. Crisis. 2005;26(3):141-5. | No extractable data |
| 172 | Cutlip AC, Bankston WB, Lee MR. Civic community and nonmetropolitan white suicide. Archives of Suicide Research.14(3):261-5. | No ethnicity data |
| 173 | Cutright P, Fernquist RM. Effects of societal intergration, period, region, and culture of suicide on male age-specific suicide rates: 20 developed countries, 1955-1989. Social Science Research. 2000;29(1):148-72. | No ethnicity data |
| 174 | Cutright P, Fernquist RM. The culture of suicide through societal integration and religion: 1996-1998 gender-specific suicide rates in 50 American states. Archives of Suicide Research. 2004;8(3):271-85. | No extractable data |
| 175 | Dabbagh N. Behind the statistics: the ethnography of suicide in Palestine. Culture, Medicine & Psychiatry. 2012;36(2):286-305. | No ethnicity data |
| 176 | dal ND. A qualitative comparison of investigatory summaries of suicide and follow-back interviews. Dissertation Abstracts International: Section B: The Sciences and Engineering. 2012;73(3):1477. | No ethnicity data |
| 177 | Dare AJ, Irving H, Guerrero-Lopez CM, Watson LK, Kolpak P, Reynales Shigematsu LM, et al. Geospatial, racial, and educational variation in firearm mortality in the USA, Mexico, Brazil, and Colombia, 1990-2015: a comparative analysis of vital statistics data. The Lancet Public Health.4(6):e281-e90. | No extractable data |
| 178 | Das-Munshi J, Dewey M, Becares L. Ethnic density associations for mental health: Systematic Review and meta-analysis of international studies. European Psychiatry.48:S428. | Grey literature |
| 179 | Davanzo F, Settimi L, Condo M, Marcello I, Sesana F, Zoppi F, et al. Methanol poisonings in Italy: 2004-2008. Clinical Toxicology.47:478. | No data on suicide |
| 180 | Davanzo F, Settimi L, Condo M, Marcello I, Zoppi F, Binetti R. A cluster of methanol-related poisonings in Sicily: case characterization and identification of unexpected sources of exposure. [Italian]. Epidemiologia e prevenzione. 2009;33(3):104-12. | No data on suicide |
| 181 | David-Ferdon C, Crosby AE, Caine ED, Hindman J, Reed J, Isk, et al. CDC Grand Rounds: Preventing Suicide Through a Comprehensive Public Health Approach. Mmwr. (34):894-7. | No ethnicity data |
| 182 | de la Fuente JR. Impacts of globalization on mental health. Gaceta Medica de Mexico.148(6):586-90. | No extractable data |
| 183 | De Leo D. Australia revises its mortality data on suicide. Crisis: The Journal of Crisis Intervention and Suicide Prevention. 2010;31(4):169-73. | No ethnicity data |
| 184 | De Leo D, Evans R. International suicide rates and prevention strategies. (2004) International suicide rates and prevention strategies xvii, 150 pp Ashland, OH, US: Hogrefe & Huber Publishers; US. 2004. | Grey literature |
| 185 | De Leo D, Milner A, Sveticic J. Mental disorders and communication of intent to die in indigenous suicide cases, Queensland, Australia. Suicide & life-threatening behavior.42(2):136-46. | No extractable data |
| 186 | De Noli MF. [Suicide among immigrants increases. It increased 10.3 percent in 2012, among Swedish-born 2.8 per cent]. Lakartidningen. 2014;111(8):326. | No extractable data |
| 187 | De Oliveira CS, Lotufo Neto F. Suicide among indigenous people: A brazilian statistical view. [Portuguese]. Revista de Psiquiatria Clinica. 2003;30(1):4-10. | No data on suicide |
| 188 | de Souza MLP, Orellana JDY. Suicide mortality in SaO Gabriel da Cachoeira, a predominantly indigenous Brazilian municipality. Revista Brasileira de Psiquiatria.34(1):34-7. | No ethnicity data |
| 189 | de Souza MLP, Orellana JDY. Suicide among the indigenous people in Brazil: A hidden public health issue. Revista Brasileira de Psiquiatria.34(4):489-90. | No extractable data |
| 190 | de Souza MLP, Orellana JDY. Inequalities in suicide mortality between indigenous and non-indigenous people in the State of Amazonas, Brazil. Jornal Brasileiro de Psiquiatria. 2013;62(4):245-52. | No extractable data |
| 191 | de Souza RSB, de Oliveira JC, Alvares-Teodoro J, Teodoro MLM. [Suicide and indigenous populations in Brazil: systematic reviewEl suicidio y los pueblos indigenas brasilenos: revision sistematica]. Pan American Journal of Public Health. 2020;44:e58. | No extractable data |
| 192 | Dennis KN. Black political and socioeconomic status attainment and the direction of lethal violence: Comparing the suicide of young Black and White males in U.S. counties. Dissertation Abstracts International Section A: Humanities and Social Sciences. 2011;71(12):4572. | Grey literature |
| 193 | Dennis M, Shah A, Lindesay J. Methods of elderly suicides in England and Wales by country of birth groupings. International Journal of Geriatric Psychiatry.24(11):1311-3. | No extractable data |
| 194 | Derek Cheung YT, Spittal MJ, Williamson MK, Tung SJ, Pirkis J. Predictors of suicides occurring within suicide clusters in Australia, 2004-2008. Social Science and Medicine. 2014;118:135-42. | No extractable data |
| 195 | Dervic K, Amiri L, Niederkrotenthaler T, Yousef S, Salem MO, Voracek M, et al. Suicide rates in the national and expatriate population in Dubai, United Arab Emirates. The International journal of social psychiatry.58(6):652-6. | No ethnicity data |
| 196 | Di Canio PT. Completed suicide in adolescence: A psychological autopsy study comparing African American and Caucasian males. Dissertation Abstracts International: Section B: The Sciences and Engineering.61(4):2197. | Not general population |
| 197 | Dickson JM, Cruise K, McCall CA, Taylor PJ. A systematic review of the antecedents and prevalence of suicide, self-harm and suicide ideation in Australian aboriginal and torres strait islander youth. International Journal of Environmental Research and Public Health. 2019;16(17). | No ethnicity data |
| 198 | Dobscha SK, Denneson LM, Kovas AE, Teo A, Forsberg CW, Kaplan MS, et al. Correlates of Suicide Among Veterans Treated in Primary Care: Case-Control Study of a Nationally Representative Sample. Journal of General Internal Medicine. 2014;29(4):853-60. | Not general population |
| 199 | Doddakashi V, Wilcox RE, Hauser LA. Female suicides in major Texas cities, 1994 through 1998. Texas medicine.99(7):50-8. | No extractable data |
| 200 | Dong XQ, Chang ES, Zeng P, Simon MA. Suicide in the global Chinese aging population: A review of risk and protective factors, consequences, and interventions. Aging and Disease. 2015;6(2):121-30. | No ethnicity data |
| 201 | dos Santos Teles A, de Araujo Oliveira RF, Bahia Coelho TC, Vinhas Ribeiro G, Lima Mendes WM, Prates Santos PN. Role of drugs in intoxication caused by chemicals in a city in Bahia (Brazil), in the period 2007 to 2010. Revista de Ciencias Farmaceuticas Basica e Aplicada. 2013;34(2):281-8. | No extractable data |
| 202 | Dranger EA, Hoffman JM. Has There Been Progress in Reducing Mortality among Wisconsin Adults Ages 25 to 44? Wisconsin Medical Journal. 2003;102(8):42-6. | No extractable data |
| 203 | Duarte-Velez YM, Bernal G. Suicide risk in Latino and Latina adolescents. Leong, Frederick T L [Ed]; Leach, Mark M [Ed] (2008) Suicide among racial and ethnic minority groups: Theory, research, and practice (pp 81-115) xviii, 334 pp New York, NY, US: Routledge/Taylor & Francis Group; US. 2008:81-115. | No ethnicity data |
| 204 | Dudarev AA, Chupakhin VS, Odl, JO. Health and society in Chukotka: an overview. International journal of circumpolar health. 2013;72:20469. | No ethnicity data |
| 205 | Duduyemi BM, Ojo BA. Coroner's autopsies in Nigeria capital city of Abuja: A review of 65 consecutive cases. Indian Journal of Forensic Medicine and Toxicology.8(1):53-7. | No ethnicity data |
| 206 | Dumitru MM, Papari AC, Sava NI, Papari A. Socio-demographic characteristics of suicides in Romania. European Psychiatry.30:394. | No ethnicity data |
| 207 | Duthe G, Hazard A, Kensey A, Shon JL. Suicide among male prisoners in France: a prospective population-based study. Forensic Science International. 2013;233(1):273-7. | No ethnicity data |
| 208 | Edwards N, Alaghehb, an R, MacDonald D, Sikdar K, Collins K, et al. Suicide in Newfoundland and Labrador: A linkage study using medical examiner and vital statistics data. Canadian Journal of Psychiatry.53(4):252-9. | No ethnicity data |
| 209 | Eggertson L. Nunavut youth saturated in the realities of suicide. CMAJ : Canadian Medical Association journal = journal de l'Association medicale canadienne.185(11):943-4. | No ethnicity data |
| 210 | Eggertson L. Risk of suicide 40 times higher for Inuit boys. CMAJ : Canadian Medical Association journal = journal de l'Association medicale canadienne.185(15):E701-2. | No ethnicity data |
| 211 | Eggertson L. Aboriginal youth suicide rises in Northern Ontario. CMAJ : Canadian Medical Association journal = journal de l'Association medicale canadienne.187(11):E335-E6. | No ethnicity data |
| 212 | Eisenberg-Guyot J, Mooney SJ, Hagopian A, Barrington WE, Hajat A. Solidarity and disparity: Declining labor union density and changing racial and educational mortality inequities in the United States. American journal of industrial medicine.63(3):218-31. | No ethnicity data |
| 213 | El-Sayed AM, Tracy M, Galea S. Suicide risk among arab-americans: The roles of gender and place. American Journal of Epidemiology.171:S15. | No extractable data |
| 214 | El-Sayed AM, Tracy M, Galea S. Life expectancy and mortality among Arab Americans. American Journal of Epidemiology.171:S14. | No extractable data |
| 215 | El-Sayed AM, Tracy M, Scarborough P, Galea S. Suicide among Arab-Americans. PLoS ONE. 2011;6(2). | No extractable data |
| 216 | Ellsaer G, Albrecht M. Circumstances of injury in childhood and adolescence. Data and epidemiology. [German]. Bundesgesundheitsblatt - Gesundheitsforschung - Gesundheitsschutz.53(10):1104-12. | No extractable data |
| 217 | Else IR, Andrade NN. Examining suicide and suicide-related behaviors among indigenous Pacific Islanders in the United States: A historical perspective. Leong, Frederick T L [Ed]; Leach, Mark M [Ed] (2008) Suicide among racial and ethnic minority groups: Theory, research, and practice (pp 143-172) xviii, 334 pp New York, NY, US: Routledge/Taylor & Francis Group; US. 2008:143-72. | No data on suicide |
| 218 | Else IRN, Andrade NN, Nahulu LB. Suicide and suicidal-related behaviors among indigenous pacific islanders in the United States. Death Studies.31(5):479-501. | No data on suicide |
| 219 | Engelbrecht C, Blumenthal R, Morris NK, Saayman G. Suicide in Pretoria: A retrospective review, 2007 - 2010. South African Medical Journal.107(8):715-8. | No extractable data |
| 220 | Ertl A, Sheats KJ, Petrosky E, Betz CJ, Yuan K, Fowler KA. Surveillance for Violent Deaths - National Violent Death Reporting System, 32 States, 2016. Morbidity and mortality weekly report.1-36. | Grey literature |
| 221 | Fadel SA, Boschi-Pinto C, Yu S, Reynales-Shigematsu LM, Menon GR, Newcombe L, et al. Trends in cause-specific mortality among children aged 5-14 years from 2005 to 2016 in India, China, Brazil, and Mexico: an analysis of nationally representative mortality studies. The Lancet.393(10176):1119-27. | No ethnicity data |
| 222 | Faria NM, Victora CG, Meneghel SN, de Carvalho LA, Falk JW. Suicide rates in the State of Rio Grande do Sul, Brazil: association with socioeconomic, cultural, and agricultural factors. Cadernos de Saude Publica. 2006;22(12):2611-21. | No ethnicity data |
| 223 | Farrell S, Kapur N, While D, Appleby L, Windfuhr K. Suicide in a National Student Mental Health Patient Population, 1997-2012. Crisis.38(2):82-8. | Not general population |
| 224 | Farrugia LA, Rhyee SH, Campleman SL, Ruha AM, Weig, T, et al. The Toxicology Investigators Consortium Case Registry-the 2015 Experience. Journal of Medical Toxicology.12(3):224-47. | No data on suicide |
| 225 | Fathelrahman AI, Ab Rahman AF, Mohd Zain Z. Self-poisoning by drugs and chemicals: variations in demographics, associated factors and final outcomes. General Hospital Psychiatry.30(5):467-70. | Not general population |
| 226 | Fazel S, Grann M, Kling B, Hawton K. Prison suicide in 12 countries: an ecological study of 861 suicides during 2003-2007. Social Psychiatry & Psychiatric Epidemiology. 2011;46(3):191-5. | No ethnicity data |
| 227 | Ferguson M, Baker A, Young S, Procter N. Understanding Suicide among Aboriginal Communities. Australian nursing & midwifery journal.23(8):36. | No ethnicity data |
| 228 | Fernquist RM. Education, race/ethnicity, age, sex, and suicide: Individual-level data in the United States, 1991-1994. Current Research in Social Psychology.6(18):No Pagination Specified. | No extractable data |
| 229 | Fernquist RM. Suicide rates and status integration in America. Archives of suicide research : official journal of the International Academy for Suicide Research. 2009;13(3):291-6. | No extractable data |
| 230 | Ferry J. No easy answer to high native suicide rates. Lancet.355(9207):906. | No ethnicity data |
| 231 | Firebaugh G, Acciai F, Noah AJ, Prather C, Nau C. Why lifespans are more variable among blacks than among whites in the United States. Demography.51(6):2025-45. | No extractable data |
| 232 | Fleegler EW, Lee LK, Monuteaux MC, Hemenway D, Mannix R. Firearm legislation and firearm-related fatalities in the United States. JAMA Internal Medicine.173(9):732-40. | No ethnicity data |
| 233 | Flisher AJ, Liang H, Laubscher R, Lombard CF. Suicide trends in South Africa, 1968-90. Scandinavian Journal of Public Health. 2004;32(6):411-8. | No extractable data |
| 234 | Fontanella CA, Warner LA, Steelesmith D, Bridge JA, Sweeney HA, Campo JV. Clinical Profiles and Health Services Patterns of Medicaid-Enrolled Youths Who Died by Suicide. JAMA Pediatrics.174(5):470-7. | No ethnicity data |
| 235 | Forster PM, Kuruleca SC, Auxier C. A note on recent trends in suicide in Fiji. Journal of Pacific Rim Psychology.1(1):1-4. | No ethnicity data |
| 236 | Fowler KA, Dahlberg LL, Haileyesus T, Annest JL. Firearm injuries in the United States. Preventive Medicine.79:5-14. | No extractable data |
| 237 | Fowler KA, Jack SPD, Lyons BH, Betz CJ, Petrosky E. Surveillance for Violent Deaths -National Violent Death Reporting System, 18 States, 2014. Morbidity and mortality weekly report.1-36. | Grey literature |
| 238 | Frank E, Biola H, Burnett CA. Mortality rates and causes among U.S. physicians. American Journal of Preventive Medicine.19(3):155-9. | No ethnicity data |
| 239 | Fried LE, Williams S, Cabral H, Hacker K. Differences in Risk Factors for Suicide Attempts Among 9th and 11th Grade Youth: A Longitudinal Perspective. Journal of School Nursing.29(2):113-22. | No data on suicide |
| 240 | Friedman MJ. Risk factors for suicides among army personnel. JAMA - Journal of the American Medical Association.313(11):1154-5. | Not general population |
| 241 | Fu H, Dai WW, Jia PL, Huang K, Meng H, Yang QK, et al. Analysis of 124 Suicide Cases in Wuhua District in Kunming. [Chinese]. Fa yi xue za zhi.34(3):253-6. | No extractable data |
| 242 | Gabbidon SL, Peterson SA. Living while Black: A State-Level Analysis of the Influence of Select Social Stressors on the Quality of Life Among Black Americans. Journal of Black Studies.37(1):83-102. | Not general population |
| 243 | Gallagher LM, Kappatos D, Tisch C, Ellis PM. Suicide by poisoning in New Zealand-a toxicological analysis. New Zealand Medical Journal.125(1362):15-25. | No ethnicity data |
| 244 | Galta K, Olsen SL, Wik G. Murder followed by suicide: Norwegian data and international literature. Nordic Journal of Psychiatry. 2010;64(6):397-401. | No ethnicity data |
| 245 | Gao X, Jin Y, Wang Y, Ye PP, Duan LL. [Analysis on suicide mortality and self-inflicted injury/suicide hospital cases in China from 2006 to 2016]. Chung-Hua Yu Fang i Hsueh Tsa Chih [Chinese Journal of Preventive Medicine]. 2019;53(9):885-90. | No extractable data |
| 246 | Garlich FM, Alsop JA, Anderson DL, Geller RJ, Kalugdan TT, Roberts DJ, et al. Poisoning and suicide by cyanide jewelry cleaner in the US Hmong community: A case series. Clinical Toxicology.50(2):136-40. | No ethnicity data |
| 247 | Garlow SJ. Age, gender, and ethnicity differences in patterns of cocaine and ethanol use preceding suicide. American Journal of Psychiatry. 2002;159(4):615-9. | Not general population |
| 248 | Garssen MJ, Hoogenboezem J, Kerkhof AJ. [Suicide among migrant populations and native Dutch in The Netherlands]. Nederlands Tijdschrift voor Geneeskunde. 2006;150(39):2143-9. | No extractable data |
| 249 | Gerstner RMF, Soriano I, Sanhueza A, Caffe S, Kestel D. [Epidemiology of suicide among adolescents and young adults in EcuadorEpidemiologia do suicidio em adolescentes e jovens no Equador]. Pan American Journal of Public Health. 2018;42:e100. | No extractable data |
| 250 | Gertner AK, Rotter JS, Shafer PR. Association Between State Minimum Wages and Suicide Rates in the U.S. American Journal of Preventive Medicine.56(5):648-54. | No ethnicity data |
| 251 | Gibbs JT. Ethnic Variations in Youth Suicide. Rutter, Michael; Tienda, Marta (2005) Ethnicity and causal mechanisms (pp 262-280) xx, 378 pp New York, NY, US: Cambridge University Press; US. 2005:262-80. | Grey literature |
| 252 | Giesbrecht N, Huguet N, Caetano R, Conner KR, Kaplan MS, McFarl, et al. Impact of on-and off-premise alcohol outlet density on alcohol-related suicide: A multi-state analysis. Alcoholism: Clinical and Experimental Research.38:145A. | No extractable data |
| 253 | Giesbrecht N, Huguet N, Ogden L, Kaplan MS, McFarl, BH, et al. Acute alcohol use among suicide decedents in 14 US states: impacts of off-premise and on-premise alcohol outlet density. Addiction (Abingdon, England).110(2):300-7. | No ethnicity data |
| 254 | Gill JR, Lenz KA, Amolat MJ. Gunshot fatalities in children and adolescents in New York City. Journal of Forensic Sciences.48(4):832-5. | Not general population |
| 255 | Giotakos O, Karabelas D, Kafkas A. [Financial crisis and mental health in Greece]. Psychiatriki. 2011;22(2):109-19. | No extractable data |
| 256 | Giupponi G, Conca A, Schmidt E, Hinterhuber H, Pompili M, Pycha R. Suicide in ethnic and cultural minorities - A research on literature. [German]. Neuropsychiatrie. 2011;25(2):93-102. | No extractable data |
| 257 | Giupponi G, Innamorati M, Baldessarini RJ, De Leo D, de Giovannelli F, Pycha R, et al. Factors associated with suicide: Case-control study in South Tyrol. Comprehensive Psychiatry.80:150-4. | Not general population |
| 258 | Gleich S, Domingo O, Ackermann N, Schwerer M, Graw M, Schopfer J. A post-mortem study of the cause of death and concomitant diseases of refugees in Munich (2014-2015). Rechtsmedizin.28(1):25-32. | Not general population |
| 259 | Glick S, Kerani R, Golden M. Mortality among men who have sex with men seen in std clinic patients: High rates of suicide and low rates of anal cancer death. Sexually Transmitted Infections.87:A156. | No ethnicity data |
| 260 | Gofin R, Avitzour M, Haklai Z, Jellin N. Intentional injuries among the young: Presentation to emergency rooms, hospitalization, and death in Israel. Journal of Adolescent Health. 2000;27(6):434-42. | No ethnicity data |
| 261 | Goncalves REM, Ponce JC, Leyton V. Alcohol use by suicide victims in the city of Sao Paulo, Brazil, 2011-2015. Journal of Forensic & Legal Medicine. 2018;53:68-72. | Not general population |
| 262 | Goodfellow B, Kolves K, Selefen AC, Massain T, Amadeo S, De Leo D. The WHO/START study in New Caledonia: A psychological autopsy case series. Journal of Affective Disorders.262:366-72. | Not general population |
| 263 | Granski M, Keller A, Venters H. Death rates among detained immigrants in the United States. International Journal of Environmental Research and Public Health.12(11):14414-9. | No ethnicity data |
| 264 | Gravseth HM, Mehlum L, Bjerkedal T, Kristensen P. Suicide in young Norwegians in a life course perspective: Population-based cohort study. Journal of Epidemiology and Community Health. 2010;64(5):407-12. | No ethnicity data |
| 265 | Griffith J. Suicide in the army national guard: an empirical inquiry. Suicide & life-threatening behavior.42(1):104-19. | Not general population |
| 266 | Griffith J. Army suicides: "knowns" and an interpretative framework for future directions. Military Psychology.24(5):488-512. | Not general population |
| 267 | Griffith J. A Description of Suicides in the Army National Guard During 2007-2014 and Associated Risk Factors. Suicide & life-threatening behavior.47(3):266-81. | Not general population |
| 268 | Grobler C, Strumpher J, Jacobs R. A retrospective study of completed suicides in the Nelson Mandela Bay Metropolitan Area from 2008 to 2013 - Preliminary results. South African Journal of Psychiatry.21:120-1. | No extractable data |
| 269 | Hagaman AK, Khadka S, Lohani S, Kohrt B. Suicide in Nepal: a modified psychological autopsy investigation from randomly selected police cases between 2013 and 2015. Social psychiatry and psychiatric epidemiology.52(12):1483-94. | No data on suicide |
| 270 | Hagaman AK, Sivilli TI, Ao T, Blanton C, Ellis H, Lopes Cardozo B, et al. An Investigation into Suicides Among Bhutanese Refugees Resettled in the United States Between 2008 and 2011. Journal of immigrant and minority health.18(4):819-27. | No ethnicity data |
| 271 | Haghparast-Bidgoli H, Rinaldi G, Shahnavazi H, Bouraghi H, Kiadaliri AA. Socio-demographic and economics factors associated with suicide mortality in Iran, 2001-2010: application of a decomposition model. International Journal for Equity in Health. 2018;17(1):77. | No ethnicity data |
| 272 | Haglund A, Tidemalm D, Jokinen J, Langstrom N, Lichtenstein P, Fazel S, et al. Suicide after release from prison: A population-based cohort study from Sweden. Journal of Clinical Psychiatry.75(10):1047-53. | No ethnicity data |
| 273 | Hamilton BE, Minino AM, Martin JA, Kochanek KD, Strobino DM, Guyer B. Annual summary of vital statistics: 2005. Pediatrics.119(2):345-60. | Grey literature |
| 274 | Han CS, Oliffe JL, Ogrodniczuk JS. Suicide among East Asians in North America: A scoping review. Journal of Mental Health.22(4):361-71. | No ethnicity data |
| 275 | Hankir A, Bhugra D. Immigrant populations and suicide. Wasserman, Danuta [Ed] (2016) Suicide: An unnecessary death , 2nd ed (pp 161-171) xxxv, 411 pp New York, NY, US: Oxford University Press; US. 2016:161-71. | Grey literature |
| 276 | Haq M, Farheen A, Goli SK. Retrospective analysis of suicidal deaths in north Karnataka. Indian Journal of Forensic Medicine and Toxicology.11(2):36-9. | No ethnicity data |
| 277 | Harris L. The relationship between adolescent suicide and income inequality. Dissertation Abstracts International: Section B: The Sciences and Engineering. 2008;69(4):2268. | Grey literature |
| 278 | Hassamal S, Keyser-Marcus L, Breden EC, Hobron K, Bhattachan A, urangi A. A brief analysis of suicide methods and trends in Virginia from 2003 to 2012. BioMed Research International. 2015. | No extractable data |
| 279 | Hassler S, Sjol, er P, Johansson R, Gronberg H, Damber L. Fatal accidents and suicide among reindeer-herding Sami in Sweden. International journal of circumpolar health. 2004;63:384-8. | No extractable data |
| 280 | Haukka J, Suvisaari J, Sarvimaki M, Martikainen P. The Impact of Forced Migration on Mortality: A Cohort Study of 242,075 Finns from 1939-2010. Epidemiology.28(4):587-93. | No extractable data |
| 281 | Hawton K, van Heeringen K. Suicide. The Lancet. 2009;373(9672):1372-81. | No ethnicity data |
| 282 | Hayati AN, Salina AA, Abdullah AA, Eusni RT, Mansar AH. The pattern of completed suicides seen in Kuala Lumpur General Hospital 1999. The Medical journal of Malaysia.59(2):190-8. | Not general population |
| 283 | Hazlett SB, McCarthy ML, Londner MS, Onyike CU. Epidemiology of Adult Psychiatric Visits to U.S. Emergency Departments. Academic Emergency Medicine.11(2):193-5. | No ethnicity data |
| 284 | He ZX, Lester D. Sex ratio in Chinese suicide. Perceptual and motor skills.95(2):620. | No ethnicity data |
| 285 | He ZX, Lester D. Elderly suicide in China. Psychological Reports. 2001;89(3):675-6. | No ethnicity data |
| 286 | Helaly AM, Ali EF, Zidan EM. The pattern of suicide in the western Kingdom of Saudi Arabia: a retrospective study from 2008 to 2012. The American journal of forensic medicine and pathology.36(1):27-30. | Not general population |
| 287 | Hern, ez AMC, Garcia LF, Macias o, Merino HP, Ramirez L. Epidemiology of suicide in the state of Guanajuato. Salud Mental.27(2):15-26. | No ethnicity data |
| 288 | Hern C, ez AM, Macias Garcia LF, Palatto Merino H, Ramirez L. Epidemiology of suicide in the state of Guanajuato. [Spanish]. Salud Mental.27(2):15-20. | No extractable data |
| 289 | Heron M. Deaths: leading causes for 2008. National vital statistics reports : from the Centers for Disease Control and Prevention, National Center for Health Statistics, National Vital Statistics System.60(6):1-94. | No extractable data |
| 290 | Heron M. Deaths: leading causes for 2010. National vital statistics reports : from the Centers for Disease Control and Prevention, National Center for Health Statistics, National Vital Statistics System.62(6):1-96. | No extractable data |
| 291 | Heron M. Deaths: Leading Causes for 2012. National vital statistics reports : from the Centers for Disease Control and Prevention, National Center for Health Statistics, National Vital Statistics System.64(10):1-93. | No extractable data |
| 292 | Heron M. Deaths: Leading Causes for 2011. National vital statistics reports : from the Centers for Disease Control and Prevention, National Center for Health Statistics, National Vital Statistics System.64(7):1-96. | No extractable data |
| 293 | Heron M. Deaths: Leading Causes for 2013. National vital statistics reports : from the Centers for Disease Control and Prevention, National Center for Health Statistics, National Vital Statistics System.65(2):1-95. | No extractable data |
| 294 | Heron M. Deaths: Leading Causes for 2015. National vital statistics reports : from the Centers for Disease Control and Prevention, National Center for Health Statistics, National Vital Statistics System.66(5):1-76. | No extractable data |
| 295 | Heron M. Deaths: Leading Causes for 2017. National vital statistics reports : from the Centers for Disease Control and Prevention, National Center for Health Statistics, National Vital Statistics System.68(6):1-77. | No extractable data |
| 296 | Heron M. Deaths: Leading causes for 2009. National Vital Statistics Reports. 2012;61(7):1-96. | No extractable data |
| 297 | Hill R, Perkins R, Wexler L. An analysis of hospital visits during the 12 months preceding suicide death in Northern Alaska. Alaska medicine. 2007;49(1):16-21. | Not general population |
| 298 | Hirani K, Cherian S, Mutch R, Payne DN. Identification of health risk behaviours among adolescent refugees resettling in Western Australia. Archives of Disease in Childhood.103(3):240-6. | No ethnicity data |
| 299 | Hjelmel, H, Hawton K, Nordvik H, Bille-Brahe U, De Leo D, et al. Why people engage in parasuicide: A cross-cultural study of intentions. Suicide and Life-Threatening Behavior. 2002;32(4):380-93. | No ethnicity data |
| 300 | Hjern A, Lindblad F, Vinnerljung B. Suicide, psychiatric illness, and social maladjustment in intercountry adoptees in Sweden: A cohort study. Lancet.360(9331):443-8. | Not general population |
| 301 | Holl, er AC, Dalman C. [How migration impacts mental health - epidemiological perspectives]. Lakartidningen. 2020;117(1):14. | No extractable data |
| 302 | Hong J, Knapp M. Impact of macro-level socio-economic factors on rising suicide rates in South Korea: panel-data analysis in East Asia. The journal of mental health policy and economics.17(4):151-62. | No full text |
| 303 | Hooghe M, Vanhoutte B. An ecological study of community-level correlates of suicide mortality rates in the Flemish region of Belgium, 1996-2005. Suicide & life-threatening behavior.41(4):453-64. | No ethnicity data |
| 304 | Houle JN, Light MT. The harder they fall? Sex and race/ethnic specific suicide rates in the U.S. foreclosure crisis. Social Science and Medicine.180:114-24. | No extractable data |
| 305 | Hourani LL, Davidson L, Clinton-Sherrod M, Patel N, Marshall M, Crosby AE. Suicide prevention and community-level indictors. Evaluation & Program Planning. 2006;29(4):377-85. | No ethnicity data |
| 306 | Howard MO, Hall MT, Edwards JD, Vaughn MG, Perron BE, Winecker RE. Suicide by asphyxiation due to helium inhalation. The American journal of forensic medicine and pathology.32(1):61-70. | No ethnicity data |
| 307 | Howell E, Decker S, Hogan S, Yemane A, Foster J. Declining child mortality and continuing racial disparities in the era of the Medicaid and SCHIP insurance coverage expansions. American journal of public health.100(12):2500-6. | No ethnicity data |
| 308 | Hoyert DL, Freedman MA, Strobino DM, Guyer B. Annual summary of vital statistics: 2000. Pediatrics. 2001;108(6):1241-55. | No ethnicity data |
| 309 | Hoyert DL, Kung HC, Smith BL. Deaths: preliminary data for 2003. National vital statistics reports : from the Centers for Disease Control and Prevention, National Center for Health Statistics, National Vital Statistics System.53(15):1-48. | No ethnicity data |
| 310 | Hu G, Baker S. Reducing black/white disparity: Changes in injury mortality in the 15-24 year age group, United States, 1999-2005. Injury Prevention.14(3):205-8. | No extractable data |
| 311 | Hu G, Wilcox HC, Wissow L, Baker SP. Mid-Life Suicide. An Increasing Problem in U.S. Whites, 1999-2005. American Journal of Preventive Medicine.35(6):589-93. | No extractable data |
| 312 | Hughes BD, Cummins CB, Shan Y, Mehta HB, Radhakrishnan RS, Bowen-Jallow KA. Pediatric firearm injuries: Racial disparities and predictors of healthcare outcomes. Journal of Pediatric Surgery. 2020. | Not general population |
| 313 | Huguet N, Lewis-Laietmark C. Rates of homicide-followed-by-suicide among White, African American, and Hispanic men. Public Health.129(3):280-2. | Not general population |
| 314 | Hull, E, Chowdhury R, Sarnat S, Chang HH, Steenl, et al. Socioeconomic Status and Non-Fatal Adult Injuries in Selected Atlanta (Georgia USA) Hospitals. Prehospital and disaster medicine.32(4):403-13. | No data on suicide |
| 315 | Hummingbird LM. The public health crisis of Native American youth suicide. NASN school nurse (Print).26(2):110-4. | No ethnicity data |
| 316 | Hung P, Busch SH, Shih Y-W, McGregor AJ, Wang S. Changes in community mental health services availability and suicide mortality in the US: A retrospective study. BMC Psychiatry Vol 20 2020, ArtID 188.20. | No ethnicity data |
| 317 | Hunter E. Contextualizing Indigenous suicide. Australian and New Zealand Journal of Psychiatry.45(7):601-2. | No ethnicity data |
| 318 | Hurlbut JL. Suicide: Re-examining factors among Alaskan adolescents. Dissertation Abstracts International Section A: Humanities and Social Sciences.60(11):3877. | Not peer reviewed |
| 319 | Husain N, Martin B, Husain M, Duddu V, Sakinofsky I, Beiser M. Ethnic immigrant suicide in a Canadian psychiatric service: A case series from 1966-1997. European Psychiatry.33:S273. | Not general population |
| 320 | Hutchinson G. Variation of homicidal and suicidal behaviour within Trinidad and Tobago and the associated ecological risk factors. West Indian Medical Journal.54(5):319-24. | No ethnicity data |
| 321 | i A, Prescott MR, Cerda M, Vlahov D, Tardiff KJ, Galea S. Economic conditions and suicide rates in New York city. American Journal of Epidemiology.175(6):527-35. | No extractable data |
| 322 | Ikram UZ, Mackenbach JP, Harding S, Rey G, Bhopal RS, Regidor E, et al. All-cause and cause-specific mortality of different migrant populations in Europe. European Journal of Epidemiology.31(7):655-65. | No ethnicity data |
| 323 | Imrie B. Letter to the editor. Journal of Child and Adolescent Psychiatric Nursing.21(3):125. | Grey literature |
| 324 | Iribarren C, Jacobs DR, Kiefe CI, Lewis CE, Matthews KA, Roseman JM, et al. Causes and demographic, medical, lifestyle and psychosocial predictors of premature mortality: The CARDIA study. Social Science and Medicine.60(3):471-82. | Not general population |
| 325 | Ivey-Stephenson AZ, Crosby AE, Jack SPD, Haileyesus T, Kresnow-Sedacca MJ. Suicide Trends Among and Within Urbanization Levels by Sex, Race/Ethnicity, Age Group, and Mechanism of Death - United States, 2001-2015. Morbidity and mortality weekly report.1-16. | Grey literature |
| 326 | Jack SPD, Petrosky E, Lyons BH, Blair JM, Ertl AM, Sheats KJ, et al. Surveillance for Violent Deaths - National Violent Death Reporting System, , 2015. Morbidity and mortality weekly report.1-32. | Grey literature |
| 327 | Jacobson J, Tollemache J, Campbell KA, Jakubec B, Alonso-Katzowitz JS. 5.58 Investigation of the Role of Outpatient Commitment Laws, Access to Care, and Native American Ethnicity on Suicide Rates in Adolescents by State. Journal of the American Academy of Child and Adolescent Psychiatry.57:S245. | No ethnicity data |
| 328 | Jacobsson L, Stoor JPA, Eriksson A. Suicide among reindeer herding Sami in Sweden, 1961-2017. International journal of circumpolar health.79(1):1754085. | No ethnicity data |
| 329 | Jaiyeola AO, Stabler W. Health disparities among Native American people of the United States. Kosoko-Lasaki, Sade [Ed]; Cook, Cynthia Theresa [Ed]; O'Brien, Richard L [Ed] (2009) Cultural proficiency in addressing health disparities (pp 225-246) xvi, 433 pp Boston, MA, US: Jones and Bartlett Publishers; US. 2009:225-46. | No data on suicide |
| 330 | Jakovljevic M, Sedic B, Martinac M, Marcinko D, Ljubicic D, Vukusic H. Update of suicide trends in croatia 1966-2002. Psychiatria Danubina. 2004;16(4):299-308. | No extractable data |
| 331 | Jalles JT, Andresen MA. The social and economic determinants of suicide in Canadian provinces. Health Economics Review. 2015;5(1). | No ethnicity data |
| 332 | James WA, Frierson RL, Balajepalli B, Lippmann SB. Suicide attempts by burning. The Journal of the Kentucky Medical Association.104(10):459-67. | No data on suicide |
| 333 | Jeon SY, Reither EN, Masters RK. A population-based analysis of increasing rates of suicide mortality in Japan and South Korea, 1985-2010. BMC public health.16:356. | No ethnicity data |
| 334 | Ji J, Kleinman A, Becker AE. Suicide in contemporary China: A review of China's distinctive suicide demographics in their sociocultural context. Harvard Review of Psychiatry. 2001;9(1):1-12. | No ethnicity data |
| 335 | Jiang N. The health of young people in the United States, 1991-2007: Trends in critical national health objectives. Dissertation Abstracts International: Section B: The Sciences and Engineering. 2010;71(2):957. | No ethnicity data |
| 336 | Joe S. Explaining Changes in the Patterns of Black Suicide in the United States From 1981 to 2002: An Age, Cohort, and Period Analysis. Journal of Black Psychology.32(3):262-84. | No ethnicity data |
| 337 | Joe S. Implications of National Suicide Trends for Social Work Practice with Black Youth. Child & Adolescent Social Work Journal. 2006;23(4):458-71. | No data on suicide |
| 338 | Joe S, Kaplan MS. Suicide among African American men. Suicide and Life-Threatening Behavior. 2001;31(1):106-21. | No ethnicity data |
| 339 | Joe S, Kaplan MS. Firearm-related suicide among young African-American males. Psychiatric Services. 2002;53(3):332-4. | Not general population |
| 340 | Joe S, Marcus SC, Kaplan MS. Racial differences in the characteristics of firearm suicide decedents in the United States. American Journal of Orthopsychiatry.77(1):124-30. | No extractable data |
| 341 | Johnson GR, Krug EG, Potter LB. Suicide among adolescents and young adults: a cross-national comparison of 34 countries. Suicide & Life-Threatening Behavior. 2000;30(1):74-82. | No ethnicity data |
| 342 | Jollant F, Macdonald C. Endogamy and suicide: An observation-based hypothesis. Medical Hypotheses.85(5):542-7. | No ethnicity data |
| 343 | Jollant F, Malafosse A, Docto R, Macdonald C. A pocket of very high suicide rates in a non-violent, egalitarian and cooperative population of South-East Asia. Psychological medicine.44(11):2323-9. | No ethnicity data |
| 344 | Jollant F, Malafosse A, Docto R, Macdonald C. Very high rates of suicide in the happy valley: A psychological autopsy study in an isolated indigenous population of South-East Asia. Biological Psychiatry.75(9):212S. | No ethnicity data |
| 345 | Jones H, Rascon M. Suicide in children in El Paso County: A consecutive case series. Modern Pathology.33:12-3. | No extractable data |
| 346 | Jones J, Br, t T, Diaz G, Wang Q, Gary T, et al. Levels of education attainment and income rates to suicide rates in USA: A comprehensive analysis of CDC mortality and US Census data. BMC Proceedings Conference: 16th Annual UT KBRIN Bioinformatics Summit. 2016;11. | No ethnicity data |
| 347 | Jones K, Mansfield CJ. Premature mortality in North Carolina: progress, regress, and disparities by county and race, 2000-2010. North Carolina medical journal. 2014;75(3):159-68. | No extractable data |
| 348 | Jongbloed K, Pearce ME, Pooyak S, Zamar D, Thomas V, Demerais L, et al. The Cedar Project: Mortality among young Indigenous people who use drugs in British Columbia. Cmaj.189(44):E1352-9. | Not general population |
| 349 | Jongbloed K, Zhang H, Thomas V, Pearce M, Christian W, Schechter MT, et al. The cedar project: Predictors of mortality among young aboriginal people who use drugs in British Columbia. Canadian Journal of Infectious Diseases and Medical Microbiology.25:31A. | Not general population |
| 350 | Jordan JT, McNiel DE. Characteristics of persons who die on their first suicide attempt: results from the National Violent Death Reporting System. Psychological medicine.50(8):1390-7. | No extractable data |
| 351 | Jung H, Siklodi Palfi K, Hecser L. Evaluation of suicidal behavior by biostatistical methods. [Romanian]. Romanian Journal of Legal Medicine.9(4):328-35. | No extractable data |
| 352 | Kalesan B, Sampson LA, Zuo Y, Galea S. Sex and age modify the relationship between life circumstances and use of a firearm in suicide deaths across 17 U.S. states. Journal of Affective Disorders.236:105-11. | No extractable data |
| 353 | Kalesan B, Vyliparambil MA, Zuo Y, Siracuse JJ, Fagan JA, Branas CC, et al. Cross-sectional study of loss of life expectancy at different ages related to firearm deaths among black and white Americans. BMJ Evidence-Based Medicine.24(2):55-8. | No extractable data |
| 354 | Kamya HA, White E. Expanding cross-cultural understanding of suicide among immigrants: The case of the Somali. Families in Society.92(4):419-25. | No ethnicity data |
| 355 | Kanamori M, Kondo N. Suicide and Types of Agriculture: A Time-Series Analysis in Japan. Suicide & Life-Threatening Behavior. 2020;50(1):122-37. | No ethnicity data |
| 356 | Kaplan MS, Huguet N, Caetano R, Giesbrecht N, Kerr W, McFarl, et al. The impact of great recession on alcohol-associated suicides: Analysis of the national violent death reporting system. Alcoholism: Clinical and Experimental Research.38:197A. | Grey literature |
| 357 | Kaplan MS, Huguet N, McFarl, BH, Caetano R, Conner KR, et al. Use of alcohol before suicide in the United States. Annals of Epidemiology.24(8):588-92.e2. | No extractable data |
| 358 | Kaplan MS, McFarl, BH, Huguet N, Newsom JT. Sooner versus later: factors associated with temporal sequencing of suicide. Suicide & Life-Threatening Behavior. 2006;36(4):377-85. | No extractable data |
| 359 | Kar N. Factors associated with suicides in Wolverhampton: Relevance of local audits exploring preventability. Medicine, Science & the Law. 2016;56(4):245-51. | No extractable data |
| 360 | Karch DL, Barker L, Strine TW. Race/ethnicity, substance abuse, and mental illness among suicide victims in 13 US states: 2004 Data from the National Violent Death Reporting System. Injury Prevention.12:ii22-ii7. | No extractable data |
| 361 | Karch DL, Logan J, McDaniel DD, Floyd C, Vagi KJ. Precipitating circumstances of suicide among youth aged 10-17 years by sex: Data from the National Violent Death Reporting System, 16 states, 2005-2008. Journal of Adolescent Health.53(1):S51-S3. | No extractable data |
| 362 | Kassem AM, Carter KK, Johnson CJ, Hahn CG. Spatial Clustering of Suicide and Associated Community Characteristics, Idaho, 2010-2014. Preventing chronic disease.16:E37. | No ethnicity data |
| 363 | Kay M. Suicide is leading cause of death in young Indian women, finds international study. BMJ (Clinical research ed). 2013;346. | No ethnicity data |
| 364 | Kelly BD, Davoren M, Mhaolain AN, Breen EG, Casey P. Social capital and suicide in 11 European countries: an ecological analysis. Social Psychiatry & Psychiatric Epidemiology. 2009;44(11):971-7. | No ethnicity data |
| 365 | Kennedy GJ, Tanenbaum S. Suicide and aging: International perspectives. Psychiatric Quarterly.71(4):345-62. | No ethnicity data |
| 366 | Keppel KG, Pearcy JN, Wagener DK. Trends in racial and ethnic-specific rates for the health status indicators: United States, 1990-98. Healthy People 2000 statistical notes / National Center for Health Statistics. (23):1-16. | Grey literature |
| 367 | Keshavan MS, Shenoy S, Li H. Suicide in Asian countries. Asian Journal of Psychiatry.6(5):355. | No ethnicity data |
| 368 | Keyes KM, Liu XC, Cerda M. The role of race/ethnicity in alcohol-attributable injury in the United States. Epidemiologic Reviews.34(1):89-102. | No ethnicity data |
| 369 | Khan MM. Suicide in the Indian subcontinent. Crisis: The Journal of Crisis Intervention and Suicide Prevention. 2000;21(4):193. | No ethnicity data |
| 370 | Khan MM. Suicide on the Indian subcontinent. Crisis. 2002;23(3):104-7. | Duplicate/No ethnicity data |
| 371 | Khan MM, Ahmed A, Khan SR. Female suicide rates in Ghizer, Pakistan. Suicide and Life-Threatening Behavior.39(2):227-30. | No ethnicity data |
| 372 | Khan MM, Hyder AA. Suicides in the developing world: Case study from Pakistan. Suicide and Life-Threatening Behavior.36(1):76-81. | No ethnicity data |
| 373 | Khan MM, Naqvi H, Thaver D, Prince M. Epidemiology of suicide in Pakistan: determining rates in six cities. Archives of Suicide Research. 2008;12(2):155-60. | No ethnicity data |
| 374 | Khan MM, Syed EU. Suicide in Asia: Epidemiology, risk factors, and prevention. O'Connor, Rory C [Ed]; Platt, Stephen [Ed]; Gordon, Jacki [Ed] (2011) International handbook of suicide prevention: Research, policy and practice (pp 487-506) xviii, 677 pp Wiley-Blackwell. 2011:487-506. | No ethnicity data |
| 375 | Kim L, Cr, all M. Asian-Americans and trauma: Unexamined disparities. Journal of Surgical Research.158:291. | No extractable data |
| 376 | Kim Y, Kim H, Gasparrini A, Armstrong B, Honda Y, Chung Y, et al. Suicide and Ambient Temperature: A Multi-Country Multi-City Study. Environmental Health Perspectives. 2019;127(11):117007. | No ethnicity data |
| 377 | Kim Y, Kim H, Honda Y, Guo YL, Chen BY, Woo JM, et al. Suicide and ambient temperature in East Asian Countries: A time-stratified case-crossover analysis. Environmental Health Perspectives.124(1):75-80. | No ethnicity data |
| 378 | Kiny, a E, Wamala D, Musisi S, Hjelmel, H. Suicide in urban Kampala, Uganda: A preliminary exploration. African Health Sciences.11(2):219-27. | No extractable data |
| 379 | Kirkcaldy BD, Furnham A, Siefen RG. Health and educational performance among young migrants. International Journal of Adolescent Medicine and Health.25(1):13-30. | Not general population |
| 380 | Kirmayer LJ. Changing patterns in suicide among young people. Canadian Medical Association Journal.184(9):1015-6. | No ethnicity data |
| 381 | Knipe DW, Padmanathan P, Muthuwatta L, Metcalfe C, Gunnell D. Regional variation in suicide rates in Sri Lanka between 1955 and 2011: a spatial and temporal analysis. BMC public health.17(1):193. | No ethnicity data |
| 382 | Kochanek KD, Arias E, Bastian BA. The Effect of Changes in Selected Age-specific Causes of Death on Non-Hispanic White Life Expectancy Between 2000 and 2014. NCHS data brief. (250):1-8. | No extractable data |
| 383 | Kochanek KD, Smith BL, Anderson RN. Deaths: preliminary data for 1999. National vital statistics reports : from the Centers for Disease Control and Prevention, National Center for Health Statistics, National Vital Statistics System.49(3):1-48. | No ethnicity data |
| 384 | Koenig HG. Association of religious involvement and suicide. JAMA Psychiatry.73(8):775-6. | No ethnicity data |
| 385 | Kohler IV, Preston SH. Ethnic and religious differentials in Bulgarian mortality, 1993-98. Population Studies.65(1):91-113. | No extractable data |
| 386 | Kohn R, Friedmann H. Culture and suicide. Shrivastava, Amresh [Ed]; Kimbrell, Megan [Ed]; Lester, David [Ed] (2012) Suicide from a global perspective: Vulnerable populations and controversies (pp 111-129) x, 180 pp Hauppauge, NY, US: Nova Science Publishers; US. 2012:111-29. | No ethnicity data |
| 387 | Kolves K, de Leo D. Suicide methods in children and adolescents. European Child and Adolescent Psychiatry.26(2):155-64. | No ethnicity data |
| 388 | Kolves K, De Leo D. Suicide rates in children aged 10-14 years worldwide: changes in the past two decades. British Journal of Psychiatry. 2014;205(4):283-5. | No ethnicity data |
| 389 | Kolves K, De Leo D. Adolescent Suicide Rates Between 1990 and 2009: Analysis of Age Group 15-19 Years Worldwide. Journal of Adolescent Health. 2016;58(1):69-77. | No ethnicity data |
| 390 | Kolves K, McDonough M, Crompton D, de Leo D. Choice of a suicide method: Trends and characteristics. Psychiatry Research.260:67-74. | No extractable data |
| 391 | Kolves K, Sisask M, Anion L, Samm A, Varnik A. Factors predicting suicide among Russians in Estonia in comparison with Estonians: Case-control study. Croatian Medical Journal.47(6):869-77. | No extractable data |
| 392 | Kondrichin SV, Lester D. Suicide in the Ukraine. Crisis: The Journal of Crisis Intervention and Suicide Prevention. 2002;23(1):32-3. | No ethnicity data |
| 393 | Kootbodien T, Wilson K, Tlotleng N, Naicker N. Suicide trends by occupation in south africa, 1997 to 2016. Occupational and Environmental Medicine.76:A100. | No ethnicity data |
| 394 | Kornetov N. Depression education in primary care is a basis of suicides prevention. European Psychiatry.30:377. | No ethnicity data |
| 395 | Koronfel AA. Suicide in Dubai, United Arab Emirates. Journal of Clinical Forensic Medicine.9(1):5-11. | No ethnicity data |
| 396 | Kposowa AJ, D'Auria S. Association of temporal factors and suicides in the United States, 2000-2004. Social Psychiatry & Psychiatric Epidemiology. 2010;45(4):433-45. | No extractable data |
| 397 | Kposowa AJ, McElvain JP, Breault KD. Immigration and suicide: The role of marital status, duration of residence, and social integration. Archives of Suicide Research.12(1):82-92. | No extractable data |
| 398 | Kral M. Indigenized suicide prevention among inuit in the Canadian arctic. Journal of the American Academy of Child and Adolescent Psychiatry.55:S2. | No ethnicity data |
| 399 | Kral MJ. Postcolonial Suicide Among Inuit in Arctic Canada. Culture, Medicine and Psychiatry.36(2):306-25. | No extractable data |
| 400 | Kral MJ. Transforming communities: Suicide, relatedness, and reclamation among inuit of Nunavut. Dissertation Abstracts International Section A: Humanities and Social Sciences. 2010;71(3):991. | Grey literature |
| 401 | Kramarow E, Chen L, Hedegaard H, Warner M. Injury deaths among adults aged 65 and over: United States, 2000-2012. Journal of the American Geriatrics Society.63:S39. | No full text |
| 402 | Krieger N, Kim R, Feldman J, Waterman PD. Using the Index of Concentration at the Extremes at multiple geographical levels to monitor health inequities in an era of growing spatial social polarization: Massachusetts, USA (2010-14). International Journal of Epidemiology.47(3):788-819. | No ethnicity data |
| 403 | Kubrin CE, Wadsworth T. Explaining suicide among Blacks and Whites: How socioeconomic factors and gun availability affect race-specific suicide rates. Social Science Quarterly.90(5):1203-27. | No extractable data |
| 404 | Kubrin CE, Wadsworth T, DiPietro S. Deindustrialization, Disadvantage and Suicide among Young Black Males. Social Forces.84(3):1559-79. | Not general population |
| 405 | Kumar PNS, Jayakrishnan, Kumari A. A case-controlled study of suicides in Wayanad district, Kerala. Indian Journal of Psychiatry.52:S2. | No extractable data |
| 406 | Kung A, Hastings KG, Kapphahn KI, Wang EJ, Cullen MR, Ivey SL, et al. Cross-national comparisons of increasing suicidal mortality rates for Koreans in the Republic of Korea and Korean Americans in the USA, 2003-2012. Epidemiology and Psychiatric Sciences.27(1):62-73. | No extractable data |
| 407 | Kung HC, Pearson JL, Liu X. Risk factors for male and female suicide decedents ages 15-64 in the United States. Results from the 1993 National Mortality Followback Survey. Social Psychiatry & Psychiatric Epidemiology. 2003;38(8):419-26. | No ethnicity data |
| 408 | Kung HC, Pearson JL, Wei R. Substance use, firearm availability, depressive symptoms, and mental health service utilization among white and African American suicide decedents aged 15 to 64 years. Annals of Epidemiology.15(8):614-21. | No extractable data |
| 409 | Kwan YK, Ip WC, Kwan P. Gender differences in suicide risk by socio-demographic factors in Hong Kong. Death Studies.29(7):645-63. | No ethnicity data |
| 410 | Kyobutungi C, Ronellenfitsch U, Razum O, Becher H. Mortality from external causes among ethnic German immigrants from former Soviet Union countries, in Germany. European Journal of Public Health.16(4):376-82. | No extractable data |
| 411 | La Luz Baez W. Hispanos in the valley of death: Street-level trauma, cultural-PTSD, overdoses, and suicides in north central New Mexico. Dissertation Abstracts International: Section B: The Sciences and Engineering. 2010;71(2):918. | Grey literature |
| 412 | Lahti A, Harju A, Hakko H, Riala K, Rasanen P. Suicide in children and young adolescents: A 25-year database on suicides from Northern Finland. Journal of Psychiatric Research.58:123-8. | No ethnicity data |
| 413 | Langley J, Broughton J. Injury to Maori. I: Fatalities. The New Zealand medical journal.113(1123):508-10. | No ethnicity data |
| 414 | Lariscy JT, Nau C, Firebaugh G, Hummer RA. Hispanic-White Differences in Lifespan Variability in the United States. Demography.53(1):215-39. | No extractable data |
| 415 | Law CK, Kolves K, De Leo D. Influences of population-level factors on suicides in older adults: A national ecological study from Australia. International Journal of Geriatric Psychiatry.31(4):388-95. | No ethnicity data |
| 416 | Law CK, Kolves K, De Leo D. Suicide mortality in second-generation migrants, Australia, 2001-2008. Social Psychiatry & Psychiatric Epidemiology. 2014;49(4):601-8. | No extractable data |
| 417 | Law S, Liu P. Suicide in China: Unique demographic patterns and relationship to depressive disorder. Current Psychiatry Reports.10(1):80-6. | No ethnicity data |
| 418 | Lawrynowicz AE, Baker TD. Suicide and latitude in Argentina: Durkheim upside-down. American Journal of Psychiatry. 2005;162(5):1022. | No ethnicity data |
| 419 | Lazzarini T, Rohrbaugh RM, Croda J, Goncalves C, Ko A, Benites W, et al. Adolescent suicide among the Guarani-Kaiowa in Dourados, Mato Grosso do Sul, Brazil. Annals of Global Health.81:114. | No ethnicity data |
| 420 | Lazzarini TAM. Clustering of suicide in Brazilian indigenous children and youth: Implications for interventions. Dissertation Abstracts International: Section B: The Sciences and Engineering. 2018;78(11):No Pagination Specified. | Grey literature |
| 421 | Leavitt RA, Ertl A, Sheats K, Petrosky E, Ivey-Stephenson A, Fowler KA. Suicides Among American Indian/Alaska Natives - National Violent Death Reporting System, 18 States, 2003-2014. Mmwr. (8):237-42. | Grey literature |
| 422 | Lee CS, Wong YJ. Racial/Ethnic and Gender Differences in the Antecedents of Youth Suicide. Cultural Diversity and Ethnic Minority Psychology. 2020. | No extractable data |
| 423 | Lee MY-S. Acculturation and suicidal risk among Asian Americans and Latinos in California. Dissertation Abstracts International Section A: Humanities and Social Sciences. 2017;77(10):No Pagination Specified. | No data on suicide |
| 424 | Leineweber M, Arensman E. Culture change and mental health: The epidemiology of suicide in Greenland. Archives of Suicide Research.7(1):41-50. | No ethnicity data |
| 425 | Leineweber M, Bjerregaard P, Baerveldt C, Voestermans P. Suicide in a society in transition. International journal of circumpolar health.60(2):280-7. | No full text |
| 426 | Lemon DD. Killing me softly: A meta-analysis examining risk factors associated with suicide among young African American males. Dissertation Abstracts International: Section B: The Sciences and Engineering. 2009;69(10):6420. | Grey literature |
| 427 | Lester D. Suicide in emigrants from the Indian subcontinent. Transcultural Psychiatry.37(2):243-54. | No extractable data |
| 428 | Lester D. Suicide in Siberian aboriginal groups. Archives of Suicide Research.10(2):221-4. | No ethnicity data |
| 429 | Lester D. Suicide among indigenous peoples: A cross-cultural perspective. Archives of Suicide Research.10(2):117-24. | No ethnicity data |
| 430 | Lester D. Suicide in Ireland: A cross-national view. Irish Journal of Psychological Medicine.27(4):198-204. | No ethnicity data |
| 431 | Lester D. Suicide in Barbados. Columbus, Frank [Ed] (2001) Advances in psychology research, Vol 2 (pp 193-197) vi, 201 pp Hauppauge, NY, US: Nova Science Publishers; US. 2001:193-7. | No ethnicity data |
| 432 | Lester D. Predicting suicide in nations. Archives of Suicide Research. 2005;9(2):219-23. | No ethnicity data |
| 433 | Lester D. Suicide and islam. Archives of suicide research : official journal of the International Academy for Suicide Research. 2006;10(1):77-97. | No ethnicity data |
| 434 | Lester D. Suicide among indigenous peoples: The usefulness of the human relations area files. Crisis. 2008;29(1):49-51. | No ethnicity data |
| 435 | Lester D, Kondrichin S. Genetic risk factors and variation in European suicide rates. The British Journal of Psychiatry.180(3):277-8. | No ethnicity data |
| 436 | Lester D, Krysinska KE. The regional variation of murder and suicide in Poland. Omega: Journal of Death and Dying. 2004;49(4):321-6. | No ethnicity data |
| 437 | Lester D, Saito Y, Park BCB. Suicide among foreign residents of Japan. Psychological Reports.108(1):139-40. | No extractable data |
| 438 | Leung CM, Chung WSD, So EPM. Burning charcoal: An indigenous method of committing suicide in Hong Kong. Journal of Clinical Psychiatry. 2002;63(5):447-50. | No ethnicity data |
| 439 | Levi F, La Vecchia C, Lucchini F, Negri E, Saxena S, Maulik PK, et al. Trends in mortality from suicide, 1965-99. Acta Psychiatrica Scandinavica. 2003;108(5):341-9. | No ethnicity data |
| 440 | Li LW, Xu H, Zhang Z, Liu J. An ecological study of social fragmentation, socioeconomic deprivation, and suicide in rural China: 2008-2010. SSM - Population Health.2:365-72. | No ethnicity data |
| 441 | Li S, Hauser LA, Gao B. Suicide in Travis County, Texas, from 1994 through 1998. Texas medicine.97(5):64-8. | No extractable data |
| 442 | Li X, Xiao Z, Xiao S. Suicide among the elderly in mainland China. Psychogeriatrics. 2009;9(2):62-6. | No ethnicity data |
| 443 | Lin CY, Hsu CY, Gunnell D, Chen YY, Chang SS. Spatial patterning, correlates, and inequality in suicide across 432 neighborhoods in Taipei City, Taiwan. Social Science and Medicine.222:20-34. | No ethnicity data |
| 444 | Lin JJ, Chang SS, Lu TH. The leading methods of suicide in Taiwan, 2002-2008. BMC Public Health. 2010;10:480. | No ethnicity data |
| 445 | Liu C, Li G. Patterns and trends in injury mortality in native american children and adolescents under age 20, 1999-2007. American Journal of Epidemiology.173:S204. | Grey literature |
| 446 | Liu KY. Suicide rates in the world: 1950-2004. Suicide & Life-Threatening Behavior. 2009;39(2):204-13. | No ethnicity data |
| 447 | Liu RT, Kraines MA, Puzia ME, Massing-Schaffer M, Kleiman EM. Sociodemographic predictors of suicide means in a population-based surveillance system: Findings from the National Violent Death Reporting System. Journal of Affective Disorders.151(2):449-54. | No extractable data |
| 448 | Liu S, Page A, Yin P, Astell-Burt T, Feng X, Liu Y, et al. Spatiotemporal variation and social determinants of suicide in China, 2006-2012: findings from a nationally representative mortality surveillance system. Psychological medicine.45(15):3259-68. | No ethnicity data |
| 449 | Loebel JP. Completed suicide in late life. Psychiatric Services. 2005;56(3):260-2. | No ethnicity data |
| 450 | Logan K, Yershova K, zhiyev VA. Suicide trends among young children in the United States from 1993 to 2014. Journal of the American Academy of Child and Adolescent Psychiatry.55:S117. | No extractable data |
| 451 | Loh C, Tai BC, Ng WY, Chia A, Chia BH. Suicide in young singaporeans aged 10-24 years between 2000 to 2004. Archives of Suicide Research.16(2):174-82. | No extractable data |
| 452 | Lorant V, Kunst AE, Huisman M, Bopp M, Mackenbach J. A European comparative study of marital status and socio-economic inequalities in suicide. Social Science & Medicine.60(11):2431-41. | No ethnicity data |
| 453 | Lorant V, Kunst AE, Huisman M, Costa G, Mackenbach J, Health EUWGoS-EIi. Socio-economic inequalities in suicide: a European comparative study. British Journal of Psychiatry. 2005;187:49-54. | No ethnicity data |
| 454 | Lu J, Xiao Y, Xu X, Shi Q, Yang Y. The suicide rates in the Yunnan Province, A multi-ethnic province in Southwestern China. International Journal of Psychiatry in Medicine.45(1):83-96. | No extractable data |
| 455 | Lubin G, Glasser S, Boyko V, Barell V. Epidemiology of suicide in Israel: A nationwide population study. Social Psychiatry and Psychiatric Epidemiology. 2001;36(3):123-7. | No extractable data |
| 456 | Luoma JB, Pearson JL. Suicide and marital status in the United States, 1991-1996: Is widowhood a risk factor? American Journal of Public Health.92(9):1518-22. | No extractable data |
| 457 | Lyons BH, Fowler KA, Jack SP, Betz CJ, Blair JM. Surveillance for Violent Deaths - National Violent Death Reporting System, 17 States, 2013. Morbidity and mortality weekly report.1-42. | No data on suicide |
| 458 | Lyons BH, Walters ML, Jack SPD, Petrosky E, Blair JM, Ivey-Stephenson AZ. Suicides Among Lesbian and Gay Male Individuals: Findings From the National Violent Death Reporting System. American Journal of Preventive Medicine.56(4):512-21. | Not general population |
| 459 | Ma J. The collapse of social life: Marginalization and exit among the Lahu minority in Southwest China. Dissertation Abstracts International Section A: Humanities and Social Sciences. 2012;72(7):2587. | Grey literature |
| 460 | Macaulay A, Orr P, Macdonald S, Elliott L, Brown R, Durcan A, et al. Mortality in the Kivalliq Region of Nunavut, 1987-1996. International journal of circumpolar health. 2004;63:80-5. | No ethnicity data |
| 461 | Macente LB, onade E. Spatial distribution of suicide incidence rates in municipalities in the state of Espirito Santo (Brazil), 2003-2007: Spatial analysis to identify risk areas. Revista Brasileira de Psiquiatria.34(3):261-9. | No ethnicity data |
| 462 | Machado DB, Santos DNd. Suicide in Brazil, from 2000 to 2012. Jornal Brasileiro de Psiquiatria.64(1):45-54. | No extractable data |
| 463 | Mack KA, Jones CM, Ballesteros MF. Illicit Drug Use, Illicit Drug Use Disorders, and Drug Overdose Deaths in Metropolitan and Nonmetropolitan Areas-United States. American Journal of Transplantation.17(12):3241-52. | No ethnicity data |
| 464 | MacPherson DW, Gushulak BD, hu J. Death and international travel - The Canadian experience: 1996 to 2004. Journal of Travel Medicine.14(2):77-84. | No ethnicity data |
| 465 | Maharajah HD. Suicidal behavior in a general hospital: The situation in Trinidad and Tobago. Shrivastava, Amresh [Ed]; Kimbrell, Megan [Ed]; Lester, David [Ed] (2012) Suicide from a global perspective: Risk assessment and management (pp 91-102) xi, 231 pp Hauppauge, NY, US: Nova Science Publishers; US. 2012:91-102. | No extractable data |
| 466 | Maharajh HD, Abdool P, Mohammed-Emamdee R. The theory and practice of Consultation-Liaison (CL) Psychiatry in Trinidad and Tobago with reference to suicidal behavior. TheScientificWorldJournal.8:920-8. | Not general population |
| 467 | Maharajh HD, Abdool PS. Cultural aspects of suicide. TheScientificWorldJournal.5:736-46. | No ethnicity data |
| 468 | Maharajh HD, Abdool PS. Culture and suicide. Merrick, Joav [Ed]; Zalsman, Gil [Ed] (2005) Suicidal behavior in adolescence: An international perspective (pp 19-32) 372 pp London, England: Freund Publishing House; England. 2005:19-32. | Grey literature |
| 469 | Majeed MH, Sherazi SAA, Afzal MY. Decriminalization of suicide in Pakistan-Treatment not punishment. Asian Journal of Psychiatry.35:6-7. | No ethnicity data |
| 470 | Makinen IH. Eastern European transition and suicide mortality. Social Science and Medicine.51(9):1405-20. | No ethnicity data |
| 471 | Makinen IH, Wasserman D. Suicide mortality among immigrant Finnish Swedes. Archives of Suicide Research.7(2):93-106. | No extractable data |
| 472 | Manea MO, Dehelean L, Draghici AM, Enache A, Ciocan V. Risk factors for committed suicide in a Romanian sample. European Psychiatry.30:1805. | No ethnicity data |
| 473 | Maniam T, Chinna K, Lim CH, Kadir AB, Nurashikin I, Salina AA, et al. Suicide prevention program for at-risk groups: Pointers from an epidemiological study. Preventive Medicine. 2013;57:S45-S6. | No data on suicide |
| 474 | Mansfield CJ, Jones K, Imai S. Increased Mortality and Health Risk Behaviors of Midlife White North Carolinians: A Marked Contrast to Nonwhites. North Carolina medical journal.78(6):366-74. | No extractable data |
| 475 | Marcikic M, Ugljarevic M, Dijanic T, Dumencic B, Pozgain I. Epidemiological features of suicides in Osijek County, Croatia, from 1986 to 2000. Collegium Antropologicum. 2003;27:101-10. | No ethnicity data |
| 476 | Marco M, Gracia E, Lopez-Quilez A, Lila M. What calls for service tell us about suicide: A 7-year spatio-temporal analysis of neighborhood correlates of suicide-related calls. Scientific reports.8(1):6746. | No extractable data |
| 477 | Marin-Leon L, De Oliveira HB, Botega NJ. Suicide in Brazil, 2004-2010: The importance of small counties. Revista Panamericana de Salud Publica/Pan American Journal Public Health.32(5):351-9. | No extractable data |
| 478 | Maron BJ, Haas TS, Murphy CJ, Ahluwalia A, Rutten-Ramos S. Incidence and causes of sudden death in U.S. college athletes. Journal of the American College of Cardiology.63(16):1636-43. | Not general population |
| 479 | Maron BJ, Haas TS, Murphy CJ, Ahluwalia A, Rutten-Ramos S. Sudden death in u.s. college athletes: Incidence, causes, and implications for preparticipation screening. Circulation Conference: American Heart Association. 2013;128(22). | Not general population |
| 480 | Marshall CA, Martin WE, Jr., Thomason TC, Johnson MJ. Multiculturalism and rehabilitation counselor training: Recommendations for providing culturally appropriate counseling services to American Indians with disabilities. Marshall, Catherine A [Ed] (2001) Rehabilitation and American Indians with disabilities: A handbook for administrators, practitioners, and researchers (pp 133-160) xx, 236 pp Athens, GA, US: Elliott & Fitzpatrick; US. 2001:133-60. | No extractable data |
| 481 | Martin CA, Unni P, man MP, Feurer ID, McMaster A, Dabrowiak M, et al. Race disparities in firearm injuries and outcomes among Tennessee children. Journal of Pediatric Surgery.47(6):1196-202. | Not general population |
| 482 | Martin JA, Kochanek KD, Strobino DM, Guyer B, MacDorman MF. Annual Summary of Vital Statistics - 2003. Pediatrics.115(3):619-34. | No extractable data |
| 483 | Martin S, Rawala M. Suicide patterns on the London Underground railway system, 2000-2010. BJPsych Bulletin.41(5):275-80. | No extractable data |
| 484 | Martin TG, Sullivan S. Appalling WA state poisoning mortality trends from 1981 to 2005. Clinical Toxicology.47:756. | No data on suicide |
| 485 | Martin TG, Sullivan S. Alarming US poisoning mortality trends from 1981 to 2005. Clinical Toxicology.47:723-4. | No data on suicide |
| 486 | Martin-Gutierrez G, Wall, er JL, Song AV, Elliott MN, Tortolero SR, et al. Health-Related Issues in Latina Youth: Racial/Ethnic, Gender, and Generational Status Differences. Journal of Adolescent Health.61(4):478-85. | Not general population |
| 487 | Martinez Y. Youth mortality by self-asphyxiation U.S.: years 1999-2014. Dissertation Abstracts International: Section B: The Sciences and Engineering. 2020;81(6):No Pagination Specified. | Not general population |
| 488 | Marusic A. Section 1--Suicide behavior in Slovenia: History and regionality of suicide behavior in Slovenia. Crisis: The Journal of Crisis Intervention and Suicide Prevention. 2000;21(4):189-90. | No extractable data |
| 489 | Marusic A. History and geography of suicide: could genetic risk factors account for the variation in suicide rates? American Journal of Medical Genetics Part C, Seminars in Medical Genetics. 2005;133(1):43-7. | No extractable data |
| 490 | Mary Fonseka T, McKinley GP, Kennedy SH. Is tetraethyl lead poison affecting contemporary indigenous suicides in Ontario, Canada? Psychiatry Research.251:253-4. | No extractable data |
| 491 | Masocco M, Pompili M, Vanacore N, Innamorati M, Lester D, Girardi P, et al. Completed suicide and marital status according to the Italian region of origin. Psychiatric Quarterly. 2010;81(1):57-71. | No extractable data |
| 492 | Matsubayashi K, Sakagami T, Wada T, Ishine M, Sakamoto R, Yamanaka G, et al. Mood disorders in community-dwelling older adults in Asia. Journal of the American Geriatrics Society.58(1):213-4. | No extractable data |
| 493 | May PA, Van Winkle NW, Williams MB, McFeeley PJ, Debruyn LM, Serna P. Alcohol and suicide death among American Indians of New Mexico: 1980-1998. Suicide and Life-Threatening Behavior.32(3):240-55. | No extractable data |
| 494 | Mayer P. Female equality and suicide in the Indian states. Psychological reports.92(3):1022-8. | No extractable data |
| 495 | Mayer P, Ziaian T. Suicide, gender, and age variations in India. Are women in indian society protected from suicide? Crisis. 2002;23(3):98-103. | No extractable data |
| 496 | McDermott KM, Brearley MB, Hudson SM, Ward L, Read DJ. Characteristics of trauma mortality in the Northern Territory, Australia. Injury Epidemiology. 2017;4(1). | No extractable data |
| 497 | McDowell R, Fowles J, Phillips D. Deaths from poisoning in New Zealand: 2001-2002. New Zealand Medical Journal. 2005;118(1225):U1725. | No extractable data |
| 498 | McHugh C, Campbell A, Chapman M, Balaratnasingam S. Increasing indigenous self-harm and suicide in the Kimberley: An audit of the 2005-2014 data. Medical Journal of Australia.205(1):33. | No extractable data |
| 499 | McKay K, Milner A, Maple M. Women and suicide: Beyond the gender paradox. International Journal of Culture and Mental Health.7(2):168-78. | No extractable data |
| 500 | McKenzie K. Suicide studies in ethnic minorities: Improving the science to help develop policy. Ethnicity and Health.17(1):7-11. | No extractable data |
| 501 | McKenzie K, Bhui K, Nanchahal K, Blizard B. Suicide rates in people of South Asian origin in England and Wales: 1993-2003. British Journal of Psychiatry.193(5):406-9. | No extractable data |
| 502 | McKenzie K, Bhui K, Nanchahal K, Blizard B. Suicide rates in people of South Asian origin in England and Wales: Correction. The British Journal of Psychiatry.194(5):470. | No extractable data |
| 503 | McKenzie K, Bhul K. Suicide rates in people of South Asian origin in England and Wales: Author's reply. The British Journal of Psychiatry.194(6):567-8. | No extractable data |
| 504 | McKenzie K, Serfaty M, Crawford M. Suicide in ethnic minority groups. British Journal Psychiatry.183:100-1. | No extractable data |
| 505 | McKinnon B, Gariepy G, Sentenac M, Elgar FJ. Adolescent suicidal behaviours in 32 low- and middle-income countries. Bulletin of the World Health Organization.94(5):340-50F. | Not general population |
| 506 | McLoughlin AB, Gould MS, Malone KM. Global trends in teenage suicide: 2003-2014. Qjm.108(10):765-80. | Not general population |
| 507 | McLoughlin DP. Suicide among Canadian First Nations males. Dissertation Abstracts International: Section B: The Sciences and Engineering. 2007;68(1):629. | Grey literature |
| 508 | McNamara PM. Adolescent suicide in Australia: rates, risk and resilience. Clinical child psychology and psychiatry.18(3):351-69. | No extractable data |
| 509 | Meel B. Trends of suicide in the transkei region of South Africa. South African Journal of Psychiatry. 2010;16:109. | No extractable data |
| 510 | Meel B. Trends of suicide in Mthatha region of South Africa over 4-year period from 2009 to 2012. South African Journal of Psychiatry. 2014;20:122. | No extractable data |
| 511 | Meel BL. Determinants of suicide in the Transkei sub-region of South Africa. Journal of Clinical Forensic Medicine.10(2):71-6. | No extractable data |
| 512 | Meel BL. A study of the incidence of suicide by hanging in the sub-region of Transkei, South Africa. Journal of Clinical Forensic Medicine.10(3):153-7. | No extractable data |
| 513 | Meel BL. Suicide among teenagers and young adults in the Transkei. Case reports. Anil Aggrawal's Internet Journal of Forensic Medicine and Toxicology. 2004;5(2). | No extractable data |
| 514 | Merrick J. Trends in adolescent suicide in Israel. International Journal of Adolescent Medicine and Health.12(2):245-8. | Not general population |
| 515 | Mezuk B, Prescott MR, Tardiff K, Vlahov D, Galea S. Suicide in older adults in long-term care: 1990 to 2005. Journal of the American Geriatrics Society.56(11):2107-11. | Not general population |
| 516 | Milic CT. [Age as a suicide risk factor]. Vojnosanitetski Pregled. 2000;57(2):191-5. | No extractable data |
| 517 | Miller M, Azrael D, Barber C. Suicide mortality in the United States: The importance of attending to method in understanding population-level disparities in the burden of suicide. Annual Review of Public Health.33:393-408. | No extractable data |
| 518 | Miller M, Warren M, Hemenway D, Azrael D. Firearms and suicide in US cities. Injury prevention : journal of the International Society for Child and Adolescent Injury Prevention.21:e116-e9. | No extractable data |
| 519 | Milner A, McClure R, De Leo D. Globalization and suicide: an ecological study across five regions of the world. Archives of Suicide Research. 2012;16(3):238-49. | No extractable data |
| 520 | Milner A, McClure R, De Leo D. Socio-economic determinants of suicide: an ecological analysis of 35 countries. Social Psychiatry & Psychiatric Epidemiology. 2012;47(1):19-27. | No extractable data |
| 521 | Minayo MC, Meneghel SN, Cavalcante FG. [Suicide of elderly men in Brazil]. Ciencia & Saude Coletiva. 2012;17(10):2665-74. | No extractable data |
| 522 | Minino A. Mortality among teenagers aged 12-19 years: United States, 1999-2006. NCHS data brief. (37):1-8. | Not general population |
| 523 | Minino AM, Murphy SL, Xu J, Kochanek KD. Deaths: final data for 2008. National vital statistics reports : from the Centers for Disease Control and Prevention, National Center for Health Statistics, National Vital Statistics System.59(10):1-126. | No extractable data |
| 524 | Minino AM, Xu J, Kochanek KD. Deaths: preliminary data for 2008. National Vital Statistics Reports. 2010;59(2):1-52. | No extractable data |
| 525 | Mirsky J, Kohn R, Dolberg P, Levav I. Suicidal behavior among immigrants. Social psychiatry and psychiatric epidemiology.46(11):1133-41. | No extractable data |
| 526 | Mobley PY. High-risk sexual behaviors among asian and pacific islander adolescents in Hawai. Dissertation Abstracts International: Section B: The Sciences and Engineering. 2007;68(2):1340. | Grey Literature |
| 527 | Mohatt JW, Walkup JT. Addressing the crisis of indigenous youth suicide: Opportunities and challenges. Journal of the American Academy of Child and Adolescent Psychiatry.55:S1. | Not general population |
| 528 | Mohler B, Earls F. Trends in adolescent suicide: Misclassification bias? American Journal of Public Health. 2001;91(1):150-3. | Not general population |
| 529 | Mok PL, Leyl, AH, Kapur N, Windfuhr K, Appleby L, et al. Why does Scotland have a higher suicide rate than England? An area-level investigation of health and social factors. Journal of Epidemiology & Community Health. 2013;67(1):63-70. | No extractable data |
| 530 | Mokhtari AM, Sahraian S, Hassanipour S, Baseri A, Mirahmadizadeh A. The epidemiology of suicide in the elderly population in Southern Iran, 2011-2016. Asian Journal of Psychiatry. 2019;44:90-4. | Not general population |
| 531 | Montesinos A, Heredia a, Brom, Z, Aichberger MC, Temur-Erman S, et al. Suicide and suicidal behaviour among female migrants of Turkish descent. Zeitschrift fur Psychiatrie, Psychologie und Psychotherapie.58(3):173-9. | No extractable data |
| 532 | Morar S, Doican I. Ethnical coordinates of accomplished suicide in Sibiu county, 1993-2002. Romanian Journal of Legal Medicine.13(2):155-9. | No extractable data |
| 533 | Moro-Martin A. Spanish changes are scientific suicide. Nature. 2012;482(7385):277. | No extractable data |
| 534 | Moulton D. Suicide rate higher for indigenous people in Labrador. CMAJ : Canadian Medical Association journal = journal de l'Association medicale canadienne.188(12):E275. | No extractable data |
| 535 | Muazzam S, Swahn MH, Alamgir H, Nasrullah M. Differences in poisoning mortality in the United States, 2003-2007: Epidemiology of poisoning deaths classified as unintentional, suicide or homicide. Western Journal of Emergency Medicine.13(3):230-8. | No extractable data |
| 536 | Mukamal KJ, Miller M. BMI and risk factors for suicide: Why is BMI inversely related to suicide? Obesity.17(3):532-8. | No extractable data |
| 537 | Mukamal KJ, Wee CC, Miller M. BMI and rates of suicide in the United States: An ecological analysis. Obesity.17(10):1946-50. | No extractable data |
| 538 | Mullen B, Smyth JM. Immigrant suicide rates as a function of ethnophaulisms: Hate speech predicts death. Psychosomatic Medicine.66(3):343-8. | No extractable data |
| 539 | Munoz M. A case series of child and adolescent suicide in Los Angeles county (2015-2016): Demographic characteristics, communication trends, and risk factors. Dissertation Abstracts International: Section B: The Sciences and Engineering. 2020;81(4):No Pagination Specified. | Not general population |
| 540 | Munson MR. Ethnicity, depression, and suicide. American Journal of Psychiatry. 2002;159(10):1794-5; author reply 5. | No extractable data |
| 541 | Murty OP, Cheh LB, Bakit PA, Hui FJ, Ibrahim ZB, Jusoh NB. Suicide and ethnicity in Malaysia. American Journal of Forensic Medicine and Pathology.29(1):19-22. | No extractable data |
| 542 | Mythri SV, Ebenezer JA. Suicide in India: Distinct Epidemiological Patterns and Implications. Indian Journal of Psychological Medicine. 2016;38(6):493-8. | No extractable data |
| 543 | Nachman R, Yanai O, Goldin L, Swartz M, Barak Y, Hiss J. Suicide in Israel: 1985-1997. Journal of Psychiatry and Neuroscience.27(6):423-8. | No extractable data |
| 544 | Naghavi M. Global, regional, and national burden of suicide mortality 1990 to 2016: Systematic analysis for the Global Burden of Disease Study 2016. BMJ: British Medical Journal Vol 364 2019, ArtID l94.364. | No extractable data |
| 545 | Naidoo SS, Schlebusch L. Sociodemographic characteristics of persons committing suicide in Durban, South Africa: 2006-2007. African journal of primary health care & family medicine.6(1):E1-7. | No extractable data |
| 546 | Nasseri K. Mortality in first generation white immigrants in California, 1989-1999. Journal of Immigrant and Minority Health.10(3):197-205. | No extractable data |
| 547 | Nasseri K, Moulton LH. Patterns of death in the first and second generation immigrants from selected Middle Eastern countries in California. Journal of immigrant and minority health / Center for Minority Public Health.13(2):361-70. | No extractable data |
| 548 | Nettelbladt P, Mattisson C, Bogren M, Holmqvist M. Suicide rates in the lundby cohort before and after the introduction of tricyclic antidepressant drugs. Archives of Suicide Research.11(1):57-67. | No extractable data |
| 549 | Neumayer E. Are socioeconomic factors valid determinants of suicide? Controlling for national cultures of suicide with fixed-effects estimation. Cross-Cultural Research: The Journal of Comparative Social Science.37(3):307-29. | No extractable data |
| 550 | Ngwena J. Black and minority ethnic groups (BME) suicide, admission with suicide or self-harm: An inner city study. Journal of Public Health (Germany).22(2):155-63. | No extractable data |
| 551 | Ni H. Suicides among older adults aged 65 and over in the United States, 2007-2017. Journal of the American Geriatrics Society.67:S6. | No extractable data |
| 552 | Niederkrotenthaler T, Mittendorfer-Rutz E, Mehlum L, Qin P, Bjorkenstam E. Previous suicide attempt and subsequent risk of re-attempt and suicide: Are there differences in immigrant subgroups compared to Swedish-born individuals? Journal of Affective Disorders.265:263-71. | No extractable data |
| 553 | Nielsen AL, Martinez R, Jr. The role of immigration for violent deaths. Homicide Studies: An Interdisciplinary & International Journal.13(3):274-87. | No extractable data |
| 554 | Nunez-Gonzalez S, Lara-Vinueza AG, Gault C, Delgado-Ron JA. Trends and spatial patterns of suicide among adolescent in Ecuador, 1997-2016. Clinical Practice and Epidemiology in Mental Health. 2018;14(1):283-92. | Not general population |
| 555 | o DH, Moreira RS, Pereira JCR, Barrozo LV. Spatial clusters of suicide in the municipality of Sao Paulo 1996-2005: an ecological study. BMC Psychiatry.12. | No extractable data |
| 556 | O'Dwyer ST, De Leo D. Older adults and suicide. Wasserman, Danuta [Ed] (2016) Suicide: An unnecessary death , 2nd ed (pp 215-227) xxxv, 411 pp New York, NY, US: Oxford University Press; US. 2016:215-27. | No extractable data |
| 557 | Ohayon MM. About suicide and its prevention. Journal of Psychiatric Research. 2009;43(10):885-6. | No extractable data |
| 558 | Ojima T, Nakamura Y, Detels R. Comparative study about methods of suicide between Japan and the United States. Journal of epidemiology / Japan Epidemiological Association.14(6):187-92. | No extractable data |
| 559 | Okoye CN, Okoye MI. Forensic epidemiology of childhood deaths in Nebraska, USA. Journal of Forensic and Legal Medicine.18(8):366-74. | Not general population |
| 560 | Olds KL, Tse R, Stables S, Baker AM, Langlois NEI, Byard RW. An analysis of child and adolescent suicide in three centres from 2008-2017. Pathology.52:S104. | Not general population |
| 561 | Oleske DA, Oleske DM, Derrick SM, Hines MO. Suicide trends and risk factors in youth aged 10-19 years, Texas, USA. Pharmacoepidemiology and Drug Safety.25:33. | Not general population |
| 562 | Olfson M, Wall M, Wang S, Crystal S, Bridge JA, Liu SM, et al. Suicide after deliberate self-harm in adolescents and young adults. Pediatrics. 2018;141(4). | No extractable data |
| 563 | Omalu BI, Macurdy KM, Koehler SA, Nnebe-Agumadu UH, Shakir AM, Rozin L, et al. Forensic pathology and forensic epidemiology of suicides in allegheny county, pennsylvania : A 10-year retrospective review (1990-1999). Forensic Science, Medicine & Pathology. 2005;1(2):125-37. | No extractable data |
| 564 | Oquendo MA, Ellis SP, Greenwald S, Malone KM, Weissman MM, Mann JJ. Ethnic and sex differences in suicide rates relative to major depression in the United States. American Journal of Psychiatry. 2001;158(10):1652-8. | No extractable data |
| 565 | Orellana JD, Basta PC, de Souza ML. Mortality by suicide: a focus on municipalities with a high proportion of self-reported indigenous people in the state of Amazonas, Brazil. Revista brasileira de epidemiologia = Brazilian journal of epidemiology.16(3):658-69. | No extractable data |
| 566 | Ortiz-Prado E, Simbana K, Gomez L, Henriquez-Trujillo AR, Cornejo-Leon F, Vasconez E, et al. The disease burden of suicide in Ecuador, a 15years' geodemographic cross-sectional study (2001-2015). BMC Psychiatry. 2017;17(1). | No extractable data |
| 567 | Osterman MJK, Kochanek KD, MacDorman MF, Strobino DM, Guyer B. Annual summary of vital statistics: 2012-2013. Pediatrics.135(6):1115-25. | No extractable data |
| 568 | Ostry A, Maggi S, Tansey J, Dunn J, Hershler R, Chen L, et al. The impact of psychosocial work conditions on attempted and completed suicide among western Canadian sawmill workers. Scandinavian Journal of Public Health. 2007;35(3):265-71. | No extractable data |
| 569 | Osvath P, Balint L, Bozsonyi K. [Lottery and suicide - the effect of number of lottery tickets on Hungarian suicide rates]. Psychiatria Hungarica. 2017;32(3):288-95. | No extractable data |
| 570 | Otsu A, Araki S, Sakai R, Yokoyama K, Scott Voorhees A. Effects of urbanization, economic development, and migration of workers on suicide mortality in Japan. Social Science and Medicine.58(6):1137-46. | No extractable data |
| 571 | Ott JJ, Winkler V, Kyobutungi C, Laki J, Becher H. Effects of residential changes and time patterns on external-cause mortality in migrants: Results of a German cohort study. Scandinavian Journal of Public Health.36(5):524-31. | No extractable data |
| 572 | Page A, Liu S, Gunnell D, Astell-Burt T, Feng X, Wang L, et al. Suicide by pesticide poisoning remains a priority for suicide prevention in China: Analysis of national mortality trends 2006-2013. Journal of Affective Disorders.208:418-23. | No extractable data |
| 573 | Page A, Morrell S, Taylor R, Carter G, Dudley M. Divergent trends in suicide by socio-economic status in Australia. Social Psychiatry and Psychiatric Epidemiology.41(11):911-7. | No extractable data |
| 574 | Palma DCA, Santos ESD, Ignotti E. [Analysis of spatial patterns and characterization of suicides in Brazil from 1990 to 2015]. Cadernos de Saude Publica. 2020;36(4):e00092819. | No extractable data |
| 575 | Pan YJ, Liao SC, Lee MB. Suicide by charcoal burning in Taiwan, 1995-2006. Journal of Affective Disorders. 2010;120(1):254-7. | No extractable data |
| 576 | Papadopoulos FC, Skalkidou A, Sergentanis TN, Kyllekidis S, Ekselius L, Petridou ET. Preventing suicide and homicide in the United States: The potential benefit in human lives. Psychiatry Research.169(2):154-8. | No extractable data |
| 577 | Paraschakis A, Michopoulos I, Christodoulou C, Koutsaftis F, Lykouras L, Douzenis A. Characteristics of immigrant suicide completers in a sample of suicide victims from Greece. The International journal of social psychiatry.60(5):462-7. | No extractable data |
| 578 | Park S. Brief report: Sex differences in suicide rates and suicide methods among adolescents in South Korea, Japan, Finland, and the US. Journal of Adolescence. 2015;40:74-7. | Not general population |
| 579 | Park S, Cho SC, Kim BN, Kim JW, Yoo HJ, Hong JP. Increased use of lethal methods and annual increase of suicide rates in Korean adolescents: comparison with adolescents in the United States. Journal of Child Psychology & Psychiatry & Allied Disciplines. 2014;55(3):258-63. | Not general population |
| 580 | Parker G, Yap HL. Suicide in Singapore: a changing sex ratio over the last decade. Singapore medical journal.42(1):11-4. | No extractable data |
| 581 | Parker R. Mental health issues in aboriginal and torres strait Islanders: An overview and understanding of determining factors. Australian and New Zealand Journal of Psychiatry.53:65-6. | No extractable data |
| 582 | Parks SE, Johnson LL, McDaniel DD, Gladden M, Centers for Disease C, Prevention. Surveillance for violent deaths - National Violent Death Reporting System, 16 states, 2010. Morbidity and mortality weekly report.1-33. | Grey literature |
| 583 | Parsons MA, Barger SD. The US Mortality Crisis: An Examination of Non-Hispanic White Mortality and Morbidity in Yavapai County, Arizona. Journal of Community Health. 2019;44(4):661-7. | No extractable data |
| 584 | Pellegrini LC, Rodriguez-Monguio R. Unemployment, Medicaid provisions, the mental health industry, and suicide. The Social Science Journal.50(4):482-90. | No extractable data |
| 585 | Pepper CM. Suicide in the Mountain West Region of the United States. Crisis.38(5):344-50. | Not general population |
| 586 | Perez-Suarez E, Jimenez-Garcia R, Iglesias-Bouzas M, Serrano A, Porto-Abad R, Casado-Flores J. [Falls from heights in Pediatrics. Epidemiology and evolution of 54 patients]. Medicina Intensiva. 2012;36(2):89-94. | No extractable data |
| 587 | Perkins R, dal TL, Howell M, dal ND, Berman A. Epidemiological and follow-back study of suicides in Alaska. International Journal of Circumpolar Health. 2009;68(3):212-23. | No extractable data |
| 588 | Petchkovsky L, Cord-Udy N, Grant L. A post-Jungian perspective on 55 indigenous suicides in Central Australia; deadly cycles of diminished resilience, impaired nurturance, compromised interiority; and possibilities for repair. AeJAMH (Australian e-Journal for the Advancement of Mental Health).6(3):1-14. | No extractable data |
| 589 | Peterson C, Schumacher PK, Steege AL. Demographic considerations in analyzing decedents by usual occupation. American journal of industrial medicine. 2020;23. | No extractable data |
| 590 | Philcox W, Garl, J, Zaidi F, Morrow P, Kesha K, et al. Higher Heart Weight in New Zealand Maori and Pacific Islanders. The American journal of forensic medicine and pathology.39(3):208-12. | No extractable data |
| 591 | Phillips A. Health status differentials across rural and remote Australia. Australian Journal of Rural Health. 2009;17(1):2-9. | No extractable data |
| 592 | Phillips JA. Factors Associated With Temporal and Spatial Patterns in Suicide Rates Across U.S. States, 1976-2000. Demography. 2013;50(2):591-614. | No extractable data |
| 593 | Pinto LW, da Silva CMFP, Pires TO, de Assis SG. Factors associated with suicide mortality among the elderly in Brazilian municipalities between 2005 and 2007. Ciencia e Saude Coletiva.17(8):2003-9. | No extractable data |
| 594 | Piper TM, Tracy M, Bucciarelli A, Tardiff K, Galea S. Firearm suicide in New York City in the 1990s. Injury Prevention.12(1):41-5. | No extractable data |
| 595 | Pobutsky A, Brown M, Nakao L, Reyes-Salvail F. Results from the Hawaii domestic violence fatality review, 2000-2009. Journal of injury & violence research.6(2):79-90. | No extractable data |
| 596 | Polozhy B. Social and ethno-cultural aspects of the dynamics of the rate of suicides in the CIS countries (1990-present time). European Psychiatry.48:S65. | No extractable data |
| 597 | Polozhy B. The ethnocultural peculiarities of suicides in russia. European Psychiatry Conference: 21st European Congress of Psychiatry, EPA. 2013;28. | No extractable data |
| 598 | Prabhakar H, Manoharan R. The Tribal Health Initiative model for healthcare delivery: a clinical and epidemiological approach. National Medical Journal of India. 2005;18(4):197-204. | No extractable data |
| 599 | Pratt D, Appleby L, Piper M, Webb R, Shaw J. Suicide in recently released prisoners: a case-control study. Psychological medicine.40(5):827-35. | No extractable data |
| 600 | Pridmore S, Kuipers P, Appleton J. The 'Operationalized Predicaments of Suicide' (OPS) applied to Northern Territory coroners' reports. Asian Journal of Psychiatry.6(3):214-7. | No extractable data |
| 601 | Probst J, Zahnd W, Breneman C. Declines In Pediatric Mortality Fall Short For Rural US Children. Health affairs (Project Hope).38(12):2069-76. | No extractable data |
| 602 | Procter NG, Kenny MA, Eaton H, Grech C. Lethal hopelessness: Understanding and responding to asylum seeker distress and mental deterioration. International journal of mental health nursing.27(1):448-54. | No extractable data |
| 603 | Przepyszny LM, Jenkins AJ. The prevalence of drugs in carbon monoxide-related deaths: A retrospective study, 2000-2003. American Journal of Forensic Medicine and Pathology.28(3):242-8. | No extractable data |
| 604 | Pugh MJV, Copel, LA, Zeber JE, Wang CP, Amuan ME, et al. Antiepileptic drug monotherapy exposure and suicide-related behavior in older veterans. Journal of the American Geriatrics Society.60(11):2042-7. | Not general population |
| 605 | Purselle DC, Heninger M, Hanzlick R, Garlow SJ. Differential association of socioeconomic status in ethnic and age-defined suicides. Psychiatry Research.167(3):258-65. | No extractable data |
| 606 | Puzo Q, Mehlum L, Qin P. Suicide among immigrant population in Norway: a national register-based study. Acta Psychiatrica Scandinavica.135(6):584-92. | No extractable data |
| 607 | Puzo Q, Mehlum L, Qin P. Socio-economic status and risk for suicide by immigration background in Norway: A register-based national study. Journal of Psychiatric Research.100:99-106. | No extractable data |
| 608 | Pycha R, Pompili M, Giupponi G. Sex, ethnic and alcohol abuse differences in South Tyrolean suicides: Two psychological autopsy studies. European Psychiatry Conference: 18th European Congress of Psychiatry Munich Germany Conference Publication:. 2010;25. | No extractable data |
| 609 | Qin P. Suicide risk in relation to level of urbanicity - A population-based linkage study. International Journal of Epidemiology.34(4):846-52. | No extractable data |
| 610 | Qin P, Mortensen PB, Pedersen CB. Frequent change of residence and risk of attempted and completed suicide among children and adolescents. Archives of General Psychiatry.66(6):628-32. | No extractable data |
| 611 | Quimby EG, McLone SG, Mason M, Sheehan K. Cross sectional analysis of Chicago vs suburban Cook County suicide deaths among 10-24-year-olds in the Illinois violent death reporting system. Injury Epidemiology.5(1):41-6. | No extractable data |
| 612 | Rahimi R, Ali N, Md Noor S, Mahmood MS, Zainun KA. Suicide in the elderly in Malaysia. Malaysian Journal of Pathology.37(3):259-63. | No extractable data |
| 613 | Raifman J, Sampson L, Galea S. Suicide fatalities in the US compared to Canada: Potential suicides averted with lower firearm ownership in the US. PLoS ONE. 2020;15(4). | No extractable data |
| 614 | Ramesh A, Kumar A, Aram, la MP, Ny, a AM. Polycyclic aromatic hydrocarbon residues in serum samples of autopsied individuals from Tennessee. International Journal of Environmental Research and Public Health.12(1):322-34. | No extractable data |
| 615 | Rao AL, Asif IM, Drezner JA, Toresdahl BG, Harmon KG. Suicide in NCAA athletes: A nine-year analysis of the NCAA resolutions database. Clinical Journal of Sport Medicine.24:168. | Not general population |
| 616 | Ray WA, Hall K, Meador KG. Racial differences in antidepressant treatment preceding suicide in a medicaid population. Psychiatric Services.58(10):1317-23. | No extractable data |
| 617 | Ream GL. An Investigation of the LGBTQ+ Youth Suicide Disparity Using National Violent Death Reporting System Narrative Data. Journal of Adolescent Health.66(4):470-7. | Not general population |
| 618 | Reggianini C, Martire L, Ferrari S, Pizzirani M, Santunione AL, Rigatelli M. Suicidal migrants in Modena. European Psychiatry Conference: 20th European Congress of Psychiatry, EPA. 2012;27. | No extractable data |
| 619 | Regoeczi WC, Gilson T. Homicide-Suicide in Cuyahoga County, Ohio, 1991-2016. Journal of forensic sciences.63(5):1539-44. | No extractable data |
| 620 | Resnick S, Smith RN, Beard JH, Holena D, Reilly PM, Schwab CW, et al. Firearm Deaths in America: Can We Learn from 462,000 Lives Lost? Annals of Surgery.266(3):432-40. | No extractable data |
| 621 | Ribeiro JD, Gutierrez PM, Joiner TE, Kessler RC, Petukhova MV, Sampson NA, et al. Health care contact and suicide risk documentation prior to suicide death: Results from the Army Study to Assess Risk and Resilience in Servicemembers (Army STARRS). Journal of Consulting and Clinical Psychology.85(4):403-8. | Not general population |
| 622 | Riddell CA, Harper S, Cerda M, Kaufman JS. Comparison of rates of firearm and nonfirearm homicide and suicide in black and white non-Hispanic men, by u.S. State. Annals of Internal Medicine.168(10):712-20. | No extractable data |
| 623 | Ring IT, O'Brien JF. Our hearts and minds - What would it take for Australia to become the healthiest country in the world? Medical Journal of Australia.187(8):447-51. | No extractable data |
| 624 | Ringgenberg W, Peek-Asa C, Donham K, Ramirez M. Trends and Characteristics of Occupational Suicide and Homicide in Farmers and Agriculture Workers, 1992-2010. The Journal of rural health : official journal of the American Rural Health Association and the National Rural Health Care Association.34(3):246-53. | No extractable data |
| 625 | Rios MA, dos Anjos KF, Meira SS, Nery AA, Casotti CA. Completeness of the information system for elderly suicide in the state of Bahia. Jornal Brasileiro de Psiquiatria. 2013;62(2):131-8. | No extractable data |
| 626 | Roberts K, Wassenaar D, Canetto SS, Pillay A. Homicide-suicide in Durban, South Africa. Journal of Interpersonal Violence.25(5):877-99. | No extractable data |
| 627 | Roberts SE, Jaremin B, Chalasani P, Rodgers ESE. Suicides among seafarers in UK merchant shipping, 1919-2005. Occupational Medicine.60(1):54-61. | No extractable data |
| 628 | Robinson J, Too LS, Pirkis J, Spittal MJ. Spatial suicide clusters in Australia between 2010 and 2012: A comparison of cluster and non-cluster among young people and adults. BMC Psychiatry. 2016;16(1). | No extractable data |
| 629 | Rock D, Greenberg DM, Hallmayer JF. Increasing seasonality of suicide in Australia 1970-1999. Psychiatry Research.120(1):43-51. | No ethnicity data |
| 630 | Rockett IRH, Caine ED, Connery HS, Nolte KB, Nestadt PS, Nelson LS, et al. Unrecognised self-injury mortality (SIM) trends among racial/ethnic minorities and women in the USA. Injury prevention : journal of the International Society for Child and Adolescent Injury Prevention. 2019;24. | No extractable data |
| 631 | Rockett IRH, Lian Y, Stack S, Ducatman AM, Wang S. Discrepant comorbidity between minority and white suicides: A national multiple cause-of-death analysis. BMC Psychiatry.9. | No extractable data |
| 632 | Rockett IRH, Samora JB, Coben JH. The black-white suicide paradox: Possible effects of misclassification. Social Science and Medicine.63(8):2165-75. | No extractable data |
| 633 | Romero MP, Wintemute GJ. The epidemiology of firearm suicide in the United States. Journal of Urban Health. 2002;79(1):39-48. | No extractable data |
| 634 | Rosales M, Smith SA, Stallones L. Newspaper coverage of injuries affecting the Spanish surname population in two counties in Colorado. Psychological Reports.99(2):651-8. | No data on suicide |
| 635 | Rubenstein A, Wood SK, Levine RS, Hennekens CH. Alarming Trends in Mortality from Firearms Among United States Schoolchildren. American Journal of Medicine.132(8):992-4. | No ethnicity data |
| 636 | Runeson B, Tidemalm D, Dahlin M, Lichtenstein P, Langstrom N. Method of attempted suicide as predictor of subsequent successful suicide: National long term cohort study. BMJ (Online).341(7765):186. | No ethnicity data |
| 637 | Runyan CW, Moracco KE, Dulli L, Butts J. Suicide among North Carolina women, 1989-93: information from two data sources. Injury prevention : journal of the International Society for Child and Adolescent Injury Prevention.9(1):67-72. | No extractable data |
| 638 | rychyn S. Geographic variation in suicide rates: relationships to social factors, migration, and ethnic history. Archives of Suicide Research. 2004;8(4):303-14. | No ethnicity data |
| 639 | Saarela J, Cederstrom A, Rostila M. Birth order and mortality in two ethno-linguistic groups: Register-based evidence from Finland. Social Science and Medicine.158:8-13. | No extractable data |
| 640 | Salib E, Green L. Gender in elderly suicide: Analysis of coroners inquests of 200 cases of elderly suicide in Cheshire 1989-2001. International Journal of Geriatric Psychiatry.18(12):1082-7. | No extractable data |
| 641 | Samuel D, Sher L. Suicidal behavior in Indian adolescents. International Journal of Adolescent Medicine and Health.25(3):207-12. | No ethnicity data |
| 642 | Santos EGO, Vedana KGG, Barbosa IR. Mortality and years of life lost to suicide in Rio Grande do Norte, Brazil, 2000-2015. Acta Scientiarum - Health Sciences. 2019;41(1). | No extractable data |
| 643 | Satar K, Koroush S, Khairollah A. Study of process and causes related to suicide in ham province during 1992-2005. Research Journal of Medical Sciences. 2010;4(3):217-21. | No data on suicide |
| 644 | Sausen J, Bray K, Richmond BS, Reither E. Suicide trends in Wisconsin 1984-1998: Good news for young and old. Wisconsin Medical Journal. 2001;100(2):35-8. | No access to full text |
| 645 | Scheyett A, Bayakly R, Whitaker M. Characteristics and contextual stressors in farmer and agricultural worker suicides in Georgia from 2008-2015. Journal of Rural Mental Health.43(2):61-72. | Not general population |
| 646 | Schild AHE, Nader IW, Pietschnig J, Voracek M. Ethnicity Moderates the Association Between 5-HTTLPR and National Suicide Rates. Archives of Suicide Research.18(1):1-13. | No ethnicity data |
| 647 | Schmitt MW, Williams TL, Woodard KR, Harruff RC. Trends in suicide by carbon monoxide inhalation in king county, Washington: 1996-2009. Journal of Forensic Sciences.56(3):652-5. | No extractable data |
| 648 | Schnittker J, Do D. Pharmaceutical Side Effects and Mental Health Paradoxes among Racial-Ethnic Minorities. Journal of health and social behavior.61(1):4-23. | No data on suicide |
| 649 | Schoenbaum M, Kessler RC, Gilman SE, Colpe LJ, Heeringa SG, Stein MB, et al. Predictors of suicide and accident death in the army study to assess risk and resilience in servicemembers (army starrs) results from the army study to assess risk and resilience in servicemembers (army starrs). JAMA Psychiatry.71(5):493-503. | No extractable data |
| 650 | Schumock GT, Gibbons RD, Lee TA, Joo MJ, Valuck RJ, Stayner LT. Relationship between leukotriene-modifying agent prescriptions dispensed and rate of suicide deaths by county in the US. Drug, Healthcare and Patient Safety. 2011;3(1):47-52. | No extractable data |
| 651 | Schwab-Reese LM, Peek-Asa C. Factors contributing to homicide-suicide: differences between firearm and non-firearm deaths. Journal of Behavioral Medicine. 2019;42(4):681-90. | No extractable data |
| 652 | Searles VB, Valley MA, Hedegaard H, Betz ME. Suicides in urban and rural counties in the United States, 2006-2008. Crisis. 2014;35(1):18-26. | No extractable data |
| 653 | Sela-Shayovitz R. The role of ethnicity and context: Intimate femicide rates among social groups in Israeli society. Violence Against Women.16(12):1424-36. | Not general population |
| 654 | Selek S. Altitude, immigration and suicide rates: a study from Turkey. Psychiatry Investigation. 2013;10(1):89-91. | No ethnicity data |
| 655 | Sena-Ferreira N, Pessoa VF, Boechat-Barros R, Figueiredo AE, Minayo MC. [Risk factors associated with suicides in Palmas in the state of Tocantins, Brazil, between 2006 and 2009 investigated by psycho-social autopsy]. Ciencia & Saude Coletiva. 2014;19(1):115-26. | No extractable data |
| 656 | Shah A, Oommen G, Koshy A. Ethnic elders and their needs. Psychiatry.8(9):358-62. | No ethnicity data |
| 657 | Shah N, Saadat S, Saucedo C, Bruckner T, Singh P, Yoon J, et al. Suicide attempt in California emergency departments in 2011 and its risk factors. Academic Emergency Medicine.26:S223. | No ethnicity data |
| 658 | Shain BN. Increases in rates of suicide and suicide attempts among black adolescents. Pediatrics. 2019;144(5). | No ethnicity data |
| 659 | Shako K. Sociodemographic factors, culture, and suicide in Guyana. Dissertation Abstracts International Section A: Humanities and Social Sciences. 2020;81(10):No Pagination Specified. | No ethnicity data |
| 660 | Sheftall AH, Asti L, Horowitz LM, Felts A, Fontanella CA, Campo JV, et al. Suicide in elementary school-aged children and early adolescents. Pediatrics. 2016;138(4). | No extractable data |
| 661 | Shelef A, Hiss J, Cherkashin G, Berger U, Aizenberg D, Baruch Y, et al. Psychosocial and medical aspects of older suicide completers in Israel: A 10-year survey. International Journal of Geriatric Psychiatry.29(8):846-51. | No extractable data |
| 662 | Shelef L, Nir I, Tatsa-Laur L, Kedem R, Gold N, Bader T, et al. The effect of the Suicide Prevention Program (SPP) on the characteristics of Israeli soldiers who died by suicide after its implementation. European Psychiatry.62:74-81. | No extractable data |
| 663 | Shepherd A, McKenney M, Elkbuli A. Healing our healers: The silent tragedy of physician suicide. American Surgeon.86(4):E213-E5. | No extractable data |
| 664 | Sheyn A, Johnson RF, Mitchell RB. Head and neck firearm injuries in the United States. Otolaryngology - Head and Neck Surgery (United States).153(1):50. | Grey literature |
| 665 | Shiang J. Considering cultural beliefs and behaviors in the study of suicide. Maris, Ronald W [Ed]; Canetto, Silvia Sara [Ed]; McIntosh, John L [Ed]; Silverman, Morton M [Ed] (2000) Review of suicidology, 2000 (pp 226-241) xxii, 282 pp New York, NY, US: Guilford Press; US. 2000:226-41. | No extractable data |
| 666 | Shields LBE, Hunsaker DM, Hunsaker IJC. Suicide: A ten-year retrospective review of Kentucky Medical Examiner cases. Journal of Forensic Sciences.50(3):613-7. | No access to full text |
| 667 | Shiels MS, Chernyavskiy P, Anderson WF, Best AF, Haozous EA, Hartge P, et al. Trends in premature mortality in the USA by sex, race, and ethnicity from 1999 to 2014: an analysis of death certificate data. The Lancet.389(10073):1043-54. | No extractable data |
| 668 | Shoaf K, Sauter C, Bourque LB, Giangreco C, Weiss B. Suicides in LOS Angeles County in relation to the Northridge earthquake. Prehospital and disaster medicine : the official journal of the National Association of EMS Physicians and the World Association for Emergency and Disaster Medicine in association with the Acute Care Foundation. 2004;19(4):307-10. | No extractable data |
| 669 | Shoval G, Schoen G, Vardi N, Zalsman G. Suicide in Ethiopian immigrants in Israel: A case for study of the genetic-environmental relation in suicide. Archives of Suicide Research.11(3):247-53. | No ethnicity data |
| 670 | Silva C, Van Orden KA. Suicide among Hispanics in the United States. Current opinion in psychology.22:44-9. | No ethnicity data |
| 671 | Silveira ML, Wexler L, Chamberlain J, Money K, Spencer RMC, Reich NG, et al. Seasonality of suicide behavior in Northwest Alaska: 1990-2009. Public Health.137:35-43. | No ethnicity data |
| 672 | Simonetti JA, Piegari R, Maynard C, Brenner LA, Mori A, Post EP, et al. Characteristics and Injury Mechanisms of Veteran Primary Care Suicide Decedents with and without Diagnosed Mental Illness. Journal of General Internal Medicine. 2020. | No extractable data |
| 673 | Singh GK, Azuine RE, Siahpush M, Kogan MD. All-cause and cause-specific mortality among US youth: socioeconomic and rural-urban disparities and international patterns. Journal of urban health : bulletin of the New York Academy of Medicine.90(3):388-405. | No ethnicity data |
| 674 | Singh GK, Kim IE, Girmay M, Perry C, Daus GP, Vedamuthu IP, et al. Opioid Epidemic in the United States: Empirical Trends, and A Literature Review of Social Determinants and Epidemiological, Pain Management, and Treatment Patterns. International Journal of MCH & AIDS. 2019;8(2):89-100. | No ethnicity data |
| 675 | Singh GK, Kogan MD, Slifkin RT. Widening disparities in infant mortality and life expectancy between appalachia and the rest of the United States, 1990-2013. Health Affairs.36(8):1423-32. | No ethnicity data |
| 676 | Singh GK, Siahpush M. Widening rural-urban disparities in life expectancy, U.S., 1969-2009. American Journal of Preventive Medicine.46(2):e19-e29. | No ethnicity data |
| 677 | Singh GK, Siahpush M. Widening rural-urban disparities in all-cause mortality and mortality from major causes of death in the USA, 1969-2009. Journal of urban health : bulletin of the New York Academy of Medicine.91(2):272-92. | No ethnicity data |
| 678 | Singh GK, Siahpush M. Increasing rural-urban gradients in US suicide mortality, 1970-1997. American Journal of Public Health. 2002;92(7):1161-7. | No ethnicity data |
| 679 | Sinyor M, Williams M, Vincent M, Schaffer A, Yip PSF, Gunnell D. Suicide deaths by gas inhalation in Toronto: An observational study of emerging methods of suicide. Journal of Affective Disorders.243:226-31. | No extractable data |
| 680 | Sipila P, Martikainen P. Language-group mortality differentials in Finland in 1988-2004: Assessment of the contribution of cause of death, sex and age. European Journal of Public Health.19(5):492-8. | No extractable data |
| 681 | Sircar K, Clower J, Shin MK, Bailey C, King M, Yip F. Carbon monoxide poisoning deaths in the United States, 1999 to 2012. American Journal of Emergency Medicine.33(9):1140-5. | No ethnicity data |
| 682 | Skopp NA, Trofimovich L, Grimes J, Oetjen-Gerdes L, Gahm GA. Relations between suicide and traumatic brain injury, psychiatric diagnoses, and relationship problems, active component, U.S. Armed Forces, 2001-2009. Msmr.19(2):7-11. | No ethnicity data |
| 683 | Smith NDL, Kawachi I. State-level social capital and suicide mortality in the 50 U.S. states. Social Science and Medicine.120:269-77. | No extractable data |
| 684 | Snowdon J. Should the recently reported increase in Australian suicide rates alarm us? Australian and New Zealand Journal of Psychiatry.51(8):766-9. | No ethnicity data |
| 685 | Snowdon J. Changes in the age pattern of New Zealand suicide rates. New Zealand Medical Journal.130(1448):18-26. | No extractable data |
| 686 | Snowdon J, Draper B, Wyder M. Age variation in the prevalence of DSM-IV disorders in cases of suicide of middle-aged and older persons in Sydney. Suicide & life-threatening behavior.41(4):465-70. | No extractable data |
| 687 | Souza MLP, Onety RJ. Characteristics of suicide mortality among indigenous and non-indigenous people in Roraima, Brazil, 2009-2013. Epidemiologia e Servicos de Saude. 2017;26(4):887-93. | No extractable data |
| 688 | Spaulding AC, Seals RM, McCallum VA, Perez SD, Brzozowski AK, Steenl, et al. Prisoner survival inside and outside of the institution: Implications for health-care planning. American Journal of Epidemiology.173(5):479-87. | No ethnicity data |
| 689 | Speldewinde PC, Cook A, Davies P, Weinstein P. The hidden health burden of environmental degradation: Disease comorbidities and dryland salinity. EcoHealth.8(1):82-92. | No ethnicity data |
| 690 | Spennemann D. Suicides of Punjabi hawkers in 19<sup>th</sup>- and early 20<sup>th</sup>-century Australia. Indian Journal of Psychiatry.61(4):347-51. | No extractable data |
| 691 | Stack S. Suicide among social workers: a research note. Archives of Suicide Research. 2004;8(4):379-88. | No ethnicity data |
| 692 | Stack S. Suicide risk among physicians: a multivariate analysis. Archives of Suicide Research. 2004;8(3):287-92. | No ethnicity data |
| 693 | Stack S, Lester D. Body mass and suicide risk. Crisis. 2007;28(1):46-7. | No ethnicity data |
| 694 | Stack S, Wasserman I. Social and racial correlates of Russian roulette. Suicide & life-threatening behavior.38(4):436-41. | No extractable data |
| 695 | Stack S, Wasserman I. Gender and suicide risk: The role of wound site. Suicide and Life-Threatening Behavior.39(1):13-20. | No extractable data |
| 696 | Stack S, Wasserman I. Race and method of suicide: Culture and opportunity. Archives of Suicide Research. 2005;9(1):57-68. | No extractable data |
| 697 | Staehr MA, Munk-Andersen E. Suicide and suicidal behaviors among asylum seekers in Denmark during the period 2001-2003: A retrospective study. [Danish]. Ugeskrift for Laeger.168(17):1650-2. | No data on suicide |
| 698 | Stallones L. Suicide and potential occupational exposure to pesticides, Colorado 1990-1999. Journal of Agromedicine. 2006;11(3):107-12. | No extractable data |
| 699 | Stallones L, Doenges T, Dik BJ, Valley MA. Occupation and suicide: Colorado, 2004-2006. American Journal of Industrial Medicine.56(11):1290-5. | No ethnicity data |
| 700 | Stanganelli V, Callaghan L, Prince T, Hansen B, Sutton P, Howe A, et al. Like sparkle in sugar cane. Cluster of youth suicide. Neuropsychiatrie de l'Enfance et de l'Adolescence.60(5):S58. | No ethnicity data |
| 701 | Stark K, Joubert G, Struwig M, Pretorius M, van der Merwe N, Botha H, et al. Suicide cases investigated at the state mortuary in Bloemfontein, 2003-2007. South African Family Practice.52(4):332-5. | No extractable data |
| 702 | Steenl, K, Halperin W, Hu S, Walker JT. Deaths due to injuries among employed adults: The effects of socioeconomic class. Epidemiology.14(1):74-9. | No ethnicity data |
| 703 | Stefanidou ME, Maravelias CP, Dona AA, Pistos CM, Spiliopoulou CA, Athanaselis SA. Carbon monoxide-related deaths in Greece: a 23-year survey. The American journal of forensic medicine and pathology.33(2):128-31. | No ethnicity data |
| 704 | Stein EM, Gennuso KP, Ugboaja DC, Remington PL. The Epidemic of Despair Among White Americans: Trends in the Leading Causes of Premature Death, 1999-2015. American journal of public health.107(10):1541-7. | No extractable data |
| 705 | Stephen Bridges F, Kunselman JC. Premature mortality due to suicide, homicide, and motor vehicle accidents in health service delivery areas: Comparison of status Indians in British Columbia, Canada, with all other residents. Psychological Reports.97(3):739-49. | No extractable data |
| 706 | Stewart PA. Analysis of educational attainment and suicide in African American females. Dissertation Abstracts International: Section B: The Sciences and Engineering. 2006;67(1):562. | Grey literature |
| 707 | Stillion JM, Noviello SB. Living and dying in different worlds: Gender differences in violent death and grief. Illness, Crisis, & Loss.9(3):247-59. | No ethnicity data |
| 708 | Straif-Bourgeois S, Ratard R. Firearm-related mortality, Louisiana 1999-2010. The Journal of the Louisiana State Medical Society : official organ of the Louisiana State Medical Society.166(4):168-74. | No extractable data |
| 709 | Straif-Bourgeois S, Ratard R. Suicide mortality rates in Louisiana, 1999-2010. The Journal of the Louisiana State Medical Society : official organ of the Louisiana State Medical Society. 2012;164(5):274-6, 9, 81-82. | No extractable data |
| 710 | Straka L, Novomesky F, Krajcovic J, Macko V. Ritual suicide of Japanese girl in the Slovak Republic. Legal Medicine.11:S506-S7. | No extractable data |
| 711 | Studdert DM, Zhang Y, Swanson SA, Prince L, Rodden JA, Holsinger EE, et al. Handgun ownership and suicide in California. New England Journal of Medicine.382(23):2220-9. | No ethnicity data |
| 712 | Sukhai A, Harris C, Moorad RGR, Dada MA. Suicide by self-immolation in Durban, South Africa: A five-year retrospective review. American Journal of Forensic Medicine and Pathology.23(3):295-8. | No extractable data |
| 713 | Sullivan EM, Annest JL, Simon TR, Luo F, Dahlberg LL. Suicide trends among persons aged 10-24 years--United States, 1994-2012. Mmwr. (8):201-5. | No ethnicity data |
| 714 | Sumarokov Y, Brenn T, Kudryavtsev A, Nilssen O. Socio-demographic characteristics of suicides in the nenets autonomous Okrug, Northwestern Russia. European Psychiatry.30:949. | No ethnicity data |
| 715 | Sumarokov YA, Brenn T, Kudryavtsev AV, Nilssen O. Variations in suicide method and in suicide occurrence by season and day of the week in Russia and the Nenets Autonomous Okrug, Northwestern Russia: A retrospective population-based mortality study. BMC Psychiatry. 2015;15(1). | No extractable data |
| 716 | Sumarokov YA, Brenn T, Kudryavtsev AV, Sidorenkov O, Nilssen O. Alcohol and suicide in the Nenets Autonomous Okrug and Arkhangelsk Oblast, Russia. International journal of circumpolar health. 2016;75:30965. | No ethnicity data |
| 717 | Sun S, Borowsky LH, Tobey M. Major causes of death among tribal communities in South dakota. Journal of General Internal Medicine.34:S104-S5. | No ethnicity data |
| 718 | Sundaram V, Qin P, Zollner L. Suicide risk among persons with foreign background in Denmark. Suicide and Life-Threatening Behavior. 2006;36(4):481-9. | No extractable data |
| 719 | Sundquist K, Hamano T, Li X, Kawakami N, Shiwaku K, Sundquist J. Linking social capital and mortality in the elderly: A Swedish national cohort study. Experimental Gerontology.55:29-36. | No ethnicity data |
| 720 | Sveticic J, Milner A, De Leo D. Contacts with mental health services before suicide: A comparison of Indigenous with non-Indigenous Australians. General Hospital Psychiatry.34(2):185-91. | No extractable data |
| 721 | Sweeney HA, Fontanella CA, Steelesmith DL, Quinn C. Suicide in Older Adults in Ohio: Characteristics, Precipitants, and Mental Health Service Utilization. Community mental health journal. 2020;27. | No extractable data |
| 722 | Tait G, Carpenter B. Firearm suicide in Queensland. Journal of Sociology.46(1):83-98. | No extractable data |
| 723 | Tan JT, Letchuman Ramanathan GR, Choy MP, Raman L, Lim BK. Paraquat poisoning: Experience in Hospital Taiping (year 2008-october 2011). Medical Journal of Malaysia.68(5):384-8. | No ethnicity data |
| 724 | Tapia Granados JA, Diez Roux AV. Life and death during the Great Depression. Proceedings of the National Academy of Sciences of the United States of America.106(41):17290-5. | No ethnicity data |
| 725 | Taylor R. Fiji's Move into the 21st Century. Marsella, Anthony J [Ed]; Austin, Ayda Aukahi [Ed]; Grant, Bruce [Ed] (2005) Social change and psychosocial adaptation in the Pacific Islands: Cultures in transition (pp 107-131) xviii, 307 pp New York, NY, US: Springer Science + Business Media; US. 2005:107-31. | No ethnicity data |
| 726 | Taylor R, Page A, Morrell S, Harrison J, Carter G. Social and psychiatric influences on urban-rural differentials in Australian suicide. Suicide and Life-Threatening Behavior.35(3):277-90. | No ethnicity data |
| 727 | Teasley ML, Washington RO, McCarley LD. A postmodern perspective on black suicides in the United States (revisited). See, Letha A (Lee) [Ed] (2007) Human behavior in the social environment from an African-American perspective , 2nd ed (pp 715-738) xliii, 782 pp New York, NY, US: Haworth Press; US. 2007:715-38. | No extractable data |
| 728 | Thompson R, Kane VR, Sayers SL, Brown GK, Coyne JC, Katz IR. An assessment of suicide in an urban VA Medical Center. Psychiatry.65(4):326-37. | No extractable data |
| 729 | Tiatia-Seath J, Lay-Yee R, R V, ow M. Suicide mortality among pacific peoples in New Zealand, 1996-2013. New Zealand Medical Journal.130(1454):21-9. | No extractable data |
| 730 | Tobias M, Jackson G. Avoidable mortality in New Zealand, 1981-97. Australian and New Zealand Journal of Public Health. 2001;25(1):12-20. | No data on suicide |
| 731 | Tondo L, Albert MJ, Baldessarini RJ. Suicide rates in relation to health care access in the United States: An ecological study. Journal of Clinical Psychiatry.67(4):517-23. | No ethnicity data |
| 732 | Trovato F. Canadian Indian mortality during the 1980s. Social biology. 2000;47(1):135-45. | No extractable data |
| 733 | Turnbull P, Webb R, Kapur N, Clements C, Bergen H, Hawton K, et al. Variation by ethnic group in premature mortality risk following self-harm: A multicentre cohort study in England. BMC Psychiatry. 2015;15(1). | Not general population |
| 734 | Van Hooijdonk C, Droomers M, Deerenberg IM, Mackenbach JP, Kunst AE. Higher mortality in urban neighbourhoods in the Netherlands: Who is at risk? Journal of Epidemiology and Community Health.62(6):499-505. | No ethnicity data |
| 735 | Van Hooijdonk C, Droomers M, Deerenberg IM, Mackenbach JP, Kunst AE. The diversity in associations between community social capital and health per health outcome, population group and location studied. International Journal of Epidemiology. 2008;37(6):1384-92. | No ethnicity data |
| 736 | van Spijker BA, Graafsma T, Dullaart HI, Kerkhof AJ. Impulsive but fatal self-poisoning with pesticides among South Asians in Nickerie, Suriname. Crisis: Journal of Crisis Intervention & Suicide. 2009;30(2):102-5. | No extractable data |
| 737 | Vannoy SD, Andrews BK, Srebnik D. Suicide After Evaluation for Involuntary Psychiatric Commitment-Who Gets Them and What Influences Survival Time? Suicide & life-threatening behavior.46(5):634-46. | Not general population |
| 738 | Vick F, Grubits S. The psychological context of Guarani-Kaiowa who commit suicide in the city of Dourados, Mate Grosso do Sui, Brazil. Guimaraes, Danilo Silva [Ed] (2016) Amerindian paths: Guiding dialogues with psychology (pp 209-223) xxxiii, 331 pp Charlotte, NC, US: IAP Information Age Publishing; US. 2016:209-23. | Grey literature |
| 739 | Vieweg WVR, urangi AK, Anum EA, Lanier JO, Fierro MF, Fern, et al. Toxicology findings in child and adolescent suicides in Virginia: 1987-2003. Primary Care Companion to the Journal of Clinical Psychiatry. 2006;8(3):142-6. | No extractable data |
| 740 | Vigil NH, Grant AR, Perez O, Blust RN, Chikani V, Vadeboncoeur TF, et al. Death by Suicide-The EMS Profession Compared to the General Public. Prehospital Emergency Care. 2019;23(3):340-5. | No extractable data |
| 741 | Vigod SN, Arora S, Urquia ML, Dennis CL, Fung K, Grigoriadis S, et al. Postpartum self-inflicted injury, suicide, assault and homicide in relation to immigrant status in Ontario: a retrospective population-based cohort study. CMAJ open. 2019;7(2):E227-E35. | No data on suicide |
| 742 | Vijayakumar L, Mohanraj R, Kumar S, Jeyaseelan V, Sriram S, Shanmugam M. CASP - An intervention by community volunteers to reduce suicidal behaviour among refugees. The International journal of social psychiatry.63(7):589-97. | No extractable data |
| 743 | Violanti JM. Suicide or undetermined? A national assessment of police suicide death classification. International Journal of Emergency Mental Health.12(2):89-94. | No extractable data |
| 744 | Violanti JM, Robinson CF, Shen R. Law enforcement suicide: a national analysis. International journal of emergency mental health. 2013;15(4):289-97. | No extractable data |
| 745 | Vo LUT, Anderson C, Thomas S, Vernia H, Howe JK, Tyroch AH, et al. Penetrating trauma in children on the United States-Mexico border: Hispanic ethnicity is not a risk factor. Injury.49(7):1358-64. | No data on suicide |
| 746 | von Borczyskowski A, Hjern A, Lindblad F, Vinnerljung B. Suicidal behaviour in national and international adult adoptees: A Swedish cohort study. Social Psychiatry and Psychiatric Epidemiology: The International Journal for Research in Social and Genetic Epidemiology and Mental Health Services.41(2):95-102. | No extractable data |
| 747 | Voracek M. Suicide rate and skin color. Perceptual & Motor Skills. 2006;102(3):836-8. | No ethnicity data |
| 748 | Voracek M, Vintila M, Muranyi D. A further test of the Finno-Ugrian suicide hypothesis: Correspondence of county suicide rates in Romania and population proportion of ethnic Hungarians. Perceptual and Motor Skills.105(3):1209-22. | No ethnicity data |
| 749 | Wadsworth T, Kubrin CE. Hispanic suicide in U.S. metropolitan areas: Examining the effects of immigration, assimilation, affluence, and disadvantage. American Journal of Sociology.112(6):1848-85. | No extractable data |
| 750 | Walker JT. County level suicide rates and social integration: Urbanicity and its role in the relationship. Sociological Spectrum.29(1):101-35. | No ethnicity data |
| 751 | Wallace ME, Hoyert D, Williams C, Mendola P. Pregnancy-associated homicide and suicide in 37 US states with enhanced pregnancy surveillance. American Journal of Obstetrics and Gynecology.215(3):364.e1-.e10. | No extractable data |
| 752 | Walsh M, Grey C. The contribution of avoidable mortality to the life expectancy gap in Maori and Pacific populations in New Zealand-A decomposition analysis. New Zealand Medical Journal.132(1492):46-60. | No extractable data |
| 753 | Wang J, Ho E, Au P, Cheung G. Late-life suicide in Asian people living in New Zealand: a qualitative study of coronial records. Psychogeriatrics.18(4):259-67. | No extractable data |
| 754 | Wang Z, Yu C, Wang J, Bao J, Gao X, Xiang H. Age-period-cohort analysis of suicide mortality by gender among white and black Americans, 1983-2012. International Journal for Equity in Health. 2016;15(1). | No extractable data |
| 755 | Wasserman I, Stack S. Race, urban context, and Russian roulette: findings from the National Violent Death Reporting System, 2003-2006. Suicide & life-threatening behavior.41(1):33-40. | No extractable data |
| 756 | Wasserman IM, Stack S. The relationship between occupation and suicide among African American males: Ohio, 1989-1991. Maris, Ronald W [Ed]; Canetto, Silvia Sara [Ed]; McIntosh, John L [Ed]; Silverman, Morton M [Ed] (2000) Review of suicidology, 2000 (pp 242-251) xxii, 282 pp New York, NY, US: Guilford Press; US. 2000:242-51. | No access to full text |
| 757 | Way BB, Miraglia R, Sawyer DA, Beer R, Eddy J. Factors related to suicide in New York state prisons. International Journal of Law and Psychiatry.28(3):207-21. | Not general population |
| 758 | Weinberger LE, Sreenivasan S, Sathyavagiswaran L, Markowitz E. Child and adolescent suicide in a large, urban area: Psychological, demographic, and situational factors. Journal of Forensic Sciences. 2001;46(4):902-7. | No access to full text |
| 759 | Weiss J. Imported suicide rate. [German]. Deutsche Medizinische Wochenschrift. 2009;134(11). | No full-text available |
| 760 | Welton RS. The management of suicidality: assessment and intervention. Psychiatry. 2007;4(5):24-34. | No ethnicity data |
| 761 | Wexler L, Hill R, Bertone-Johnson E, Fenaughty A. Correlates of Alaska Native fatal and nonfatal suicidal behaviors 1990-2001. Suicide & life-threatening behavior.38(3):311-20. | No ethnicity data |
| 762 | Wexler L, Silveira ML, Bertone-Johnson E. Factors Associated with Alaska Native Fatal and Nonfatal Suicidal Behaviors 2001-2009: Trends and Implications for Prevention. Archives of Suicide Research.16(4):273-86. | No ethnicity data |
| 763 | Wick R, Gilbert JD, Felgate P, Byard RW. Inhalant deaths in South Australia: A 20-year retrospective autopsy study. American Journal of Forensic Medicine and Pathology.28(4):319-22. | No extractable data |
| 764 | Wiebe DJ. Homicide and suicide risks associated with firearms in the home: A national case-control study. Annals of Emergency Medicine.41(6):771-82. | No extractable data |
| 765 | Wild P, Bovio N, Canu IG. Modelling mortality by suicide among women at work in the swiss national cohort. Occupational and Environmental Medicine.76:A62. | No ethnicity data |
| 766 | Willis LA. The silent endemic: A sociological investigation of African-American suicide. Dissertation Abstracts International Section A: Humanities and Social Sciences. 2005;65(12):4729. | No ethnicity data |
| 767 | Willis LA, Coombs DW, Drentea P, Cockerham WC. Uncovering the Mystery: Factors of African American Suicide. Suicide and Life-Threatening Behavior.33(4):412-29. | No extractable data |
| 768 | Wilson AL. The state of South Dakota's child: 2007, part II--death during childhood. South Dakota medicine : the journal of the South Dakota State Medical Association.61(2):47-51. | No extractable data |
| 769 | Wong MD, Chung AK, Boscardin WJ, Li M, Hsieh HJ, Ettner SL, et al. The contribution of specific causes of death to sex differences in mortality. Public Health Reports.121(6):746-54. | No extractable data |
| 770 | Wong O, Harris F, Rosamilia K, Raabe GK. Updated mortality study of workers at a petroleum refinery in Torrance, California, 1959 to 1997. Journal of Occupational and Environmental Medicine. 2001;43(12):1089-102. | No ethnicity data |
| 771 | Wong Y, Deng K, Lee CS, Grimes J, Li P. Asian Pacific Islander Americans' and White Americans' suicide methods. Asian American Journal of Psychology.9(4):318-26. | No extractable data |
| 772 | Wong YJ, Wang L, Li S, Liu H. Circumstances preceding the suicide of Asian Pacific Islander Americans and White Americans. Death studies.41(5):311-7. | No extractable data |
| 773 | Woo JM, Gibbons RD, Rogers CA, Qin P, Kim JB, Roberts DW, et al. Pollen counts and suicide rates. Association not replicated. Acta Psychiatrica Scandinavica. 2012;125(2):168-75. | No extractable data |
| 774 | Woodl, JM. Native American youth and suicide: Mediators and moderators of the relationship between being Native American and suicidality. Dissertation Abstracts International Section A: Humanities and Social Sciences. 2017;77(9):No Pagination Specified. | No ethnicity data |
| 775 | Wright N, Roesler J, Heinen M. The Unequal Burden of Suicide among Minnesotans: Three Strategies for Prevention. Minnesota medicine.98(10):37-9. | No ethnicity data |
| 776 | Wu C, Lathrop S, Reichard RR. A 20 year retrospective review of traumatic spinal cord injuries at a statewide medical examiner's office. Journal of Neuropathology and Experimental Neurology.69:547. | No ethnicity data |
| 777 | Wurdeman TD, Peters AW, Alkire B, Shrime MG. Firearm Prevalence and Pediatric Firearm Homicides: A State-Level Panel Data Analysis. Journal of the American College of Surgeons.229:S153-S4. | No ethnicity data |
| 778 | Xu J, Kochanek KD, Murphy SL, Arias E. Mortality in the United States, 2012. NCHS data brief. (168):1-8. | No ethnicity data |
| 779 | Yamane GK, Butler JL. Suicide burden in the U.S. Air Force: 1990-2004. Military medicine.174(10):1019-23. | No ethnicity data |
| 780 | Yang J, Kang C, Li J, Li P, Zhao X. A three-decade repeated cross-sectional survey on mental health of the Chinese Jino minority. Australian and New Zealand Journal of Psychiatry.51(11):1134-41. | No extractable data |
| 781 | Yang X, Liu T-B, Yang H, Shu M-Y, Hu C-Y, Jin D, et al. The causes of suicide cluster of 12 workers committed and attempted suicide in Foxconn's Shenzhen factory: A retrospective study. Chinese Mental Health Journal.26(2):120-3. | No access to full text |
| 782 | Yildirim Y. Relationship between socioeconomic factors and suicide in North Carolina counties, 1998-2002. Dissertation Abstracts International: Section B: The Sciences and Engineering. 2012;72(11):6660. | No ethnicity data |
| 783 | Yogesh AG, Chang M, Ada A, Narayanan A, Dorothy C. 3.61 Youth Suicide in Saipan and the Introduction of a Prevention Program. Journal of the American Academy of Child and Adolescent Psychiatry.58:S214-S5. | No access to full text |
| 784 | Young TW, Wooden SE, Dew PC, Hoff GL, Cai J. The Richard Cory phenomenon: Suicide and wealth in Kansas City, Missouri. Journal of Forensic Sciences.50(2):443-7. | No extractable data |
| 785 | Yusin T, Hoof M, Hummel LA, Nguyen T, Friedman J, McGrew P, et al. Analysis of pediatric firearm head and neck injuries. Otolaryngology - Head and Neck Surgery.161:P127. | No ethnicity data |
| 786 | Zaki MKL, Ekram T, ae M. Trends of suicide in Jeddah, Kingdom of Saudi Arabia. Rechtsmedizin.21:365. | No ethnicity data |
| 787 | Zammit S, Gunnell D, Lewis G, Leckie G, Dalman C, Allebeck P. Individual- and area-level influence on suicide risk: a multilevel longitudinal study of Swedish schoolchildren. Psychological medicine.44(2):267-77. | No extractable data |
| 788 | Zang E, Zheng H, Yang YC, KC. Recent trends in US mortality in early and middle adulthood: Racial/ethnic disparities in inter-cohort patterns. International Journal of Epidemiology.48(3):934-44. | No extractable data |
| 789 | Zhao Y, Condon JR, Guthridge S, You J. Living longer with a greater health burden--changes in the burden of disease and injury in the Northern Territory Indigenous population between 1994-1998 and 1999-2003. Australian and New Zealand journal of public health.34:S93-8. | No ethnicity data |
| 790 | Zimmerman SL. States' spending for public welfare and their suicide rates, 1960 to 1995: What is the problem? Journal of Nervous and Mental Disease. 2002;190(6):349-60. | No ethnicity data |
| 791 | Zivin K, Kim HM, McCarthy JF, Austin KL, Hoggatt KJ, Walters H, et al. Suicide mortality among individuals receiving treatment for depression in the veterans affairs health system: Associations with patient and treatment setting characteristics. American Journal of Public Health.97(12):2193-8. | Not general population |

* Articles which were not peer reviewed (e.g. book chapters, letters or comments) are listed as grey literature. We assumed these article types were not peer reviewed.

## Supplementary File 7. Forest plots of absolute rates per 100,000 and rate ratios

### 7.1) Absolute rates per 100,000 by continent amongst moderate-high quality studies

### 7.2) Absolute rates per 100,000 by migrant status amongst moderate-high quality studies

### 7.3) Absolute rates per 100,000 by region of ancestral origin amongst moderate-high quality studies

### 7.4) Rate ratios by continent of residence amongst moderate-high quality studies

### 7.5) Rate ratios by migrant status amongst moderate-high quality studies

### 7.6) Rate ratios by region of ancestral origin amongst moderate-high quality studies

## Supplementary File 8. Sensitivity analysis results: Absolute rates and rate ratios amongst studies of all methodological quality

### Absolute rates

A total of 85 studies of low, moderate, and high quality, reported absolute rates by continent, with highest rates reported in South America (n=6) (38·3 per 100,000; 95% CIs 12·0-122·7), followed by Oceania (n=6) (33·3 per 100,000; 95% CIs 17·3-64·4), Europe (n=20) (15·4 per 100,000; 95% CIs 10·1-23·4), Asia (n=8) (11·0 per 100,000; 95% CIs 4·7-25·6), and North America (n=43) (8·6 per 100,000; 95% CIs 5·8-12·7). High levels of heterogeneity were reported across these studies (I^2^>98%). There were insufficient studies from Africa (n=2) to report on pooled rates, however findings are summarised in forest plots in Supplementary File 6. Both studies reported above average absolute suicide rates.

When examining absolute rates in studies of any methodological quality, 23 studies reported on migrants (11·5 per 100,000; 95% CIs 7·7-17·4). High levels of heterogeneity were reported across these studies (I^2^>99%). There were insufficient studies to report on pooled rates amongst non-migrant groups (n=4), these are summarised in forest plots in Supplementary File 6. Absolute rates amongst non-migrants varied, and were mostly below the average rate, with exception of one study.

When examining absolute rates by ancestral origin in studies of any methodological quality, individuals from Europe reported highest rates (n=8) (25·3 per 100,000; 95% CIs 12·1-52·6), followed by indigenous individuals (n=29) (23·4 per 100,000; 95% CIs 14·6-37·4), individuals from Asia (n=19) (9·1 per 100,000; 95% CIs 5·3-15·6), Black individuals (n=27) (4·3 per 100,000; 95% CIs 3·0-6·3), and Hispanic individuals (n=17) (4·0 per 100,000; 95% CIs 2·1-7·8). There were insufficient studies to report on pooled rates amongst individuals from the Middle East (n=4), with varying rates reported, mostly below the average rate, with exception of one study (see Supplementary File 6). Studies reporting ancestral origin on individuals from North America (n=3), South America (n=1), White (n=3) provided evidence for above average rates of suicide. Whilst studies reporting on ancestral origin from Latino individuals (n=1) and individuals from Africa (n=3) showed below average rates of suicide, with the exception of one study from Africa.

### Rate ratios

A total of 97 studies of low, moderate, and high quality, reported rate ratios by continent (RR 1·2; 95% CIs 0·9-1·5), with highest risk reported in South America (n=6) (RR 7·9; 95% CIs 3·0-21·0), followed by Oceania (n=9) (RR 3·1; 95% CIs 1·7-5·6), Asia (n=7) (RR 1·3; 95% CIs 0·5-3·0). There was no evidence of increased risk in Europe (n=23) (RR 1·0; 95% CIs 0·8-1·3), and North America (n=50) (RR 0·9; 95% CIs 0·6-1·1). High levels of heterogeneity were reported across these studies (I^2^>96%). There were insufficient studies from Africa (n=2) reporting on risk of suicide, therefore we provide estimates on forest plots (see Supplementary File 6), with inconsistent evidence of increased or decreased risk of suicide.

When examining rate ratios by migrant status in studies of any methodological quality, highest risk was estimated in non-migrants (n=5) (RR 1·3; 95% CIs 0·7-2·2). High levels of heterogeneity were reported across these studies (I^2^>95%). There was no evidence of increased risk for any of the other migrant groups.

When examining rate ratios by ancestral origin in studies of any methodological quality, indigenous groups (n=35) reported highest risk (RR 2·8; 95% CIs 2·0-4·0). There was no evidence of increased risk for the following ancestral origin groups: individuals from Asia, Black individuals, Hispanic individuals, and individuals from the Middle East (see Table 3). There were insufficient studies reporting on ancestral origin in individuals from Africa (n=3), South America (n=1), Latino (n=1), White (n=2), and North America (n=2). Throughout the studies, inconsistent evidence was found in relation to increased or decreased suicide risk, as seen in the forest plots in Supplementary File 6.

## Supplementary File 9. Absolute suicide rates per 100,000 and relative risk of suicide amongst individuals from an ethnic minority background in high-quality rating studies

|  | **High-quality studies** | | |  |
| --- | --- | --- | --- | --- |
|  | Rate (95% CI) | I^2^ | No. of studies | |
| **Absolute rates**  **Overall**  **Sex**  Male  Female | 23·9 (12·9-44·0)  34·0 (15·4-74·8) 8·2 (3·3-20·1) | 99·2%  99·3% 97·8% | 15  8 8 | |
|  | Rate ratios (95% CI) | I^2^ | No. of studies | |
| **Rate ratios**  **Overall**  **Sex**  Male  Female | 1·6 (1·0-2·6)  1·5 (0·8-2·8) 1·4 (0·6-3·1) | 98·9%  99·4% 98·9% | 20  11 11 | |

# Supplementary File 10. Absolute suicide rates per 100,000 in individuals from an ethnic minority background by continent, migrant status and region of ancestral origin – multi-level meta-analysis

|  | **Moderate and high-quality studies** | | | | | **All studies** | | | | |
| --- | --- | --- | --- | --- | --- | --- | --- | --- | --- | --- |
|  | Rate (95% CI) | I^2^  Studies | I^2^ Estimates | No. of studies | No. of estimates | Rate (95% CI) | I^2^  Studies | I^2^  Estimates | No. of studies | No. of estimates |
| Overall | 10·8 (7·8-15·0) | 64·8% | 35·2% | 42 | 145 | 11·6 (9·0-14·8) | 72·8% | 27·.1% | 85 | 329 |
| **Sex**  Male  Female | 21·3 (12·7-35·8)  6·3 (3·6-10·8) | 73·6%  65·0% | 26·4%  34·8% | 20 18 | 68  58 | 20·4 (15·6-26·8)  6·7 (5·1-8·8) | 68·1% 65·5% | 31·2%  34·4% | 53 51 | 203  185 |
| **Continent**  Africa  Asia  Europe  N. America  Oceania  S. America | -  -  -  9·2 (6·3-13·3)  -  - | -  -  -  63·9%  -  - | -  -  -  36·1%  -  - | -  -  -  28  -  - | -  -  -  110  -  - | -  9·1 (3·5-23·6)  15·3 (10·7-21·8)  9·0 (6·3-12·9)  17·4 (10·3-29·5)  21·2 (5·2-87·5) | -  68·6%  75·2%  72·7%  99.9%  63·4% | -  31·4%  24·8%  27·2%  0·1%  36·4% | -  8  20  43  6  6 | -  31  85  161  14  11 |
| **Migrant Status**  Migrant  Non-migrant  Indigenous  Not specified | 11·0 (7·5-15·9)  -  -  22·8 (14·4-36·2)  6·2 (4·1-9·4) | 81·6%  -  -  0%  76·9% | 18·4%  -  -  99·4%  23·1% | 41  -  -  18  26 | 100  -  -  19  81 | 11·9 (9·1-15·6)  12·0 (8·0-18·2)  -  29·8 (19·9-44·5)  6·3 (4·4-8·9) | 84·7%  75·2%  -  71·5%  82·7% | 15·2%  24·5%  -  27·9%  17·2% | 85  23  -  27  41 | 237  72  -  31  134 |
| **Ancestral Origin**  Asia  Black  Europe  Hispanic  Indigenous  Middle East  Not clear | 10·9 (7·5-15·6)  7·0 (2·5-19·3)  4·4 (2·9-6·6)  -  6·0 (2·9-12·7)  23·1 (15·3-35·1) -  4·8 (3·0-7·8) | 80·8%  89·9%  53·8%  -  76·7%  0%  -  0% | 19·2%  10·1%  46·0%  -  23·2%  99·2% -  99·9% | 48  9  18  -  10  20 -  13 | 101  20  28  -  18  21  -  14 | 11·6 (8·9-15·2)  8·3 (4·6-14·9)  4·2 (3·0-6·1)  19·8 (10·7-36·9)  4·3 (2·3-7·8)  22·2 (14·1-35·0)  -  7·3 (5·0-10·7) | 83·0%  69·7%  57·3%  77·9%  79·4%  76·8% -  83·4% | 17·0%  30·3%  42·5%  21·9%  20·5%  22·8%  -  16·5% | 85  19  27  10  18  31  -  37 | 230  44  37  29  26  34  -  60 |

Note: Rates are estimated using a two-level random-effects meta-analysis where estimates are nested within studies.

# Supplementary File 11. Rate ratios amongst individuals from an ethnic minority background by continent, migrant status and region of ancestral origin – multi-level meta-analysis

|  | **Moderate and high-quality studies** | | | | | **All studies** | | | | |
| --- | --- | --- | --- | --- | --- | --- | --- | --- | --- | --- |
|  | Rate Ratios (95% CI) | I^2^  Studies | I^2^  Estimates | No. of studies | No. of estimates | Rate Ratios (95% CI) | I^2^  Studies | I^2^  Estimates | No. of studies | No. of estimates |
| Overall | 1·2 (0·9-1·7) | 90·0% | 9·9% | 51 | 115 | 1·1 (0·9-1·4) | 86·0% | 13·9% | 97 | 286 |
| **Sex**  Male  Female | 1·2 (0·8-1·9) 1·3 (0·8-2·2) | 92·7%  86·4% | 7·2%  13·1% | 27 25 | 61  52 | 1·1 (0·9-1·4) 1·2 (1·0-1·6) | 75·0% 79·9% | 24·5%  19·6% | 63 67 | 355  333 |
| **Continent**  Africa  Asia  Europe  N. America  Oceania  S. America | -  -  -  0·8 (0·6-1·1)  3·3 (1·9-5·9)  - | -  -  -  86·8%  49·0%  - | -  -  -  13·1%  49·0%  - | -  -  -  35  6  - | -  -  -  89  6  - | -  1·3 (0·5-3·2)  0·9 (0·7-1·2)  0·8 (0·6-1·1)  2·6 (1·3-5·2)  7·7 (2·9-21·0) | -  69·8%  54·2%  88·1%  32.0%  95·4% | -  30·2%  44·2%  11·8%  66·8%  4·1% | -  7  23  50  9  6 | -  20  100  124  13  8 |
| **Migrant Status**  Migrant  Non-migrant  Indigenous  Not specified | 1·2 (0·9-1·7)  -  -  2·8 (2·0-4·0)  0·6 (0·4-0·7) | 91·0%  -  -  0%  73·0% | 9·0%  -  -  99·3%  26·9% | 50  -  -  25  29 | 112  -  -  27  85 | 1·3 (1·0-1·6)  0·9 (0·7-1·3)  1·3 (0·6-3·2)  3·2 (2·2-4·4)  0·6 (0·5-0·7) | 89·3%  78·5%  70·3%  68·8%  71·4% | 10·6%  21·0%  27·8%  30·5%  28·4% | 74  26  5  34  45 | 180  85  21  40  140 |
| **Ancestral Origin**  Asia  Black  Europe  Hispanic  Indigenous  Middle East  Not clear | 1·2 (0·9-1·7)  0·8 (0·4-1·8)  0·4 (0·4-0·5)  -  0·5 (0·4-0·6)  2·8 (2·0-3·9)  -  0·7 (0·4-1·1) | 89·8%  87·0%  41·1%  -  19·3%  0%  -  99·9% | 10·1%  13·0%  58·3%  -  79·3  99·3%  -  0% | 81  9  21  -  10  25  -  16 | 111  18  31  -  18  27  -  17 | 1·1 (0·9-1·4)  0·9 (0·5-1·6)  0·5 (0·4-0·6)  0·9 (0·6-1·4)  0·4 (0·4-0·5)  2·9 (2·0-4·0)  0·4 (0·3-0·5)  0·8 (0·6-1·0) | 86·0%  81·3%  53·1%  65·6%  6·6%  65·8%  90·3%  78·0% | 13·9%  18.6%  46·3%  34·0%  92·5%  33·5%  0%  21·8 | 97  19  30  9  18  35  6  44 | 268  39  40  44  26  40  8  71 |

Note: Rate ratios are estimated using a two-level random-effects meta-analysis where estimates are nested within studies.

# Supplementary File 12. Funnel plots


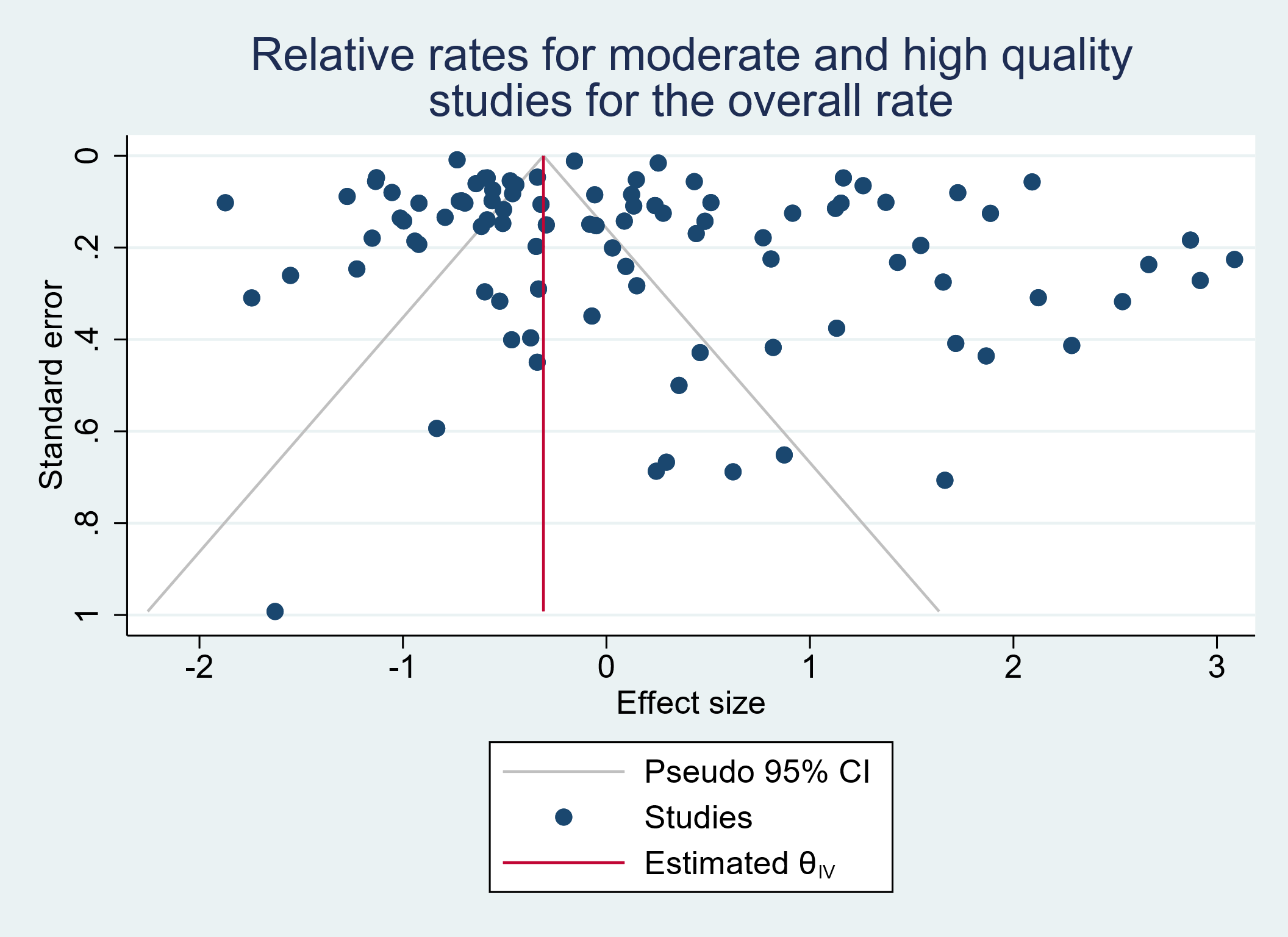

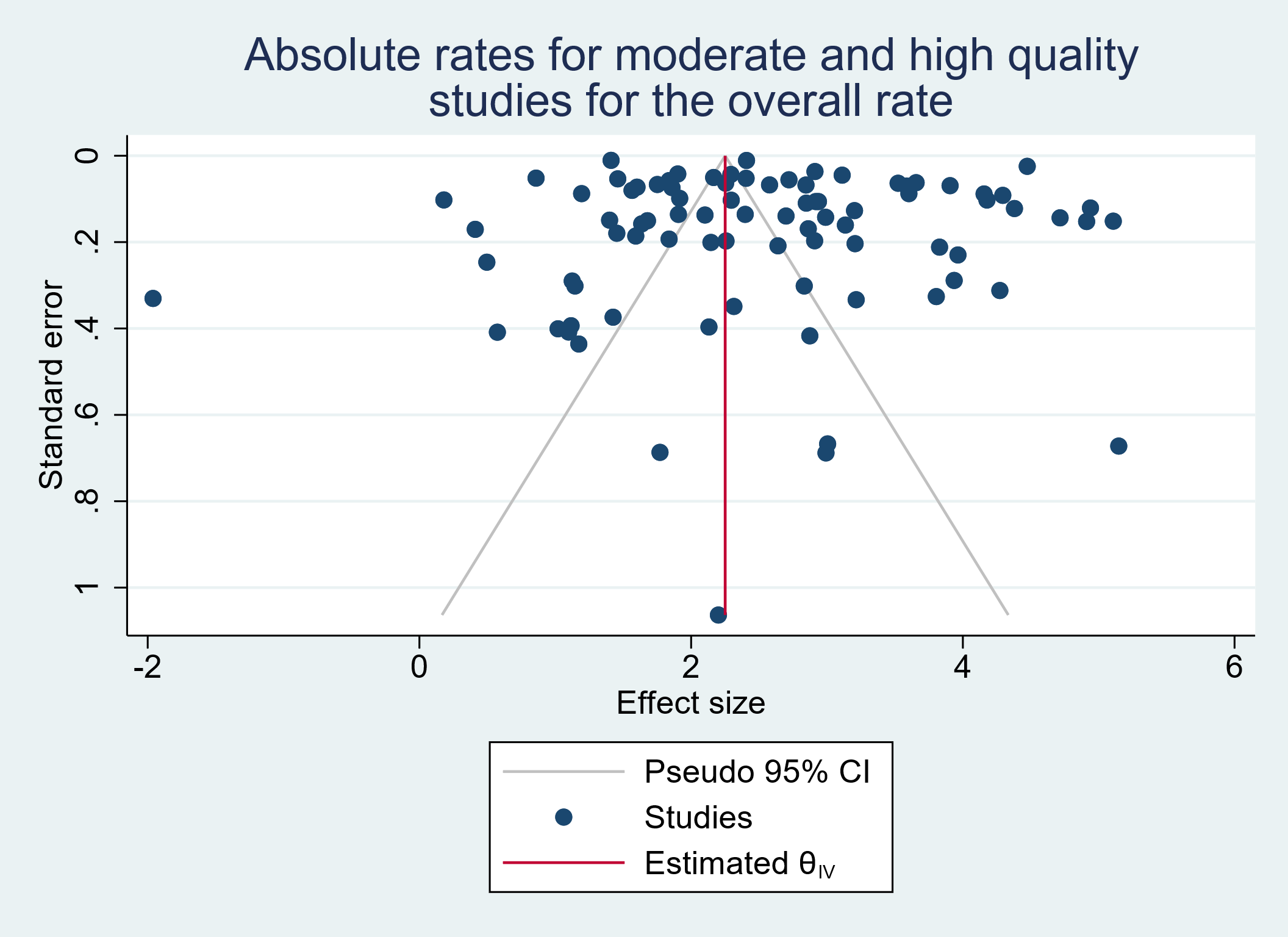

Supplement: Supplementary file 1 [file mmc1.docx]
